# Epigenetics of Aging and Alzheimer’s Disease: Implications for Pharmacogenomics and Drug Response

**DOI:** 10.3390/ijms161226236

**Published:** 2015-12-21

**Authors:** Ramón Cacabelos, Clara Torrellas

**Affiliations:** 1 EuroEspes Biomedical Research Center, Institute of Medical Science and Genomic Medicine, 15165-Bergondo, Corunna, Spain; serviciodocumentacion@euroespes.com; 2 Chair of Genomic Medicine, Camilo José Cela University, 28692-Madrid, Spain

**Keywords:** aging, Alzheimer’s disease, brain disorders, epigenetics, epigenetic drugs, drug resistance, pharmacogenomics, pharmacoepigenomics

## Abstract

Epigenetic variability (DNA methylation/demethylation, histone modifications, microRNA regulation) is common in physiological and pathological conditions. Epigenetic alterations are present in different tissues along the aging process and in neurodegenerative disorders, such as Alzheimer’s disease (AD). Epigenetics affect life span and longevity. AD-related genes exhibit epigenetic changes, indicating that epigenetics might exert a pathogenic role in dementia. Epigenetic modifications are reversible and can potentially be targeted by pharmacological intervention. Epigenetic drugs may be useful for the treatment of major problems of health (e.g., cancer, cardiovascular disorders, brain disorders). The efficacy and safety of these and other medications depend upon the efficiency of the pharmacogenetic process in which different clusters of genes (pathogenic, mechanistic, metabolic, transporter, pleiotropic) are involved. Most of these genes are also under the influence of the epigenetic machinery. The information available on the pharmacoepigenomics of most drugs is very limited; however, growing evidence indicates that epigenetic changes are determinant in the pathogenesis of many medical conditions and in drug response and drug resistance. Consequently, pharmacoepigenetic studies should be incorporated in drug development and personalized treatments.

## 1. Introduction

Epigenetics is the molecular phenomenon by which phenotypic changes are transmitted from one generation to another with no apparent alterations in structural DNA. Epigenetic mechanisms (DNA methylation, histone modifications, and microRNAs (miRNAs) regulation) are among the major regulatory elements responsible for the control of metabolic pathways at the molecular level. Epigenetic modifications regulate gene expression transcriptionally, whereas miRNAs suppress gene expression post-transcriptionally [1].

Epigenetic reprogramming is present in vertebrate genomes during development and disease. In some asexual species, stable transmission of DNA methylation, transcriptomes and phenotypes from parent to clonal offspring has been demonstrated. Furthermore, clonal genotypes from natural populations show habitat-specific DNA methylation [2]. The methylation process varies spatially across the genome, with a majority of the methylated sites mapping to intragenic regions [3]. Mitochondrial DNA (mtDNA) is also under epigenetic regulation [4]. Distinctly methylated genes identified in different human populations may reflect the influence of DNA methylation on phenotype differences (*i.e.*, susceptibility to certain diseases and pathogens, and response to drugs and xenobiotic agents).

Epigenetic regulation is a molecular mechanism linking exposures during the course of life to long-term health. Epigenetic status is modified by environmental exposures such as nutrition, social status, chemical and emotional environment, pregnancy conditions, infertility, contraception, and different modalities of pharmacological intervention. Epigenetic status is also influenced by genotype, and genetic variation in genes encoding a plethora of enzymes and proteins [5]. Furthermore, DNA methylation contributes to natural human variation [4]. Epigenetic modifications are reversible and can potentially be targeted by pharmacological and dietary interventions [6]. A series of epigenetic drugs have been developed (Table 1), and some of these compounds have been approved by the Food and Drug Administration (FDA) for the treatment of neoplastic processes [7,8,9].

**Table 1:** Classification of selected epigenetic drugs.

**DNA Methyltransferase Inhibitors**
**Nucleoside analogs:** 5-Aza-2′-deoxycytidine (Decitabine); 5-Azacytidine (Azacitidine)
**Small molecules:** Hydralazine; Procainamide; RG108 [2-(1,3-dioxo-1,3-dihydro-2H-isoindol-2-yl)-3-(1H-indol-3-yl)propanoic acid]
**Natural products:** Curcumin derivatives: RG-108, SGI-1027; Psammaplins; Tea polyphenols: Epigallocatechin-3-gallate; Catechins: Catechin, Epicatechin; Bioflavonoids: Quercetin, Genistein, Fisetin
**Antisense oligonucleotide inhibitors**
**ncRNAs (miRNAs)**
**Histone deacetylase (HDAC) inhibitors**
**Short-chain fatty acids:** Sodium butyrate; Sodium phenyl butyrate; Valproic acid; Pivaloyloxymethyl butyrate (AN-9, Pivanex)
**Hydroxamic acids:** Suberoylanilide hydroxamic acid (SAHA, Vorinostat); Oxamflatin; Pyroxamide; TSA; CBHA; Derivatives of the marine sponge *Psammaplysilla purpurea*: NVP-LAQ824, NVP-LBH589; LBH-589 (Panobinostat); ITF2357 (Givinostat); PXD101 (Belinostat); JHJ-26481585; CHR-3996; CHR-2845; PCI-24781
**Cyclic peptides:** Romidepsin (Depsipeptide, FR901228); Apicidin; CHAPS; Trapoxin A and B; Chlamydocin; HC toxin; Bacterial FK228
**Benzamides:** MS-275 (Entinostat); CI-994; RGFP136; MGCD0103 (Mocetinostat); Compound 60
**Ketones:** Trifluoromethyl ketone
**Sirtuin modulators:** Sirtuin inhibitors: Nicotinamide/niacinamide, Suramin, AGK-2, Sirtinol, Salermide, MS3, Splitomycin, Cambiol, SEN-196, Dihydrocoumarin, Tenovin, UVI5008; Sirtuin activators: Resveratrol, SRT-501, SRT-1460, SRT-1720, SRT-2183, GSK-184072, Quercetin, Piceatannol
**Miscellaneous compounds:** 3-Deazaneplanocin A (DZNep); Tubacin; EVP-0334; 6-([^18^F]Fluoroacetamido)-1-hexanoicanilide; Quinazolin-4-one derivatives: (*E*)-3-(2-Ethyl-7-fluoro-4-oxo-3-phenethyl-3,4-dihydroquinazolin-6-yl)-*N*-hydroxyacrylamide, *N*-Hydroxy-3-(2-methyl-4-oxo-3-phenethyl-3,4-dihydro-quinazolin-7-yl)-acrylamide
**Histone acetyltransferase modulators**
**Histone acetyltransferase inhibitors:** Curcumin (Diferuloylmethane); Lys-CoA; H3-CoA-20; Anacardic acid; Garcinol
**Histone aceyltransferase activators:** *N*-(4-Chloro-3-trifluoromethyl-phenyl)-2-ethoxy-6-pentadecyl-benzamide; Pentadecylidenemalonate 1b (SPV-106)
**Histone methyltransferase inhibitors**
**Lysine methyltransferase inhibitors:** *S*-Adenosylmethionine (SAMe); SAMe analogs; Chaetocin; BIX-01294; BIX-01338; UNC0224; EZH2 (KMT6) inhibitors: Deazaneplanocin A
**Arginine methyltransferase inhibitors:** AMI-1
**Histone demethylase inhibitors**
**LSD1 inhibitors:** Tranylcypromine; Parnate; (*S*)-4-(2-(5-(Dimethylamino)naphthalene-1-sulfonamido)-2-phenylacetamido)-*N*-hydroxybenzamide (D17)
**Non-coding RNAs**
**miRNAs**
**RNAi**
**Other potential epigenetic treatments**
**Small molecule inhibitors to chromatin-associated proteins:** DOT1L histone methyltransferase inhibitors: EPZ004777, EPZ-5676, SGC0946; EZH2 histone methyltransferase inhibitors: GSK126, GSK343, EPZ005687, EPZ-6438, EI1, UNC1999; G9A histone methyltransferase inhibitors: BIX1294, UNC0321, UNC0638, NC0642, BRD4770; PRMT3 histone methyltransferase inhibitors: 14u; PRMT4 (CARM1) histone methyltranferase inhibitors: 17b, MethylGene; LSD1 histone demethylase inhibitors: Tranylcypromine, ORY-1001; BET histone demethylase inhibitors: JQ1, IBET762, IBET151, PFI-1; BAZ2B histone demethylase inhibitors: GSK2801; L3MBTL1 chromodomain inhibitors: UNC669; L3MBTL3 chromodomain: UNC1215; Bromodomain inhibitors: LP99, RVX-208; Chromodomain inhibitors
**Small molecules for somatic cell reprogramming**
**Chaperones** **(sHSPs)**
**IGFBP7 inhibitors**
**Nutraceuticals**
**Dietary regimes:** Vitamins: Folic acid, Vitamin B, Vitamin C, Vitamin D, Vitamin E; Natural products

BAZ2B: Bromodomain adjacent to zinc finger domain protein 2B; BET: bromo and extra terminal; CARM1: Coactivator-associated arginine methyltransferase 1; CBHA: m-Carboxycinnamic acid bis-hydroxamide; CHAPS: Cyclic hydroxamic acid-containing peptides; DOT1L: DOT1-like histone H3K79 methyltransferase; EZH2: enhancer of zeste 2 polycomb repressive complex 2 subunit; G9A: Euchromatic Histone-Lysine N-Methyltransferase 2; HC: Helminthosporium carbonum; IGFBP7: Insulin-like growth factor binding protein 7; L3MBTL1: Lethal(3)malignant brain tumor-like protein 1; L3MBTL3: Lethal(3)malignant brain tumor-like protein 3; LSD1: Lysine (K)-specific demethylase 1; PRMT3: Protein Arginine Methyltransferase 3; sHSPs: small heat shock proteins; TSA: Trichostatin A.

Multiple epigenetic changes have been reported in different tissues with aging. Epigenetic factors influence life span in several species. Major problems of health, such as cardiovascular disorders, cancer and neurodegeneration, are age-dependent processes in which epigenetic alterations also play a pathogenic role. Most of these complex disorders are the result of multiple defects distributed across the human genome together with the interaction of environmental factors and epigenetic phenomena [8,10]. Some of these medical conditions are susceptible to epigenetic intervention with epigenetic drugs. Interventions targeting epigenetic regulation might be effective in treating a range of age-related neurodegenerative disorders [8,9]. The downstream synaptic protein response to some epigenetic drugs is experience-dependent, and this plasticity is disrupted in the aged hippocampus. Epigenetic intervention may modify the hippocampal transcriptome, potentially reversing age-related cognitive dysfunction [11]. Consequently, epigenetics is, therefore, of considerable translational significance to the field of neuroprotection [12]. In addition, the efficacy and safety of both epigenetic drugs and other medications are closely associated with the efficiency of the pharmacogenetic process in which the gene clusters involved in the pharmacogenetic network (pathogenic, mechanistic, metabolic, transporter, pleiotropic genes) are also under the influence of epigenetic changes; therefore, the pharmacoepigenetic machinery may determine the therapeutic outcome (drug efficacy and safety) [13]. The redundancy and promiscuity of this complex system regulating drug effects and toxicity is a scientific challenge of paramount importance for the pharmaceutical industry and the medical community in the coming years [9].

## 2. The Epigenetic Machinery

### 2.1. DNA Methylation/Demethylation

DNA methylation occurs when methyl groups are incorporated into cytosine molecules by DNA methyltransferases (DNMTs), forming 5-methylcytosine. This process contributes to the suppression of transcription. About 70% of CpG dinucleotides within the human genome are methylated. CpG islands in promoter regions of genes are defined as 200 bp regions of DNA where the GC content is greater than 60%. DNA methylation inhibits transcription by interfering with the binding of transcription factors to recognition sites on promoters or by recruiting and binding transcriptional repressors, and altering chromatin structure. The 5-Methylcytosines (5mC) can also be oxidized to form 5-hydroxymethylcytosine (5hmC) to reduce the interaction of DNA with DNA-binding proteins [14]. CpG methylation may also cause a dual effect on transcription, repressing transcription when CpG methylation occurs at the promotor level or promoting transcription when CpG methylation affects the gene sequence [15]. The transfer of methyl groups from *S*-adenosyl-methionine (SAMe) to cytosine in CpGs is catalyzed by a family of DNA methyltransferases (DNMTs), which in mammals are represented by two *de novo* DNMTs (DNMT3A, DNMT3B) and a maintenance DNMT (DNMT1) that is expressed in neurons. At least three enzyme families are involved in DNA demethylation: (i) the ten-eleven translocation (TET) family, mediating the conversion of 5mC into 5hmC; (ii) the AID/APOBEC family, acting as mediators of 5mC or 5hmC deamination; and (iii) the BER (base excision repair) glycosylase family involved in DNA repair [14]. LSD1 (Lysine-Specific Demethylase 1, KDM1A, AOF2) is a histone modifier involved in transcriptional repression, forming a stable core complex with the corepressor of RE1-silencing transcription factor (CoREST) and histone deacetylases (HDAC1/2).

### 2.2. Histone Modifications

Histones are formed by a central globular domain and an N-terminal tail with multiple sites for modification of nucleosomal organization, leading to ATP-dependent chromatin remodeling complexes and post-translation amino acid modifications on histone tails (histone acetylation, methylation, phosphorylation, sumoylation, ubiquitylation, glycosylation, ADP ribosylation, biotinylation) [14,16]. Histone modifications (HMs) influence transcription, DNA repair and DNA replication. Histone acetylation is achieved by the action of histone acetyltransferase (HAT). HAT incorporates an acetyl group to a lysine residue, resulting in chromatin/transcriptional activation. Histone deacetylases (HDACs) remove acetyl groups, and histone deacetylation promotes chromatin inactivation and transcriptional repression [16,17].

### 2.3. Chromatin Remodeling

Chromatin regulators (CRs) mediate HMs to adjust chromatin structures and functions. A stable heterochromatin is essential for silencing transposable elements (TEs) and maintaining genome integrity. ATP-dependent chromatin remodeling complexes use ATP hydrolysis to move, destabilize, eject or restructure nucleosomes, allowing the accessibility of transcription factors to DNA. These complexes can be classified into four families: (i) the SWI/SNF (switching defective/sucrose nonfermenting) family; (ii) the ISWI (imitation SWI) family; (iii) the CHD (chromodomain, helicase, DNA binding) family; and (iv) the INO (inositol requiring 80) family [18]. Their transcriptional effects (activation or repression) depend upon the recruitment of coactivators or corepressors [14].

### 2.4. Post-Translational Histone Modifications

Post-translational changes on histone tails (acetylation, ubiquitylation, or sumoylation at K (lysine) residues, methylation at K, R (arginine) or H (histidine) residues, and phosphorylation at S (serine), T (threonine) or Y (tyrosine) residues) affect transcription, DNA replication and DNA repair [14]. Histone acetylation is catalyzed by five families of histone lysine acetyltransferases (KATs) (KAT2A/GCN5, KAT2B/PCAF, KAT6-8, CREBBP/CBP, EP300) [19]. Histone acetylation is associated with transcriptional activation and open chromatin conformation, and histone deacetylation is involved in transcriptional repression and closed chromatin structure. According to their homology to yeast, 18 mammalian HDACs are classified into four classes (classes I, II, III, IV). Class I HDACs (HDAC1, 2, 3, and 8) are nuclear proteins; HDAC1 and HDAC2 are often found in transcriptional corepressor complexes (SIN3 transcription regulator family member A (SIN3A), Nucleosome remodeling deacetylase (NuRD), CoREST), and HDAC3 is found in other complexes (silencing mediator of retinoid and thyroid hormone receptor/nuclear receptor corepressor (SMRT/N-CoR)); class II HDACs are subdivided into class IIa (HDAC4, 5, 7, and 9), and IIb (HDAC6 and 10), which are located in the nucleus-cytoplasm interface and in the cytoplasm, respectively. Class III HDACs belong to the sirtuin family, with nuclear (SIRT1, 2, 6, 7), mitocondrial (SIRT3, 4, 5), or cytoplasmic (SIRT1, 2) localization. Class IV HDAC (HDAC11) is a nuclear protein [14,16,20,21].

### 2.5. Non-Coding RNAs

Long non-coding (lnc) RNAs are defined as non-protein-coding RNAs, distinct from housekeeping RNAs (tRNAs, rRNAs, and snRNAs) and independent from small RNAs with specific molecular processing machinery (micro- or piwi-RNAs) [22]. Over 95% of the eukaryotic genome is transcribed into non-coding RNAs (ncRNAs) and less than 5% is translated. LncRNA-mediated epigenetic regulation depends mainly on lcnRNA interactions with proteins or genomic DNA via RNA secondary structures, and some lncRNAs rely on Watson-Crick base pairing for functional activity [23]. ncRNAs are classified by size into two categories: (i) small RNAs (<200 nucleotides) including (a) structural RNAs (ribosomal (rRNA), transfer (tRNA), small nuclear RNAs (snRNA)) and (b) regulatory RNAs (microRNAs (miRNA), small interfering RNAs (siRNA), small nuclear RNAs (snRNA), piwi-interacting RNAs (piRNA), splice junction-associated RNAs); and (ii) long RNAs (lncRNAs) (>200 nucleotides), present in >8000 loci in the human genome, which include large intergenic non-coding RNAs (lincRNA), natural antisense transcripts (NATs), non-coding RNA expansion repeats, promoter-associated RNAs (PARs), enhancer RNAs (eRNAs), small activating RNAs (saRNAs, RNAa) [14,24,25].

Small ncRNAs (miRNAs, siRNAs, piRNAs) show mature forms of 20–30 nucleotides (nt) that associate with members of the Argonaute (AGO) superfamily of proteins, the central effectors of RNA interference (RNAi) pathways. miRNAs and siRNAs are post-transcriptional gene silencers, inducing transcript degradation and blocking translation [24]. miRNAs repress translation with RISC (RNA-induced silencing complex) and induce mRNA degradation by binding to the 3′ untranslated region (3′UTR). Other miRNAs may enhance mRNA translation and induce gene expression by binding to the promoter of the target gene. ncRNAs are essential in the regulation of epigenetic mechanisms (silencing of transposable elements, gene expression control, X-chromosome inactivation, DNA imprinting, DNA methylation, histone modifications). piRNAs are essential for fertility, associating with the PIWI clade of Argonautes to silence transposons in the germline [24]. Changes in histones are accompanied by RNA activation (RNAa) around the target promoter, and DNA methylation does not appear to be affected by RNAa [26], although RNA-directed DNA methylation (RdDM) and RNA-induced transcriptional silencing (RITS) phenomena have been reported [23]. Endogenous small RNA-mediated epigenetic gene regulation involves miRNA-induced RNAa and miRNA-induced transcriptional gene silencing (TGS) [23].

Long non-coding RNAs (lncRNAs) function as adaptors that link specific chromatin loci with chromatin-remodeling complexes and transcription factors. lncRNAs can act in cis or trans to guide epigenetic-modifier complexes to distinct genomic sites, or act as scaffolds which recruit multiple proteins simultaneously, thereby coordinating their activities [26]. miRNAs are small ncRNAs that inhibit the expression of target mRNAs by reducing both their stability and translation rate. miRNAs are involved in the regulation of gene expression at the epigenetic level, and epigenetic regulators are strongly enriched among the predicted targets of miRNAs, contributing to pluripotency, development and somatic cell reprogramming [27]. Similar to protein-coding genes, miRNAs are also susceptible to epigenetic modulation. Several miRNAs have been shown to be affected by DNA methylation. Enhancer of zeste homolog 2 (EZH2) and HDACs were recently identified as critical histone modifiers of deregulated miRNAs in cancer and can be recruited to a miRNA promoter by transcription factors such as v-myc avian myelocytomatosis viral oncogene homolog (MYC) [28].

## 3. Age-Related Epigenetics

Epigenetic changes in genes associated with age affect life expectancy and longevity [29]. Altered DNA methylation patterns may account for phenotypic changes associated with human aging [30,31]. Age- and tissue-dependent DNA hypo- and hypermethylation has been reported [16,30,31]. It appears that global loss of DNA methylation predominates in aged cells. DNMT1, which maintains DNA methylation of CpGs, decreases with age [32]. In contrast, some loci have been found hypermethylated with age (e.g., estrogen receptor, interferon γ, insulin-like growth factor II, promoters of tumor-suppressor genes such as lysyl oxidase (*LOX*), *p16INK4a*, runt-related transcription factor 3 (*RUNX3*), and TPA-inducible gene 1 (*TIG1*)) [14]. In 1006 blood DNA samples of women (35–76 years) from the Sister Study, Xu and Taylor [33] found 7694 (28%) CpGs associated with age, indicating that at least 749 “high-confidence” age-related CpG (arCpGs) sites are present in normal blood. About 71%–91% of increasingly methylated arCpGs (IM-arCpGs) were over-methylated in a wide variety of tumor types. IM-arCpGs sites occurred almost exclusively at CpG islands and were disproportionately marked with the repressive H3K27me3 histone modification. It appears that as cells acquire methylation at age-related sites, they have a lower threshold for malignant transformation, which may explain, in part, the increase in cancer incidence with age.

Age-related changes in DNA methylation occurring in blood leukocytes during early childhood may reflect epigenetic maturation. Some of these changes involve gene networks of critical relevance in leukocyte biology. Susceptibility loci for complex inflammatory diseases (*IRF5*, *NOD2* and *PTGER4*) and genes encoding histone modifiers and chromatin remodeling factors (*HDAC4*, *KDM2A*, *KDM2B*, *JARID2*, *ARID3A* and *SMARCD3*) undergo DNA methylation changes in leukocytes during early childhood [34,35]. McClay *et al.* [36] performed a methylome-wide association study (MWAS) of aging in whole blood DNA from 718 individuals, aged 25–92 years. They sequenced the methyl-CpG-enriched genomic DNA fraction, averaging 67.3 million reads per subject, to obtain methylation measurements for the ~27 million autosomal CpGs (4,344,016 CpG blocks) in the human genome. Forty-two differentially methylated regions (DMRs) were hypomethylated and 28 were hypermethylated with age. Hypermethylated DMRs overlaped with CpG islands and shores. Hypomethylated DMRs were preferentially found in regions associated with polycomb/regulatory proteins (EZH2) or histone modifications (H3K27ac, H3K4m1, H3K4m2, H3K4m3 and H3K9ac). Among genes implicated by the top DMRs were protocadherins, homeobox genes, mitogen-activated protein kinases (*MAPKs*), ryanodine receptors, and genes with potential relevance for age-related disease.

In an interesting study by Yang *et al.* [37] using genome-wide DNA methylation data from 740 postmortem brains, the authors interrogated 420,132 CpG sites across the genome in a cohort of individuals with ages from 66 to 108 years old, a range of ages at which many neuropathologic indices become quite common, and found 8156 age-associated CpGs. The number of age-associated CpGs dropped by more than 10% following adjustment for sex. After adjusting for common neuropathologies, the total number of age-associated CpGs was reduced by approximately 40% compared to the sex-adjusted model. According to these data, the association of methylation changes in the brain with age is inflated if one does not account for age-related brain pathologies.

Histone modifications are also observed with aging. Histone acetylation decreases and phosphorylation increases with age [38]. H4K20me and H3K36me3 decrease in the brain of old senescence-accelerated-prone mice (SAMP8) and H3K27m^3^, H3K79me, and H3K79me^2^ increase in these aged mouse brains [39]. The silent information regulator 2 (Sir2) in yeast and its mammalian orthologs, sirtuin 1–7 (SIRT1–7), are histone-modifying enzymes which tend to be dowregulated in aging, especially SIRT1. Activation of sirtuins may extend lifespan-modulating calorie restriction mechanisms [40] and promote healthy aging, delaying the onset of neurodegenerative processes [41]. In the epidermis, aging is associated with a limited destabilization of the epigenome at gene-regulatory elements [42]. Wound treatment with sirtuin activators and class I HDAC inhibitors induces keratinocyte proliferation and enhances healing via a nitric oxide (NO)-dependent mechanism. Acetylation of α-tubulin and histone H3 Lysine 9 may activate cell function and gene expression to foster tissue repair. The direct activation of P300/CBP-associated factor (PCAF) by the histone acetylase activator pentadecylidenemalonate 1b (SPV-106) induces lysine acetylation in wounds. Alterations in PCAF and/or GCN5 family acetylases may affect skin repair under different pathological conditions [43].

Loss of transcriptional regulation is a phenomenon related to shortened life span. Lack of H3K36 methylation is proportional to the increased transcription of genes in old cells with shorter life span, and deletion of K36me2/3 demethylase Rph1 increases H3K36me3 in these genes, suppresses transcript initiation, and extends life span. Epigenetic misregulation causes a loss of transcriptional precision with detrimental effects on life span in aging cells. Restoring transcriptional fidelity may reverse accelerated aging [44].

Age-associated differentially methylated regions (aDMRs) and regulatory signatures in the promoters of age-associated genes (aGENs) share a common Polycomb Repressive Complex 2 (PRC2) signature (EZH2, SUZ12 polycomb repressive complex 2 subunit (SUZ12), CCCTC-binding factor (CTCF)) binding sites, repressive H3K27me3, and activating H3K4me1 histone modification), and a “poised promoter” chromatin state. This signature is depleted in RNA Polymerase II–associated transcription factor binding sites, activating H3K79me2, H3K36me3, and H3K27ac marks, and an “active promoter” chromatin state. The PRC2 signature is associated with aDMRs hypermethylated with age, while hypomethylated aDMRs are associated with enhancers. aGENs are associated with the PRC2 signature independently of the directionality of gene expression changes. The PRC2 signature represents an epigenomic mark associated with changes in DNA hypermethylation and gene expression in aging [45].

Trimethylation of Lys36 on histone 3 (H3K36me3) in *Caenorhabditis elegans* cells revealed that H3K36me3 influences gene expression stability with effects on longevity. Inactivation of methyltransferase met-1 results in decreased H3K36me3 marks, increased mRNA expression change with age, and shortened life span, indicating that H3K36me3 modulates age-dependent gene expression stability and longevity [46].

There is a correlation between changes in miRNA expression and aging. miRNA lin-4 regulates life span in *C. elegans*; several miRNAs (miRNAs-34, -669c, -709, -93, -214) were found to be upregulated with age, while others (miRNAs-103, -107,-128, -130a, -155, -24, -221, -496, -1538, -17, -19b, -20a, -106a) appeared downregulated in peripheral tissues [47,48]. Seventy miRNAs were found to be upregulated in the aging brain; 27 of these miRNAs may target genes of mitocondrial complexes III, IV, and F_0_F_1_-ATPase involved in oxidative phosphorylation and reduced expression in aging [49].

Oncogenic insults activate oncogene-induced senescence (OIS) as a tumor suppressor mechanism. Montes *et al.* [50] identified upregulation of lncRNA MIR31 host gene (*MIR31HG*) as in OIS. Knockdown of MIR31HG promotes a strong p16(INK4A)-dependent senescence phenotype. MIR31HG interacts with both INK4A and MIR31HG genomic regions and with Polycomb group (PcG) proteins, and MIR31HG is required for PcG-mediated repression of the *INK4A* locus. A functional enhancer located between MIR31HG and INK4A becomes activated during OIS and interacts with the MIR31HG promoter. A negative correlation between MIR31HG and p16(INK4A) expression levels was observed in melanoma cells, suggesting a role for this transcript in cancer.

Accelerated telomere shortening may cause cancer via chromosomal instability. Age-related blood telomere length (BTL) attrition is faster in cancer cases, with an age-adjusted BTL attrition decelerating in the proximity of diagnosis [51].

## 4. Pathogenic Epigenetics

Epigenomic modifications are observed in physiological and pathological conditions; of major importance are those associated with age-related processes and with major problems of health such as cardiovascular disorders, obesity, cancer, inflammatory processes, asthma and allergy, and brain disorders. Pharmaceuticals, pesticides, air pollutants, industrial chemicals, heavy metals, hormones, nutrition, and behavior can change gene expression through a broad array of gene regulatory mechanisms (gene translocation, histone modifications, DNA methylation, DNA repair, transcription, RNA stability, alternative RNA splicing, protein degradation, gene copy number, and transposon activation) [52]. Disease-associated genetic variation interferes with miRNA-mediated regulation by creating, destroying, or modifying miRNA binding sites. miRNA-target variability is a ubiquitous phenomenon in the adult human brain, influencing gene expression in physiological and pathological conditions. One of the major roles of lncRNAs in the nucleus is the regulation of gene expression at the transcriptional level via histone or DNA modification [53].

Epigenetic aberrations are a typical paradigm of the oncogenic process in some types of cancer. Myelodysplastic syndromes (MDS) are characterized by chronic cytopenias and dysplasia, with variable progression to acute myeloid leukemia (AML). Aberrant methylation of tumor suppressor gene promoters is a potential driver of MDS pathogenesis. Recurrent somatic mutations in genes encoding proteins involved in DNA methylation and demethylation and in covalent histone modifications are present in myeloid malignancies and MDS. Hypomethylating agents are the therapeutic pillars of advanced MDS [7,54] (Table 2).

Colorectal cancer (CRC) evolves through a multistage process with progressive accumulation of mutations and chromatin aberrations in the promoters of tumor suppressors and oncogenes. CRCs enhibit thousands of abnormally methylated genes. Specific histone modifications and DNA methylation regulate gene expression in CRC pathogenesis [55]. DNA methylation can be used as a biomarker of disease, especially in different types of cancer, such as colon cancer. Sporadic CRC is a consequence of the accumulation of genetic and epigenetic alterations that result in the transformation of normal colonic epithelial cells to adenocarcinomas. Some hypermethylated (HM) and unmethylated (UM) genes may be useful epigenetic markers for non-invasive CRC screening. For instance, the five genes that have been reported to be UM in control tissues are *MLH1* (71.7%), *DKK2* (69.6%), *CDKN2A* (68.4%), *APC* (67.5%) and *hsa-mir-342* (67.4%), whereas *RUNX3* (58.9%), *PCDH10* (55.5%), *SFRP5* (52.1%), *IGF2* (50.4%) and *Hnf1b* (50.0%) are HM in CRC cases [56].

Profound methylation of CpG islands constitutes a distinct molecular subtype of colorectal cancer (CRC). The frequencies of methylation in CRC vary according to clinico-pathological characteristics, including sex. Concurrent methylation in *NEUROG1* and *CDKN2A* is associated with poor survival in CRC treated with adjuvant FOLFOX [57].

Melanoma is a genetically heterogeneous cancer with great plasticity and frequent resistance to antineoplastic treatments. In patients with melanoma, over 20% of all non-silent mutations affect an epigenetic regulator. The *BRAF*, *MECOM*, *NRAS*, *TP53*, *MLL2*, and *CDKN2A* genes are frequently mutant in melanoma. About 30% of the most frequently mutant genes encode epigenetic regulators: (i) enzymes involved in histone modification (*MECOM*, *MLL2*, *SETD2*); (ii) chromatin remodeling (*ARID1B*, *ARID2*); and (iii) DNA methylation and demethylation enzymes (*TET2*, *IDH1*). Over 90% of patients with melanoma are carriers of at least one mutation in an epigenetic regulator, especially *MLL2* (100%) and *MECOM* (82.6%) [58].

The oxidative DNA demethylase alkB homolog 3, α-ketoglutarate-dependent dioxygenase (ALKBH3) targets single-stranded DNA (ssDNA) to perform DNA alkylation damage repair. ALKBH3 becomes upregulated in tumorigenesis and proliferation. ALKBH3 binds to transcription-associated locations (promoter-proximal paused RNA polymerase II enhancers). ALKBH3 binds to the transcription initiation sites of active gene promoters which are characterized by high levels of transcriptional regulators (transcription factors, the mediator complex, cohesin, histone modifiers, active histone marks). ALKBH3 is an intrinsic DNA repair protein that suppresses transcription-associated DNA damage at highly-expressed genes and thereby plays a role in maintaining genomic integrity in ALKBH3-overexpressing cancer cells [59].

Other examples of pathogenic epigenetics are represented by obesity or type 2 diabetes [60,61,62]. Recent studies indicate that changes in DNA methylation of the insulin-like growth factor binding protein-1 (IGFBP1) gene are associated with type 2 diabetes, suggesting that increased IGFBP1 DNA methylation and decreased IGFBP1 serum levels are features of short-duration type 2 diabetes [63].

POMC (pro-opiomelanocortin) neurons located in the arcuate nucleus (ARC) of the hypothalamus regulate energy homeostasis, secreting α-MSH (α-melanocyte-stimulating hormone) in response to leptin signaling. POMC expression is under epigenetic regulation. The nuclear protein methyl-CpG-binding protein 2 (MeCP2), essential for neuronal function, influences gene expression by interacting with gene promoters. Mice devoid of Mecp2 in Pomc neurons (Mecp2 ^flox/y^/Pomc-Cre (PKO)) are obese and show high levels of plasma leptin. Deletion of Mecp2 increases DNA methylation of the Pomc promoter and reduces Pomc expression. Hypermethylation of the Pomc promoter causes a reduction in transcriptional activity. Mecp2- and cAMP-responsive element binding protein 1 (CREB1) show a functional synergy in regulating the Pomc promoter [64].

**Table 2:** Pharmacological profile and pharmacogenetics of selected epigenetic drugs.

Drug	Properties	Pharmacogenetics
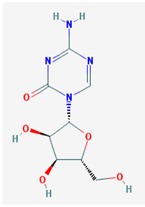	Name: 5-Azacytidine, Azacitidine, Azacytidine, Ladakamycin, Vidaza, Mylosar, Azacitidinum, 5-AZAC IUPAC Name: 4-amino-1-[(2*R*,3*R*,4*S*,5*R*)-3,4-dihydroxy-5-(hydroxymethyl)oxolan-2-yl]-1,3,5-triazin-2-one Molecular Formula: C_8_H_12_N_4_O_5_ Molecular Weight: 244.20468 Category: Pyrimidine nucleoside cytidine analog Mechanism: DNA methyltransferase inhibitor, Telomerase inhibitor Target: DNA (cytosine-5)-methyltransferase 1 (DNMT1) Interactions: Cytidine deaminase Effect: Antineoplastic, Antimetabolite; Methylates CpG residues; Methylates hemimethylated DNA; Mediates transcriptional repression by direct binding to HDAC2	Pathogenic genes: *ALDH3A1*, *CDKN2A*, *MGMT*, *PLA2R1*, *RRM1*, *TNFRSF1B*; Mechanistic genes: *ALDH1A1*, *DAPK1*, *DNMT1*, *DPYD*; *CDKN2A*, *MGMT*, *PLCB1*; Metabolic genes: Substrate: *CDA*, *DCK*, *SLC28A1*, *SLC29A1*, *RRM1*, *RRM2*, *UCK1*, *UCK2*; Inhibitor: *CYP1A2* (weak), *CYP2E1* (weak), *DNMT1*; Inducer: *SULT1C2*; Transporter genes: *SLC5A5*, *SLC28A1*, *SLC29A1* Pleiotropic genes: *BLK*
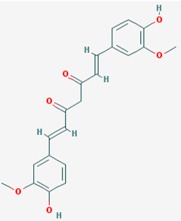	Name: Curcumin, Diferuloylmethane, Natural yellow 3, Turmeric yellow, Turmeric, Kacha haldi, Gelbwurz, Curcuma, Haldar, Souchet; IUPAC Name: (1*E*,6*E*)-1,7-bis(4-hydroxy-3-methoxyphenyl)hepta-1,6-diene-3,5-dione; Molecular Formula: C_21_H_20_O_6_; Molecular Weight: 368.3799; Category: Natural product (*Curcuma longa*); Mechanism: Histone acetyltransferase (HAT) inhibitor; Effect: Non-steroidal anti-inflammatory agent; Antineoplastic; Antioxidant; Cognitive enhancer; Coloring agent; Enzyme inhibitor	Pathogenic genes: *BACE1*, *CCND1*, *CDH1*, *GSK3B*, *IL1A*, *IL6*, *JUN*, *MSR1*, *PSEN1*, *PTGS2*, *SNCA*, *SREBF1*, *TNF*; Mechanistic genes: *AKT1*, *PRKAs*, *BACE1*, *CCND1*, *CDH1*, *CDKs*, *CRM1*, *CTNNB1*, *EGF*, *GSK3B*, *HDACs*; *HIF1A*, *IL1A*, *IL6*, *JUN*, *MMPs*, *MSR1*, *NFKB1*, *NOS2*, *PDGFRs*, *PSEN1*, *PTGS2*, *SNCA*, *SOCS1*, *SOCS3*, *SREBF1*, *STAT3*, *TNF*, *VEGFA*; Metabolic genes: Inhibitor: *CYP2C8*, *CYP2C9*, EP300; Inducer: *CYP2C8*, *CYP2C9*, *CYP2D6*, *CYP3A4*; Transporter genes: *ABCA1*, *SNCA*; Pleiotropic genes: *CTNNB1*, *MSR1*
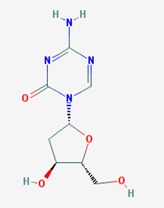	Name: Decitabine, 5-Aza-2′-deoxycytidine, Dacogen, Dezocitidine, 2′-Deoxy-5-azacytidine; IUPAC Name: 4-amino-1-[(2R,4S,5R)-4-hydroxy-5-(hydroxymethyl)oxolan-2-yl]-1,3, 5-triazin-2-one; Molecular Formula: C_8_H_12_N_4_O_4_; Molecular Weight: 228.20528; Category: Nucleoside; Mechanism: DNA methyltransferase inhibitor; Target: DNA (cytosine-5)-methyltransferase 1 (DNMT1); Interactions: Deoxycytidine kinase; Effect: Antineoplastic, Antimetabolite; Enzyme inhibitor; Teratogen	Pathogenic genes: *BRCA1*, *CDKN2B*, *DNMT3A*, *EGFR*, *FOS*, *MGMT*, *MLH1*, *MMP9*, *MYC*, *NOS3*, *NQO1*, *TP53*, *VHL*; Mechanistic genes: *APAF1*, *BRCA1*, *CDKN2B*, *EGFR*, *ICAM1*, *MAGED1*, *MGMT*, *MLH1*, *MMP2*, *MMP9*, *MYC*, *NOS3*, *TIMP3*, *TP53*, *VHL*, *ZNF350*; Metabolic genes: Substrate: *DCK*, *DNMT1*, *CDA*, *SLC29A1*; Inhibitor: *DNMT1*, *DNMT3B*; Inducer: *DPYD*; Transporter genes: *ABCs*, *SLC15s*, *SLC22s*, *SLC28A1*, *SLC29As*; Pleiotropic genes: *HBG1*, *NQO1*, *NTRK2*, *MMP2*, *MSH2*
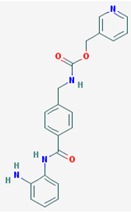	Name: Entinostat, ms-275, 209783-80-2, SNDX-275, MS 275, MS-27-275, SNDX 275, Histone Deacetylase Inhibitor I, S1053_Selleck, MS 27-275; IUPAC Name: pyridin-3-ylmethylN-[[4-[(2-aminophenyl)carbamoyl]phenyl]methyl]carbamate; Molecular Formula: C_21_H_20_N_4_O_3_; Molecular Weight: 376.4085; Category: Benzamide; Mechanism: Class I HDAC inhibitor (HDAC1, 2, 3); Effect: Antineoplastic agent; Histone deacetylase inhibitor; Memory enhancer	Pathogenic genes: *CDH1*; Mechanistic genes: *CDH1*, *HDAC1*, *HDAC2*, *HDAC3*, *KLRK1*; Metabolic genes: Inhibitor: *HDAC1*, *HDAC2*, *HDAC3*; Inducer: *CYP19A1*
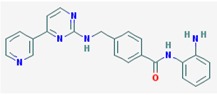	Name: Mocetinostat, MGCD0103, 726169-73-9, MGCD-0103, MGCD 0103, N-(2-Aminophenyl)-4-([[4-(pyridin-3-yl)pyrimidin-2 yl]amino]methyl)benzamide IUPAC Name: *N*-(2-Aminophenyl)-4-[[(4-pyridin-3-ylpyrimidin-2-yl)amino]methyl] benzamide; Molecular Formula: C_23_H_20_N_6_O; Molecular Weight: 396.4445; Category: Benzamide; Mechanism: Class I HDAC inhibitor (HDAC1, 2, 3); Class IV HDAC inhibitor (HDAC11); Effect: Antineoplastic agent; Histone deacetylase inhibitor	Pathogenic genes: *CDKN1A*, *CDKN2B*, *TNF*; Mechanistic genes: *CDKN1A*, *CDKN2B*, *HDAC1*, *HDAC2*, *HDAC3*, *HDAC11*, *NFKB2*, *TNF*; Metabolic genes: Inhibitor: *HDAC1*, *HDAC2*, *HDAC3*, *HDAC11*
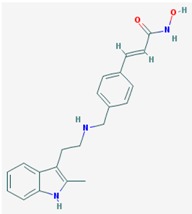	Name: Panobinostat, LBH-589, 404950-80-7, LBH589, Faridak, NVP-LBH589, LBH 589, S1030_Selleck, AC1OCFY8, Panobinostat (LBH589); IUPAC Name: (*E*)-*N*-hydroxy-3-[4-[[2-(2-methyl-1H-indol-3-yl)ethylamino]methyl]phenyl] prop-2-enamide; Molecular Formula: C_21_H_23_N_3_O_2_; Molecular Weight: 349.42622; Category: Hydroxamic acid; Mechanism: Class I HDAC inhibitor (HDAC1, 2, 3, 8); Class IIa HDAC inhibitor (HDAC4, 5, 7, 9); Class IIb HDAC inhibitor (HDAC6, 10); Class IV HDAC inhibitor (HDAC11); Pan-histone deacetylase inhibitor; Effect: Antineoplastic agent; Histone deacetylase inhibitor	Pathogenic genes: *CDKN1A*, *EGFR*, *IL6*, *RASSF1*; Mechanistic genes: *AKT1*, *CDKN1A*, *DAPK1*, *DNMT1*, *EGFR*, *HDACs*, *HIST3H3*, *HIST4H4*, *HSP90As*, *IL6*, *IL10*, *IL12*, *IL23A*, *NFKB2*, *RASSF1*, *TLR3*; Metabolic genes: Substrate: *CYP2C19*, *CYP2D6*, *CYP3A4*; Inhibitor: *AKT1*, *CYP19A1* (strong), *HDACs*; Pleiotropic genes: *IL10*
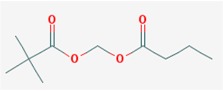	Name: Pivanex, AN-9, Pivalyloxymethyl butyrate, AN 9, 122110-53-6, BRN 4861411, [(2,2-Dimethylpropanoyl)oxy]methyl butanoate; IUPAC Name: Butanoyloxymethyl 2,2-dimethylpropanoate; Molecular Formula: C_10_H_18_O_4_; Molecular Weight: 202.24752; Category: Short-chain fatty acid; Mechanism: Class I HDAC inhibitor (HDAC1, 2, 3, 8); Effect: Antineoplastic agent; Histone deacetylase inhibitor	Pathogenic genes: *BCL2*, *TP53*; Mechanistic genes: *BAX*, *BCL2*, *BCR-ABL*, *HDACs*, *TP53*; Metabolic genes: Inhibitor: *ABCB1*, *HDAC*; Transporter genes: *ABCB1*
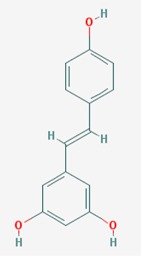	Name: Resveratrol, trans-resveratrol, 501-36-0, 3,4,5-Trihydroxystilbene, 3,4′,5-Stilbenetriol, 3,5,4′-Trihydroxystilbene, Resvida, (*E*)-resveratrol; IUPAC Name: 5-[(*E*)-2-(4-Hydroxyphenyl)ethenyl]benzene-1,3-diol; Molecular Formula: C_14_H_12_O_3_; Molecular Weight: 228.24328; Category: Natural polyphenol; Mechanism: SIRT1 inducer/activator; Effect: Non-steroidal anti-inflammatory agent; Anticarcinogenic; Antimutagenic; Antineoplastic; Antioxidant; Platelet aggregation inhibitor; Enzyme inhibitor; Lifespan extension; Memory improvement; Aβ decrease; Reduction of plaque formation	Pathogenic genes: *BCL2*, *CAV1*, *ESR1*, *ESR2*, *GRIN2B*, *NOS3*, *PTGS2*, *TNFRSF10A*, *TNFRSF10B*; Mechanistic genes: *APP*, *ATF3*, *BAX*, *BAK1*, *BBC3*, *BCL2*, *BCL2L1*, *BCL2L11*, *BIRC5*, *CASP3*, *CAV1*, *CFTR*, *ESR1*, *ESR2*, *GRIN1*, *GRIN2B*, *HTR3A*, *NFKB1*, *NOS3*, *PMAIP1*, *PTGS1*, *PTGS2*, *SIRT1*, *SIRT3*, *SIRT5*, *SRC*, *TNFRSF10A*, *TNFRSF10B*, *TRPs*; Metabolic genes: Substrate: *CYP1A1*, *CYP1A2*, *CYP1B1*, *CYP2E1*, *GSTP1*, *PTGS1*, *PTGS2*; Inhibitor: *CYP1A1*, *CYP1B1*, *CYP2C9*, *CYP2D6*, *CYP3A4*, *NQO2*; Inducer: *CYP1A2*, *SIRT1*; Transporter genes: *ABCC1*, *ABCC2*, *ABCC3*, *ABCC4*, *ABCC8*, *ABCG1*, *ABCG2*, *CFTR*, *TRPs*
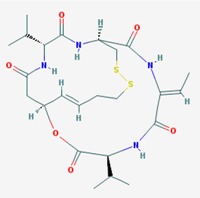	Name: Romidepsin, Depsipeptide, Chromadax, Istodax, Antibiotic FR 901228, FK228, FR 901228, FK-228, NSC 630176, NSC-630176; IUPAC Name: (1*S*,4*S*,7*Z*,10*S*,16*E*,21*R*)-7-ethylidene-4,21-di(propan-2-yl)-2-oxa-12,13-dithia-5,8,20,23-tetrazabicyclo[8.7.6]tricos-16-ene-3,6,9,19,22-pentone; Molecular Formula: C_24_H_36_N_4_O_6_S_2_; Molecular Weight: 540.69584; Category: Cyclic peptide; Mechanism: Class I HDAC inhibitor (HDAC1, 2, 3, 8); Class IIa HDAC inhibitor (HDAC4,5,7,9); Class IIb HDAC inhibitor (HDAC6, 10); Class IV HDAC inhibitor (HDAC11); Effect: Antibiotic; Antineoplastic agent; Histone deacetylase inhibitor	Pathogenic genes: *BCL2*, *CCDN1*, *CDKN1A*, *MYC*, *NF2*, *RB1*, *ROS1*, *TNFSF10*, *VHL*; Mechanistic genes: *BCL2*, *CCDN1*, *CDKN1A*, *FLT1*, *HDAC1*, *HDAC2*, *HDAC3*, *HDAC4*, *HSP90As*, *KDR*, *MYC*, *NF2*, *TNFSF10*, *VEGFs*, *VHL*; Metabolic genes: Substrate: *ABCB1*, *ABCG2*, *CYP1A1* (minor), *CYP2B6* (minor), *CYP2C19* (minor), *CYP3A4* (major), *CYP3A5* (minor), *NR1I3*, *SLCO1B3*; Inhibitor: *ABCB1*, *HDACs*; Inducer: *ABCG2*; Transporter genes: *ABCB1*, *ABCC1*, *ABCG2*, *SLCO1B3* Pleiotropic genes: *CDH1*, *CDKN1A*
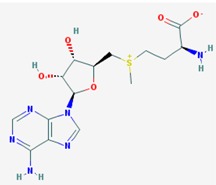	Name: *S*-Adenosylmethionine, Ademetionine, AdoMet, Donamet, S-adenosyl-L-methionine, SAMe, Methioninyladenylate, SAM-e, adenosylmethionine; IUPAC Name: (2*S*)-2-amino-4-[[(2*S*,3*S*,4*R*,5*R*)-5-(6-aminopurin-9-yl)-3,4-dihydroxyoxolan-2-yl]methyl-methylsulfonio]butanoate; Molecular Formula: C_15_H_22_N_6_O_5_S; Molecular Weight: 398.43738; Category: Methyl radical donor; Mechanism: Histone methyltransferase inhibitor; Effect: Antineoplastic; Antiinflammatory; Memory enhancer; PSEN1 repressor	Pathogenic genes: *AKT1*, *ERK*, *GNMT*, *MAT1A*, *PSEN1*; Mechanistic genes: *AMD1*, *CAT*, *CBS*, *GCLC*, *GNMT*, *GSS*, *NOS2*, *ROS1*, *STAT1*, *TNF*; Metabolic genes: Substrate: *COMT*, *GNMT*, *TPMT*, *SRM*; Inhibitor: *ABCB1*, *CYP2E1*, *NOS2*; Transporter genes: *SLC25A26*; Pleiotropic genes: *CAT*, *TNF*
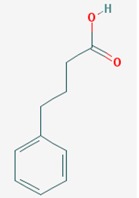	Name: Sodium phenylbutyrate, Buphenyl, 4-Phenylbutiric acid, 4-Phenylbutanoic acid, Benzenebutanoic acid, Benzenebutyric acid, Butyric acid, 4-phenyl-, 1821-12-1, gamma-Phenylbutyric acid; IUPAC Name: 4-Phenylbutanoic acid; Molecular Formula: C_10_H_12_O_2_; Molecular Weight: 164.20108; Category: Short-chain fatty acid; Mechanism: Class I HDAC inhibitor (HDAC1, 2, 3, 8); Class IIa inhibitor (HDAC4,5,7,9); Class IIb inhibitor (HDAC6,10); Effect: Antineoplastic agent; Histone deacetylase inhibitor; Memory improvement; pTau decrease via GSK3β inactivation; C99 and Aβ decrease; Amyloid burden reduction	Pathogenic genes: *ARG1*, *ASS1*, *BCL2*, *CPS1*, *NAGS*, *OTC*; Mechanistic genes: *BCL2*, *BDNF*, *EDN1*, *HDACs*, *HSPA8*, *ICAM1*, *NFKB2*, *NT3*, *VCAM1*; Metabolic genes: Inhibitor: *HDACs*; Inducer: *ARG1*, *CFTR*, *CYP2B6*, *NFKB2*; Transporter genes: *CFTR*; Pleiotropic genes: *ASL*, *BDNF*, *VCAM1*
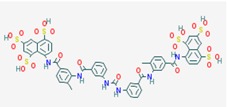	Name: Suramin, Naphuride, Germanin, Naganol, Belganyl, Fourneau, Farma, Antrypol, Suramine, Naganin; IUPAC Name: 8-[[4-methyl-3-[[3-[[3-[[2-methyl-5-[(4,6, 8-trisulfonaphthalen-1-yl)carbamoyl]phenyl]carbamoyl]phenyl] carbamoylamino]benzoyl]amino]benzoyl]amino]naphthalene-1,3,5-trisulfonic acid; Molecular Formula: C_51_H_40_N_6_O_23_S_6_; Molecular Weight: 1297.2797; Category: Polyanionic compound; Mechanism: Class III HDAC/Sirtuin inhibitor (SIRT1-3); Effect: Antineoplastic Agent; Trypanocidal Agent; Antiparasitic; Antinematodal (African trypanosomiasis, Onchocerca); Sirtuin inhibitor	Mechanistic genes: *FSHR*, *IL10*, *P2RY2*, *PDGFRB*, *RYR1*, *SIRT1*, *SIRT2*, *SIRT3*, *SIRT5*; Metabolic genes: Inhibitor: *SIRT1*, *SIRT2*, *SIRT3*
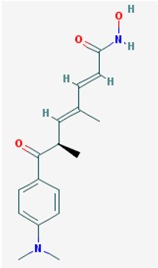	Name: Trichostatin A, 58880-19-6, TSA, Trichostatin A (TSA), CHEBI:46024, TSA; 2,4-Heptadienamide, 7-(4-(dimethylamino)phenyl)-*N*-hydroxy-4,6-dimethyl-7-oxo-7-(4-(Dimethylamino)phenyl)-*N*-hydroxy-4,6-dimethyl-7-oxo-2,4-heptadienamide; [*R*-(*E*,*E*)]-7-[4-(Dimethylamino)phenyl]-*N*-hydroxy-4,6-dimethyl-7-oxo-2,4-heptadienamide; IUPAC Name: (2*E*,4*E*,6*R*)-7-[4-(dimethylamino)phenyl]-*N*-hydroxy-4,6-dimethyl-7-oxohepta-2,4-dienamide; Molecular Formula: C_17_H_22_N_2_O_3_; Molecular Weight: 302.36818; Category: Hydroxamic acid; Mechanism: Class I HDAC inhibitor (HDAC1, 2, 3); Class IIa HDAC inhibitor (HDAC4, 7, 9); Class IIb inhibitor (HDAC6); Effect: Antifungal agent; Antibacterial agent; Histone deacetylase inhibitor; Protein synthesis inhibitor; Antineoplastic; Memory improvement; Rescue of CA3-CA1 LTP in APP/PS1 transgenic models	Pathogenic genes: *BCL2*; Mechanistic genes: *BCL2*, *HDACs*, *IL8*, *IL12A*, *IL12B*, *NFKB2*, *RARB*; Metabolic genes: Substrate: *CYP3A4* (mayor); Inhibitor: *HDACs*; Inducer: *CYP1A1*, *CYP1B1*, *CYP2B6*, *CYP2E1*, *CYP7A1*, *SLC19A3*; Transporter genes: *SLC19A3*
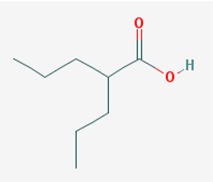	Name: Valproic Acid, 2-Propylpentanoic acid, Depakene, Depakine, Ergenyl, Dipropylacetic acid, Mylproin, Convulex, Myproic Acid; IUPAC Name: 2-Propylpentanoic acid; Molecular Formula: C_8_H_16_O_2_; Molecular Weight: 144.21144; Category: Short-chain fatty acid; Mechanism: Class I HDAC inhibitor (HDAC1, 2, 3, 8); Effect: Anticonvulsant; Mood stabilizer; Antimanic agent; Enzyme inhibitor; Histone deacetylase inhibitor; GABA modulator; Memory improvement; Aβ and pTau decrease; CDK5 inactivation	Pathogenic genes: *CREB1*, *IL6*, *LEP*, *SCN2A*, *TGFB1*, *TNF*, *TRNK*; Mechanistic genes: *ABAT*, *CDK5*, *GSK3B*, *HDAC1*, *HDAC2*, *HDAC3*, *HDAC8*, *HDAC9*, *LEP*, *LEPR*, *SCNs*, *SMN2*; Metabolic genes: Substrate: *ABCB1*, *CYP1A1* (minor), *CYP2A6* (major), *CYP2B6* (minor), *CYP2C9* (major), *CYP2C19* (minor), *CYP2E1* (minor), *CYP3A4* (minor), *CYP4B1* (major), *CYP4F2* (minor), *UGT1A4*, *UGT1A6*, *UGT1A8*, *UGT1A9*, *UGT1A10*, *UGT2B7*; Inhibitor: *ABCB1*, *ACADSB*, *AKR1A1*, *CYP2A6* (moderate), *CYP2C9* (strong), *CYP2C19* (moderate), *CYP2D6* (weak), *CYP3A4* (moderate), *HDAC1*, *HDAC2*, *HDAC3*, *HDAC8*, *HDAC9*, *UGT1A9*, *UGT2B1*, *UGT2B7*; Inducer: *ABCB1*, *AKR1C4*, *CASR*, *CYP2A6*, *CYP2B6*, *CYP3A4*, *CYP7A1*, *MAOA*, *NR1I2*, *SLC5A5*, *SLC6A2*, *SLC12A3*, *SLC22A16;* Transporter genes: *ABCB1*, *ABCC2*, *ABCG1*, *ABCG2*, *SCNs*, *SLC5A5*, *SLC6A2*, *SLC12A3*, *SLC22A16;* Pleiotropic genes: *ABL2*, *AGPAT2*, *ASL*, *ASS1*, *CDK4*, *CHRNA1*, *COL1A1*, *CPS1*, *CPT1A*, *DRD4*, *FMR1*, *FOS*, *HBB*, *HFE*, *HLA-A*, *HLA-B*, *ICAM1*, *IFNG*, *IL6*, *IL10*, *LEPR*, *NAGS*, *NR3C1*, *OTC*, *PTGES*, *STAT3*, *TGFB1*, *TNF*, *TP53*
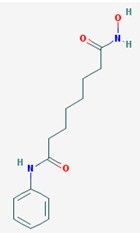	Name: Vorinostat, Suberoylanilide hydroxamic acid (SAHA), Zolinza, Suberanilohydroxamic acid, 149647-78-9,*N*-hydroxy-*N*’-phenyloctanediamide, SAHA cpd; IUPAC Name: *N*’-Hydroxy-*N*-phenyloctanediamide; Molecular Formula: C_14_H_20_N_2_O_3_; Molecular Weight: 264.3202; Category: Hydroxamic acid; Mechanism: Class I HDAC inhibitor (HDAC1, 2, 3, 8); Class IIb inhibitor (HDAC6); Effect: Antineoplastic, Memory improvement	Pathogenic genes: BIRC3, CCND1, *CDKN1A*, CFLAR, CYP19A1, ERBB2, ERBB3, EGFR, RB1, TP53, TNF; Mechanistic genes: *CDK* N1A, EGFR, ERBB2, ERBB3, STATs, TYMS, VEGFs; Metabolic genes: Substrate: *CYP2A6* (minor), *CYP2C9* (minor), *CYP2C19* (major), *CYP2D6* (minor), *CYP3A4* (major); Inhibitor: *HDAC1*, *HDAC2*, *HDAC3*, *HDAC6*; Inducer: *CYP1A1*, *CYP1A2*, *CYP1B1*; Pleiotropic genes: *ALPs*, *TNF*, *TYMS*

*ABAT*: 4-aminobutyrate aminotransferase; *ABCA1*: ATP-binding cassette, sub-family A (ABC1), member 1; *ABCB1*: ATP-binding cassette, sub-family B (MDR/TAP), member 1; *ABCC1*: ATP-binding cassette, sub-family C (CFTR/MRP), member 1; *ABCC2*: ATP-binding cassette, sub-family C (CFTR/MRP), member 2; *ABCC3*: ATP-binding cassette, sub-family C (CFTR/MRP), member 3; *ABCC4*: ATP-binding cassette, sub-family C (CFTR/MRP), member 4; *ABCC8*: ATP-binding cassette, sub-family C (CFTR/MRP), member 8; *ABCG1*: ATP-binding cassette, sub-family G (WHITE), member 1; *ABCG2*: ATP-binding cassette, sub-family G (WHITE), member 2 (Junior blood group); *ABCs*: ATP-binding cassette family; *ABL2*: ABL proto-oncogene 2, non-receptor tyrosine kinase; *ACADSB*: acyl-CoA dehydrogenase, short/branched chain; *AGPAT2*: 1-acylglycerol-3-phosphate O-acyltransferase 2; *AKR1A1*: aldo-keto reductase family 1, member A1 (aldehyde reductase); *AKR1C4*: aldo-keto reductase family 1, member C4; *AKT1*: v-akt murine thymoma viral oncogene homolog 1; *ALDH1A1*: aldehyde dehydrogenase 1 family, member A1; *ALDH3A1*: aldehyde dehydrogenase 3 family, member A1; *ALPs*: alkaline phosphatases; *AMD1*: adenosylmethionine decarboxylase 1; *APAF1*: apoptotic peptidase activating factor 1; *APP*: amyloid β (A4) precursor protein; *ARG1*: arginase 1; *ASL*: argininosuccinate lyase; *ASS1*: argininosuccinate synthase 1; *ATF3*: activating transcription factor 3; *BACE1*: β-site APP-cleaving enzyme 1; *BAK1*: BCL2-antagonist/killer 1; *BAX*: BCL2-associated X protein; *BBC3*: BCL2 binding component 3; *BCL2*: B-cell CLL/lymphoma 2; *BCL2L1*: BCL2-like 1; *BCL2L11*: BCL2-like 11 (apoptosis facilitator); *BCR-ABL*: *BCR*-*ABL* tyrosine kinase fusion; *BDNF*: brain-derived neurotrophic factor; *BIRC3*: baculoviral IAP repeat containing 3; *BIRC5*: baculoviral IAP repeat containing 5; *BLK*: BLK proto-oncogene, Src family tyrosine kinase; *BRCA1*: breast cancer 1, early onset; *CASP3*: caspase 3, apoptosis-related cysteine peptidase; *CASR*: calcium-sensing receptor; *CAT*: catalase; *CAV1*: caveolin 1, caveolae protein, 22kDa; *CBS*: cystathionine-β-synthase; *CCDN1*: cyclin D1; *CDA*: cytidine deaminase; *CDH1*: cadherin 1, type 1; *CDK4*: cyclin-dependent kinase 4; *CDK5*: cyclin-dependent kinase 5; *CDKN1A*: cyclin-dependent kinase inhibitor 1A (p21, Cip1); *CDKN2A*: cyclin-dependent kinase inhibitor 2A; *CDKN2B*: cyclin-dependent kinase inhibitor 2B (p15, inhibits CDK4); *CDKs*: cyclin-dependent kinases; *CFLAR*: CASP8 and FADD-like apoptosis regulator; *CFTR*: cystic fibrosis transmembrane conductance regulator (ATP-binding cassette sub-family C, member 7); *CHRNA1*: cholinergic receptor, nicotinic, alpha 1 (muscle); *COL1A1*: collagen, type I, alpha 1; *COMT*: catechol-O-methyltransferase; *CPS1*: carbamoyl-phosphate synthase 1, mitochondrial; *CPT1A*: carnitine palmitoyltransferase 1A (liver); *CREB1*: cAMP responsive element binding protein 1; *CTNNB1*: catenin (cadherin-associated protein), β 1, 88kDa; *CYP19A1*: cytochrome P450, family 19, subfamily A, polypeptide 1; *CYP1A1*: cytochrome P450, family 1, subfamily A, polypeptide 1; *CYP1A2*: cytochrome P450, family 1, subfamily A, polypeptide 2; *CYP1B1*: cytochrome P450, family 1, subfamily B, polypeptide 1; *CYP2A6*: cytochrome P450, family 2, subfamily A, polypeptide 6; *CYP2C19*: cytochrome P450, family 2, subfamily C, polypeptide 19; *CYP2C8*: cytochrome P450, family 2, subfamily C, polypeptide 8; *CYP2C9*: cytochrome P450, family 2, subfamily C, polypeptide 9; *CYP2D6*: cytochrome P450, family 2, subfamily D, polypeptide 6; *CYP2E1*: cytochrome P450, family 2, subfamily E, polypeptide 1; *CYP3A4*: cytochrome P450, family 3, subfamily A, polypeptide 4; *CYP3A5*: cytochrome P450, family 3, subfamily A, polypeptide 5; *CYP4B1*: cytochrome P450, family 4, subfamily B, polypeptide 1; *CYP4F2*: cytochrome P450, family 4, subfamily F, polypeptide 2; *CYP7A1*: cytochrome P450, family 7, subfamily A, polypeptide 1; *DAPK1*: death-associated protein kinase 1; *DCK*: deoxycytidine kinase; *DNMT1*: DNA (cytosine-5-)-methyltransferase 1; *DNMT3A*: DNA (cytosine-5-)-methyltransferase 3 alpha; *DNMT3B*: DNA (cytosine-5-)-methyltransferase 3 β; *DPYD*: dihydropyrimidine dehydrogenase; *DRD4*: dopamine receptor D4; *EDN1*: endothelin 1; *EGF*: epidermal growth factor; *EGFR*: epidermal growth factor receptor; *EP300*: E1A binding protein p300; *ERBB2*: erb-b2 receptor tyrosine kinase 2; *ERBB3*: erb-b2 receptor tyrosine kinase 3; *ERK*: elk-related tyrosine kinase; *ESR1*: estrogen receptor 1; *ESR2*: estrogen receptor 2 (ER β); *FLT1*: fms-related tyrosine kinase 1; *FMR1*: fragile X mental retardation 1; *FOS*: FBJ osteosarcoma oncogene; *FSHR*: follicle stimulating hormone receptor; *GCLC*: glutamate-cysteine ligase, catalytic subunit; *GNMT*: glycine N-methyltransferase; *GRIN1*: glutamate receptor, ionotropic, N-methyl D-aspartate 1; *GRIN2B*: glutamate receptor, ionotropic, N-methyl D-aspartate 2B; *GSK3B*: glycogen synthase kinase 3 β; *GSS*: glutathione synthetase; *GSTP1*: glutathione S-transferase pi 1; *HBB*: hemoglobin, β; *HBG1*: hemoglobin, gamma A; *HDAC1*: histone deacetylase 1; *HDAC11*: histone deacetylase 11; *HDAC2*: histone deacetylase 2; *HDAC3*: histone deacetylase 3; *HDAC4*: histone deacetylase 4; *HDAC6*: histone deacetylase 6; *HDAC8*: histone deacetylase 8; *HDAC9*: histone deacetylase 9; *HDACs*: histone deacetylases; *HFE*: hemochromatosis; *HIF1A*: hypoxia inducible factor 1, alpha subunit (basic helix-loop-helix transcription factor); *HIST3H3*: histone cluster 3, H3; *HIST4H4*: histone cluster 4, H4; *HLA-A*: major histocompatibility complex, class I, A; *HLA-B*: major histocompatibility complex, class I, B; *HSP90As*: heat shock protein 90kDa alpha (cytosolic), class A; *HSPA8*: heat shock 70kDa protein 8; *HTR3A*: 5-hydroxytryptamine (serotonin) receptor 3A, ionotropic; *ICAM1*: intercellular adhesion molecule 1; *IFNG*: interferon, gamma; *IL10*: interleukin 10; *IL12*: interleukin 12; *IL12A*: interleukin 12A; *IL12B*: interleukin 12B; *IL1A*: interleukin 1, alpha; *IL23A*: interleukin 23, alpha subunit p19; *IL6*: interleukin 6; *IL8*: interleukin 8; IUPAC: International Union of Pure and Applied Chemistry; *JUN*: jun proto-oncogene; *KDR*: kinase insert domain receptor; *KLRK1*: killer cell lectin-like receptor subfamily K, member 1; *LEP*: leptin; *LEPR*: leptin receptor; *MAGED1*: melanoma antigen family D1; *MAOA*: monoamine oxidase A; *MAT1A*: methionine adenosyltransferase I, alpha; *MGMT*: O-6-methylguanine-DNA methyltransferase; *MLH1*: mutL homolog 1; *MMP2*: matrix metallopeptidase 2; *MMP9*: matrix metallopeptidase 9; *MMPs*: matrix metallopeptidases; *MSH2*: mutS homolog 2; *MSR1*: macrophage scavenger receptor 1; *MYC*: v-myc avian myelocytomatosis viral oncogene homolog; *NAGS*: *N*-acetylglutamate synthase; *NF2*: neurofibromin 2 (merlin); *NFKB1*: nuclear factor of kappa light polypeptide gene enhancer in B-cells 1; *NFKB2*: nuclear factor of kappa light polypeptide gene enhancer in B-cells 2 (p49/p100); *NOS2*: nitric oxide synthase 2, inducible; *NOS3*: nitric oxide synthase 3 (endothelial cell); *NQO1*: NAD(P)H dehydrogenase, quinone 1; *NQO2*: NAD(P)H dehydrogenase, quinone 2; *NR1I2*: nuclear receptor subfamily 1, group I, member 2; *NR1I3*: nuclear receptor subfamily 1, group I, member 3; *NR3C1*: nuclear receptor subfamily 3, group C, member 1 (glucocorticoid receptor); *NT3*: 3′-nucleotidase; *NTRK2*: neurotrophic tyrosine kinase, receptor, type 2; *OTC*: ornithine carbamoyltransferase; *P2RY2*: purinergic receptor P2Y, G-protein coupled, 2; *PDGFRB*: platelet-derived growth factor receptor, β polypeptide; *PDGFRs*: platelet-derived growth factor receptors; *PLA2R1*: phospholipase A2 receptor 1, 180kDa; *PLCB1*: phospholipase C, β 1 (phosphoinositide-specific); *PMAIP1*: phorbol-12-myristate-13-acetate-induced protein 1; *PRKAs*: protein kinase family, AMP-activated; *PSEN1*: presenilin 1; *PTGES*: prostaglandin E synthase; *PTGS1*: prostaglandin-endoperoxide synthase 1 (prostaglandin G/H synthase and cyclooxygenase); *PTGS2*: prostaglandin-endoperoxide synthase 2 (prostaglandin G/H synthase and cyclooxygenase); *RARB*: retinoic acid receptor, β; *RASSF1*: Ras association (RalGDS/AF-6) domain family member 1; *RB1*: retinoblastoma 1; *RRM1*: ribonucleotide reductase M1; *ROS1*: ROS proto-oncogene 1, receptor tyrosine kinase; *RRM1*: ribonucleotide reductase M1; *RRM2*: ribonucleotide reductase M2; *RYR1*: ryanodine receptor 1 (skeletal); *SCN2A*: sodium channel, voltage gated, type II alpha subunit; *SCNs*: sodium channel family; *SIRT1*: sirtuin 1; *SIRT2*: sirtuin 2; *SIRT3*: sirtuin 3; *SIRT5*: sirtuin 5; *SLC12A3*: solute carrier family 12 (sodium/chloride transporter), member 3; *SLC15s*: solute carrier family 15; *SLC19A3*: solute carrier family 19 (thiamine transporter), member 3; *SLC19A3*: solute carrier family 19 (thiamine transporter), member 3; *SLC22A16*: solute carrier family 22 (organic cation/carnitine transporter), member 16; *SLC22s*: solute carrier family 22; *SLC25A26*: solute carrier family 25 (S-adenosylmethionine carrier), member 26; *SLC28A1*: solute carrier family 28 (concentrative nucleoside transporter), member 1; *SLC29A1*: solute carrier family 29 (equilibrative nucleoside transporter), member 1; *SLC29As*: solute carrier family 29; *SLC5A5*: solute carrier family 5 (sodium/iodide cotransporter), member 5; *SLC6A2*: solute carrier family 6 (neurotransmitter transporter), member 2; *SLCO1B3*: solute carrier organic anion transporter family, member 1B3; *SMN2*: survival of motor neuron 2, centromeric; *SNCA*: synuclein, alpha (non A4 component of amyloid precursor; *SOCS1*: suppressor of cytokine signaling 1; *SOCS3*: suppressor of cytokine signaling 3; *SRC*: SRC proto-oncogene, non-receptor tyrosine kinase; *SREBF1*: sterol regulatory element binding transcription factor 1; *SRM*: spermidine synthase; *STAT1*: signal transducer and activator of transcription 1, 91kDa; *STAT3*: signal transducer and activator of transcription 3 (acute-phase response factor); *STATs*: signal transducer and activator of transcription family; *SULT1C2*: sulfotransferase family, cytosolic, 1C, member 2; *TGFB1*: transforming growth factor, β 1; *TIMP3*: TIMP metallopeptidase inhibitor 3; *TLR3*: toll-like receptor 3; *TNF*: tumor necrosis factor; *TNFRSF10A*: tumor necrosis factor receptor superfamily, member 10a; *TNFRSF10B*: tumor necrosis factor receptor superfamily, member 10b; *TNFRSF1B*: tumor necrosis factor receptor superfamily, member 1B; *TNFSF10*: tumor necrosis factor (ligand) superfamily, member 10; *TP53*: tumor protein p53; *TPMT*: thiopurine S-methyltransferase; *TRNK*: mitochondrially encoded tRNA lysine; *TRPs*: transient receptor potential cation channels; *TYMS*: thymidylate synthetase; *UCK1*: uridine-cytidine kinase 1; *UCK2*: uridine-cytidine kinase 2; *UGT1A10*: UDP glucuronosyltransferase 1 family, polypeptide A10; *UGT1A4*: UDP glucuronosyltransferase 1 family, polypeptide A4; *UGT1A6*: UDP glucuronosyltransferase 1 family, polypeptide A6; *UGT1A8*: UDP glucuronosyltransferase 1 family, polypeptide A8; *UGT1A9*: UDP glucuronosyltransferase 1 family, polypeptide A9; *UGT2B1*: UDP glucuronosyltransferase 1 family, polypeptide B1; *UGT2B7*: UDP glucuronosyltransferase 2 family, polypeptide B7; *VCAM1*: vascular cell adhesion molecule 1; *VEGFA*: vascular endothelial growth factor A; *VEGFs*: vascular endothelial growth factor family; *VHL*: von Hippel-Lindau tumor suppressor, E3 ubiquitin protein ligase; *ZNF350*: zinc finger protein 350.

Chemically or nutritionally mediated epigenetic changes might lead to adverse health outcomes [65], and considerable evidence links many neuropsychiatric, neurodevelopmental and neurodegenerative disorders with multiple complex interactions between genetics and nutrition [66]. Epigenetic changes associated with nutrition (nutrition epigenetics) are important in understanding human health. Nutritional supplements regulate epigenetic alterations and may be effective in the maintenance of neuronal functions. Folic acid, a cofactor in one-carbon metabolism, reduces hyperhomocysteinemia. An elevated concentration of homocysteine may induce oxidative stress that epigenetically mediates cerebrovascular remodeling, leading to neurodegeneration [67].

## 5. Brain Disorders

Epigenetic mechanisms are influential in brain development, maturation and aging, puberty-related changes, mental disorders, addictive behaviors, and neurodegeneration [8,10,13,14,67,68,69,70,71,72,73]. Prototypal examples of neurodegeneration in which genomic and epigenomic alterations coexist are Huntington’s disease and Alzheimer’s disease. Huntington’s chorea-related striatal degeneration is characterized by: (i) mutations (CAG expansions) in the huntingtin (*HTT*) gene; (ii) mutant HTT-related excitotoxicity, mitochondrial dysfunction, axonal transport deficit, altered proteasome activity, and gene dysregulation; (iii) dysregulation of multiple genes; (iv) interference of nuclear localization of expanded HTT with transcription factors, co-activators, and proteins of the transcriptional machinery; (v) alteration of cytoplasmic retention of the transcriptional repressor REST, which is normally associated with wild-type HTT; (vi) alteration of the transcription of multiple genes involved in neuronal survival, plasticity, signaling, and mitochondrial biogenesis and respiration; (vii) dysmorphic chromatin structure through altered post-translational modifications of histones and methylation of DNA; (viii) multiple alterations of histone post-translational modifications, including acetylation, methylation, ubiquitylation, polyamination, and phosphorylation; (ix) altered expression and regulation of non-coding miRNAs controlled by REST; and (x) concomitant de-repression of downstream mRNA targets [74,75].

Fragile X syndrome (FXS) is a monogenic form of neurodevelopmental cognitive impairment associated with CGG repeat expansions (dynamic mutations) in the 5′UTR of the *FMR1* gene which can be inactivated by epigenetic modifications. An intact *FMR1* coding sequence allows pharmacological reactivation of gene transcription with DNA demethylating agents (5′-aza-2′-deoxycytydine) and/or inhibitors of histone deacetylases (Table 1 and Table 2). DNA methylation is dominant over histone acetylation in silencing the *FMR1* gene. DNA methylation represses FMR1 transcription as confirmed by the existence of rare unaffected males carrying unmethylated full mutations [76].

There are a number of neurodevelopmental disorders in which epigenetic dysregulation plays an important role (autism spectrum disorders, Rett syndrome, fragile X syndrome, Prader–Willi syndrome, Angelman syndrome, and Kabuki syndrome) [77]. Rett syndrome (RTT) is an X-linked neurodevelopmental disease caused by *MECP2* mutations. The MeCP2 protein acts as a transcription repressor by binding to methylated CpG dinucleotides, and also as a transcription activator. MeCP2 is expressed in neurons and in glial cells. Reintroduction of MeCP2 into behaviorally affected Mecp2-null mice after birth rescues neurological symptoms, indicating that epigenetic failures in RTT are reversible [78].

Epigenetic dysregulation is involved in the pathogenesis of autism [79,80]. Mbadiwe and Millis [79] reviewed mechanisms for altering DNA-histone interactions of cell chromatin to regulate gene expression as epigenetic targets for therapeutic interventions. The proposed rationale includes the following sequence: (i) DNA methyltransferases (DNMTs) phosphorylate histone H3 at T6; (ii) the DNMT lysine-specific demethylase-1 prevents demethylation of H3 at K4; (iii) epigenetic changes induce androgen receptor (AR)-dependent gene overactivation, which may explain, in part, the male predominance of autism; (iv) AR-dependent gene overactivation, in conjunction with a DNMT-related oxytocin receptor methylation, may send high arousal inputs to the amygdala, resulting in aberrant socialization, a prime characteristic of autism; (v) dysregulation of histone methyltransferases and histone deacetylases (HDACs) associated with low activity of methyl-CpG-binding protein-2 at cytosine-guanine sites diminishes the capacity for condensing chromatin and silencing genes in the frontal cortex, where decreased cortical interconnectivity occurs in patients with autism; and (vi) HDAC1 inhibition overactivates mRNA transcription, a putative mechanism for the increased number of cerebral cortical columns and local frontal cortex hyperactivity [79,81].

Quality of maternal care experienced during infancy is a key factor that can confer vulnerability or resilience to psychiatric disorders later in life. Experiences within an adverse caregiving environment produce aberrant DNA methylation patterns at various gene loci in the medial prefrontal cortex of developing and adult experimental animals [82].

Altered DNA methylation at the aryl hydrocarbon receptor repressor (AHRR) correlates with self-reported smoking. Smoking was associated with DNA demethylation at two distinct loci within *AHRR* (cg05575921 and cg21161138), and methylation status at the AHRR residue interrogated by cg05575921 was highly correlated with serum cotinine levels [83].

Mutations in isocitrate dehydrogenase 1 and 2 (*IDH1/2*) are present in gliomas and in glioblastoma multiforme (GBM). IDH enzymes catalyze the decarboxylation of isocitrate to generate α-ketoglutarate (αKG). Recurrent mutations at Arg^132^ of IDH1 and Arg^172^ of IDH2 confer a neomorphic enzyme activity that catalyzes reduction of αKG into the putative oncometabolite D-2-hydroxyglutate (D2HG). D2HG inhibits αKG-dependent dioxygenases and creates a cellular state permissive to malignant transformation by altering cellular epigenetics and blocking normal differentiation processes [84]. The Polycomb group (PcGhmg) proteins involved in histone-mediated epigenetics are implicated in the malignant evolution of GBM. Aberrant expression of PcG members has been identified in GBM. *EZH2*, *PHF19*, *CBX8* and *PHC2* were found to be upregulated whereas *CBX7*, *CBX6*, *EZH1* and *RYBP* appeared to be dowregulated. Changes in EZH2, PHF19, CBX7, CBX6 and EZH1 were progressively seen in parallel to increased astrocytoma grade [85].

The type III histone deacetylase sirtuin 1 (Sirt1) is a critical immune regulator by suppressing T cell immunity and macrophage activation in inflammation. Mice with genetic Sirt1 deletion specifically in dendritic cells (DCs) are resistant to myelin oligodendrocyte glycoprotein (MOG)-induced experimental autoimmune encephalomyelitis (EAE). Production of IL-27 and interferon beta 1(IFN-β) increases in DCs by loss of Sirt1 functions. Co-cultivation of Sirt1-null DCs with CD4^+^ T cells inhibits Th17 differentiation, which can be reversed by anti-IL27 and anti-IFN-β antibodies. Acetylation of IRF1, a transcription factor that drives IL-27 production, is antagonized by Sirt1. *IRF1* deletion in Sirt1-null DCs abolishes IL-27 production and suppresses Th17 differentiation. Sirt1 affects DC programs to regulate Th17 differentiation during inflammation [86].

Epigenetic changes also occur after nerve tissue injury. Epigenetic regulation of CC-chemokine ligand (CCL) 2 and CCL3 participates in peripheral sensitization, leading to neuropathic pain. Kiguchi *et al.* [87], in a mouse model of neuropathic pain with partial sciatic nerve (SCN) ligation (PSL), found that mRNA levels of CCL2, CCL3 and their receptors (CCR2 and CCR1/CCR5) were increased in the injured SCN. Lysine 9–acetylated histone H3 (H3K9ac) and lysine 4–trimethylated H3 (H3K4me3) levels were increased in the promoter regions of the *CCL2* and *CCL3* genes in the injured SCN after PSL, reflecting an augmentation of gene expression. Upregulation of CCLs and CCRs was suppressed by anacardic acid, a histone acetyltransferase inhibitor. After nerve injury, these chemokine cascades may elicit chronic neuroinflammation.

DNA methylation is a hallmark of genomic imprinting. Differentially methylated regions (DMRs) are found near and in imprinted genes. Imprinted genes are expressed only from the maternal or paternal allele and their normal balance can be disrupted by uniparental disomy (UPD). A growing number of congenital disorders have been linked to genomic imprinting. Each of these is caused by perturbed gene expression at one principal imprinted domain. Some imprinting disorders (Prader–Willi and Angelman syndromes) are caused by genetic mutations. In other cases (Beckwith–Wiedemann syndrome, Silver–Russell syndrome, transient neonatal diabetes mellitus), imprinted expression is perturbed mostly by epigenetic alterations at imprinting control regions. In a few cases, DNA methylation is altered at multiple imprinted loci, reflecting alterations in common trans-acting factors [88].

Maternal UPD for chromosome 7 (matUPD7) results in Silver-Russell syndrome (SRS) with typical features and growth retardation, but no gene has been conclusively implicated in SRS. Genome-scale analysis of eight matUPD7 patients, a segmental matUPD7q31-qter, a rare patUPD7 case and 10 controls on the Infinium Human Methylation 450K BeadChip with 30,017 CpG methylation probes for chromosome 7 showed highly significant clustering of DMRs only on chromosome 7, including the known imprinted loci growth factor receptor bound protein 10 (*GRB10*), sarcoglycan epsilon/paternally expressed 10 (*SGCE/PEG10*), and mesoderm specific transcript (*PEG/MEST*). Ten novel DMRs on chromosome 7, two DMRs for the predicted imprinted genes *HOXA4* and *GLI3* and one for the disputed imprinted gene *PON1*, and differential expression for three genes with novel DMRs, *HOXA4*, *GLI3*, and *SVOP*, were also demonstrated. Allele-specific expression analysis confirmed maternal-only expression of SVOP-like (*SVOPL*), and imprinting of HOXA4 was supported by monoallelic expression. These results reported by Hannula-Jouppi *et al.* [89] represent the first comprehensive map of parent-of-origin-specific DMRs on human chromosome 7, suggesting many new imprinted sites.

## 6. Alzheimer’s Disease

Alzheimer’s disease (AD) is a complex polygenic/multifactorial disorder, in which hundreds of polymorphic variants of over 600 genes of risk might be involved [13,90,91,92,93]; however, conventional genomics does not explain, in full, AD pathogenesis, in which epigenetics may help to understand some enigmatic events. Major epigenetic mechanisms may contribute to AD pathology, although evidence is still very limited [8,9,10,14,94,95,96]. Many AD-related genes contain methylated CpG sites in their promoter regions, and a genome-wide decrease in DNA methylation has been reported in AD [14,95]. Methylation status of repetitive elements (*i.e.*, Alu, long interspersed nuclear element 1 (LINE-1) and α-satellite (SAT-α)) is a major contributor to global DNA methylation patterns. The study of global DNA methylation levels for long interspersed nuclear element 1 (LINE-1) repetitive sequences in patients with AD and controls did not provide clear results. In one study, no differences in LINE-1 methylation levels were found between patients and controls [97], whereas in another, LINE-1 methylation was found increased in AD patients compared with healthy volunteers [98]. In AD, both hypomethylation and hypermethylation of specific genes have been reported [14]. DNA methylation of the amyloid precursor protein (*APP*) promoter was found to be decreased in the brain of autopsy cases older than 70 years of age as compared with younger cases [99]. The intracellular domain of APP (AICD) has emerged as a key epigenetic regulator of gene expression, controlling a diverse range of genes, including *APP* itself, the amyloid-degrading enzyme neprilysin, and aquaporin-1 [100]. Abnormal processing of neuronal cell membrane APP is accompanied by elevated human serum and cerebrospinal fluid (CSF) levels of 24-hydroxycholesterol, an endogenous ligand of Liver X receptor (LXR-α). There is an epigenomic pathway that connects LXR-α activation with genes involved in the regulation of aberrant Aβ production, leading to the generation of neurotoxic mediators of cell death. LXR-α activation by its specific endogenous or exogenous ligands results in the overexpression of the *PAR-4* gene and the suppression of the *AATF* gene. Overexpression of the *PAR-4* gene is accompanied by aberrant Aβ production followed by reactive oxygen species (ROS) generation and subsequent neuronal death. Aβ-induced heme oxygenase-1 can ensure cholesterol oxidation to provide endogenous ligands for the sustained activation of neuronal LXR-α-dependent epigenomic pathways, leading to neuronal death in AD [101].

Presenilin1 (PSEN1) is modulated by DNA methylation in neuroblastoma cells and Alzheimer’s mice in an experimental model of nutritionally altered one-carbon metabolism. Studies performed on human neuronal cell cultures revealed that deprivation of folate and other B vitamins results in epigenetic modification of *PSEN1* [102].

Several pathogenic genes (*APP*, *PS1*, *APOE*, *BACE*) and many other AD-related susceptibility genes contain methylated CpG sites. The promoter region of the *APP* gene is hypomethylated, with this contibuting to a potential enhancement of Aβ production; however, some authors have reported no relevant changes in *APP* methylation, with an epigenetic drift in AD samples [103]. *BACE* and *PS1* expression is enhanced after folate deprivation–induced hypomethylation, and is restored when folate deficiency is supplemented with SAMe. Aβ may induce genome-wide hypomethylation accompanied by the upregulation of genes involved in neuroinflammation (*TNF*) and apoptosis (caspase-3), which contribute to Aβ production, the process thus entering into a vicious circle [14].

The *APOE* gene exhibits a bimodal structure, with a hypomethylated CpG-poor promoter and a fully methylated 3′-CpG island, containing the sequences for the *APOE4*-haplotype. According to Wang *et al.* [14,103], aberrant epigenetic change in this CpG island may contribute to late-onset AD (LOAD) pathology. A hypermethylated CpG island is present within the *APOE* gene. The *APOE4* sequence may change the epigenetic function of the methylated 3′-CpG island, since the *APOE4* allele induces a C to T transition that is involved in a loss of a methylatable CpG unit [103]. *APOE4* carriers show a dose-dependent risk, and the relative mRNA level of *APOE4* is increased in AD compared to controls, indicating that variability in the neuronal expression of *APOE* contributes to disease risk [104].

Clusterin gene (*CLU*) (apolipoprotein J, ApoJ), together with *APOE*, influences Aβ aggregation and clearance. CLU levels are increased in AD and may be associated with brain atrophy, disease severity, and clinical progression. The promoter region of *CLU* contains a CpG-rich methylation domain. The demethylating effect of 5-aza-2′-deoxycytidine in prostate cancer cell lines increases the expression of CLU [105].

Hyperphosphorylated tau is responsible for the formation of neurofibrillary tangles (NFTs). Changes in methylation status differ among transcription factor binding sites of tau promoter. Binding sites for GCF (granulocyte chemotactic factor), responsible for the repression of GC-rich promoters, were found to be hypomethylated, whereas binding sites for the transcriptional activator SP1 (specificity factor 1) were hypermethylated [106]. High levels of Hcy may induce tau hyperphosphorylation, NFT formation, and senile plaques (SP) formation via inhibition of methyltransferases and hypomethylation of protein phosphatase 2A (PP2A), a dephosphorylating enzyme of phosphorylated tau [107]. In transgenic *APPswe/presenilin (PS) 1* (A246E) mice, PP2A methylation at the L309 site is decreased, in parallel with increased tau phosphorylation at Tau-1 and PHF-1 sites. Aβ_25−35_ induces demethylation and enhances tau phosphorylation [108]. Hypomethylation of PP2A may lead to tau hyperphosphorylation and NFT formation [14].

Sánchez-Mut *et al.* [109] studied 12 distinct mouse brain regions according to their CpG 5′-end gene methylation patterns, and the DNA methylomes obtained from the cerebral cortex were used to identify aberrant DNA methylation changes that occurred in two mouse models of AD. They translated these findings to patients with AD and identified DNA methylation–associated silencing of three target genes: thromboxane A2 receptor (*TBXA2R*), sorbin and SH3 domain containing 3 (*SORBS3*) and spectrin β 4 (*SPTBN4*). These hypermethylation targets suggest that the cyclic AMP response element-binding protein (CREB) activation pathway and the axon initial segment might contribute to AD pathology.

Several components of the cell cycle (P16, P21, P27, P53, RB1, cyclin B2, alternate open reading frame (ARF) protein product) and apoptosis pathways (caspase 1, 3, 7, 8, 9) are regulated by DNA methylation and appear upregulated in AD neurons. SORBS3 (vinexin, SCAM-1 or SH3D4), encoding a cell adhesion molecule expressed in neurons and glia, is progressively hypermethylated with age. S100A2, a member of the S100 family of calcium-binding proteins, which exhibits an age-dependent decrease in DNA methylation later in life, is also hypermethylated in AD [14].

Chaperones participate in AD pathology due to their involvement in protein quality control, folding, and degradation. Silva *et al.* [110] investigated the mRNA and promoter DNA methylation levels of two chaperones, heat shock protein family A8 and 9 (HSPA8 and HSPA9), in postmortem brain tissue (entorhinal and auditory cortices and hippocampus) from healthy elderly and AD subjects as well as in the peripheral blood of healthy elderly and AD patients. No changes were observed in peripheral HSPA8 and HSPA9 expression between elderly controls and AD. A significant downregulation of HSPA8 and HSPA9 was observed in AD across the three brain regions compared to the controls.

In summary, DNA methylation changes are present in AD-related genes; some of these genes are hypermethylated (*MTHFR*, Neprilysin, *MAPT*, *APOE*, *SORB3*), while others have been found to be hypomethylated (*APP*, *BACE*, *PSEN1*, *PP2A*, *S100A2*, *CREB5*) [14,17]. DNA methylation of CpG units by DNA methyltransferases (DNMTs) disrupts the binding of transcription factors and attracts methyl-CpG-binding domain proteins that are associated with gene silencing and chromatin compaction [111]. An association was discovered between the rs1187120 SNP in DNMT3A and annual decline in cognitive functioning, suggesting that DNMT3A moderates cognitive decline in subjects with mild cognitive impairment [112].

A small bulk of recent information [14,21,113] suggests that histone modifications are present in AD: (i) histone acetylation is reduced in AD brain tissues [114] and in AD transgenic models [21]; (ii) levels of HDAC6, a tau-interacting protein and a potential modulator of tau phosphorylation and accumulation, are increased in cortical and hippocampal regions in AD [115]; mice lacking HDAC6 are cognitively normal, but reducing endogenous HDAC6 levels restores learning and memory and α-tubulin acetylation [116]; (iii) SIRT1 is decreased in the parietal cortex of AD patients, and the accumulation of Aβ and tau in AD brains might be related to the loss of SIRT1 [117], since SIRT1 may reduce Aβ production, activating the transcription of ADAM metallopeptidase domain 10 (ADAM10) [118]; (iv) in the brains of twins discordant for AD, trimethylation of H3K9, a marker of gene silencing, and condensation of heterochromatin structure are increased in the temporal cortex and hippocampus of the AD twin as compared to the twin devoid of AD neuropathology [119]; (v) phosphorylation of H3S10, a key regulator in chromatin compaction during cell division, is increased in the cytoplasm of hippocampal neurons in AD cases [120]; (vi) evidence of DNA damage, as reflected by phosphorylated H2AX at Ser139, is present in hippocampal astrocytes of AD patients [121]; (vii) long-term potentiation (LTP) and memory deficits in *APP/PS1* transgenic mice might be mediated in part by decreased H4 acetylation; improving histone acetylation level restores learning after synaptic dysfunction [122]; (viii) acetylation of H3 and H4 is increased in 3xTg-AD neurons relative to non-transgenic neurons [123]; (ix) nuclear translocation of EP300 interacting inhibitor of differentiation 1 (EID1), a CBP/p300 inhibitory protein, is increased in the cortical neurons of AD patients, and overexpression of EID1 is reported to reduce hippocampal LTP and to impair cognitive function via inhibiting CBP/p300 acetyltrasferase activity and disrupting neuronal structure [124]; (x) memory formation leads to a transient increase in acetylation on lysine residues within H2B, H3, H4 [125,126]; (xi) inhibition of HDAC induces dendritic sprouting, increases synaptic number, and improves long-term memory [127]; (xii) overexpression of neuronal HDAC2 decreases dendritic spine density, synapse number, synaptic plasticity and memory formation, and HDAC2 deficiency increases synapse number and memory facilitation [128,129]; (xiii) HDAC4 is involved in learning and synaptic plasticity, and selective inhibition of HDAC4 activity may deteriorate learning and memory [130]; (xiv) treatment of hippocampal neurons with HDAC inhibitors facilitates Bdnf expression via hyperacetylation of histones at the Bdnf promoters [131,132]; (xv) histone (H3K4) methylation participates in the regulation of Bdnf expression and memory formation [133]; (xvi) histone methylation also facilitates memory consolidation coupled with histone acetylation; inhibition of HDACs with sodium butyrate (NaB) causes an increase in H3K4 trimethylation and a decrease in H3K9 dimethylation in the hippocampus after fear conditioning [133]; (xvii) histone H3 acetylation, methylation and phosphorylation are increased in the prefrontal cortex of Tg2576 mice, and histone H4 acetylation is increased in the hippocampal CA1 neurons of these transgenic mice [134].

Several lncRNAs are dysregulated in AD (Sox2OT, 1810014B01Rik, BC200, BACE1-AS, NAT-Rad18, 17A, GDNFOS), Parkinson’s disease (naPINK1, Sox2OT, 1810014B01Rik, BC200), and Huntington’s disease (highly accelerated region 1A (HAR1F), HTTAS, DiGeorge syndrome critical region gene 5 (*DGCR5*), nuclear paraspeckle assembly transcript 1 (NEAT1), taurine up-regulated 1(TUG1)) [26]. miRNAs belong to the class of non-coding regulatory RNA molecules of ~22 nt length and are now recognized to regulate ~60% of all known genes through post-transcriptional gene silencing (RNA interference) (RNAi). Alterations in epigenetically regulated miRNAs may contribute to the abnormal expression of pathogenic genes in AD [25,135]. Examples of miRNAs directly linked to AD pathogenesis include miR-34a (1p36.22), miR-34b/c (11q23.1), miR-107 (10q23.31), miR-124 (8p23.1/8p12.3/20q13.33), miR-125b (11q24.1/21q21.1), and miR-137 (1p21.3); examples of epigenetically regulated miRNAs with targets linked to AD pathogenesis are let-7b (22q13.1), miR-9 (1q22/5q14.3/15q26.1), miR-132/212 (17p13.3), miR-146a (5q34), miR-148a (7p15.2), miR-184 (15q25.1), and miR-200 (miR-200b/200a/429, 1p36.33; miR-200c/141, 12p13.31) [135].

miRNAs can be used as biomarkers to discriminate different disease forms, staging and progression, as well as prognosis [136]. A unique circulating 7-miRNA signature (hsa-let-7d-5p, hsa-let-7g-5p, hsa-miR-15b-5p, hsa-miR-142-3p, hsa-miR-191-5p, hsa-miR-301a-3p and hsa-miR-545-3p) reported by Kumar *et al.* [136] in plasma could distinguish AD patients from normal controls with >95% accuracy. Leidinger *et al.* [137] showed a novel miRNA-based signature for detecting AD from blood samples. Using this 12-miRNA signature, they differentiated between AD and controls with an accuracy of 93%, a specificity of 95% and a sensitivity of 92%. The differentiation of AD from other neurological diseases (mild cognitive impairment (MCI), multiple sclerosis, Parkinson’s disease, major depression, bipolar disorder and schizophrenia) was possible with accuracies of between 74% and 78%. Alexandrov *et al.* [138] found increased levels of miRNA-9, miRNA-125b, miRNA-146a, miRNA-155 in the CSF and brain tissue–derived extracellular fluid from patients with AD, suggesting that these miRNAs might be involved in the modulation or proliferation of miRNA-triggered pathogenic signaling in AD brains.

The interaction of AD-related SNPs with miRNA gene regulation may affect AD susceptibility. Several AD genes interact with miR-214, -23a and& -23b, -486-3p, -30e*, -143, -128, -27a and -27b, -324-5p and -422a, and the dysregulated miRNA network contributes to the aberrant expression of genes in AD [139,140,141].

Several miRNAs have been identified *in vitro* to directly regulate the *APP* mRNA, including miRNA let-7, the miR-20a family (miRs-20a, -17 and -106b), miRs-106a and 520c, miR-101, miR-16, and miRs-147, -153, -323-3p, -644 and -655 [14]. Inhibition of miR-101 overexpression reduces APP and Aβ load in the hippocampal neurons [142]. miR-16 targets APP to potentially modulate AD pathogenesis, and miR-16 overexpression may lead to reduced *APP* expression [143]. Both miR-124 and polypyrimidine tract binding protein 1 (PTBP1) may alter splicing of APP exons 7 and 8 in neuronal cells [144]. miR-124 also regulates the expression of BACE1 [145]. mRNA expression of BACE1 is mediated by both miRNAs (miRs-9, -29a/b-1, -29c, -107, -298, -328 and -485-5p) and long ncRNAs (BACE1-antisense) (BACE1-AS), and is repressed by miRs-29a, -29b-1 and -9 *in vitro*. In transgenic HEK293-APP cells, transient miR-29a/b-1 overexpression decreases BACE1 levels and Aβ production [146]. miR-29c overexpression lowers BACE1 protein levels [147]. miRNAs repress BACE1 through direct binding to sequences in its 3′ untranslated region (3′UTR), whereas miR-485-5p represses BACE1 via binding to its open reading frame in exon 6. miR-107 is downregulated at intermediate stages (Braak stage 3) of AD pathogenesis, and might accelerate AD progression through control of BACE1 [148]. miR-298, miR-328 and miR-195 inversely correlate with BACE1 protein, and downregulate Aβ levels by inhibiting the translation of BACE1 [149,150]. miR-125 decreases whereas BACE1 increases in animal models [150]. Overexpression of miR-485-5p reduces BACE1 protein levels by 30% while knockdown of miR-485-5p increases BACE1 protein levels [151]. BACE1-AS, a ~2 kb conserved ncRNA transcribed from the opposite strand to BACE1 and co-expressed with BACE, is upregulated in AD, potentially promoting Aβ generation and AD pathogenesis. BACE1-AS may enhance *BACE1* mRNA stability by “masking” the binding site for miR-485-5p and prevent miRNA-induced translational repression of *BACE1* mRNA [151,152].

The RNA polymerase III–dependent ncRNA NDM29 promotes APP amyloidogenesis and Aβ secretion [153]. miR-107 levels are reduced in the AD temporal cortex [154,155]. Loss of miRs-9, 29a/b-1, -137 and -181c (currently downregulated in the AD frontal cortex) increases Aβ production and serine palmitoyltransferase (SPT), the first rate-limiting enzyme in ceramide biosynthesis [156]. miRNA-106b (downregulated in the anterior temporal cortex) can influence Aβ metabolism either through direct regulation of APP itself, or via modulating APP trafficking, Aβ clearance and β- and γ-secretase activity through the regulation of the ATP-binding cassette transporter A1 (ABCA1), which is elevated in the hippocampus, correlating with cognitive decline [157]. The brain-expressed ncRNA, 17A, is upregulated in the AD cortex, promoting Aβ in response to neuroinflammation injury [158].

Several miRNAs also regulate tau metabolism. The miR-132/PTBP2 pathway influences microtubule-associated protein tau (MAPT) exon 10 splicing in the brain and may contribute to AD pathogenesis. miR-132 was found to be downregulated in some tauopathies, such as progressive supranuclear palsy (PSP), a major 4R-tau tauopathy, where the protein levels of the neuronal splicing factor PTBP2 were elevated [159]. miR-124, -9, -132 and -137 might regulate the 4R/3R ratio in neuronal cells [159]. Both miR-9 and miR-124 are downregulated in AD, and might affect tau. The miR-15/ERK1 pathway mediates Tau phosphorylation. miR-15a is downregulated in AD brains [160]. The miR-15 family (miR-15a, -16, -195 and -497) targets extracellular signal-regulated kinase 1 (ERK1) expression, and decreased miR-15 levels might participate in neuronal tau hyperphosphorylation. miR-26a represses mRNA of the tau kinase GSK-3β involved in Aβ production and NFT formation [161,162]. miR-26a expression is also altered in AD [163].

In conditional Dicer knockout mice, with reduced brain miRNA production, tau hyperphosphorylation and altered MAPT splicing is observed; reduced miRNA processing in Dicer-1 knockout flies enhances tau-induced neurodegeneration [164].

SIRT1 deacetylates tau, and SIRT1 deficiency increases tau acetylation and the accumulation of hyperphosphorylated tau [117,165]. miR-9, -34c and -181c repress SIRT1 mRNA [166,167]. miR-128 modulates the expression of BCL2 associated athanogene 2 (*BAG2*), the cochaperone involved in tau degradation and aggregation [168]. miR-212 is downregulated in AD, and appears to be involved in NFT density [154,163]. miR-146a is an inflammation effector associated with immune and inflammation signaling by targeting IRAK1. miR-146a upregulation in the AD brain might contribute to neuroinflammation [169,170]. miR-146a interacts with the 3′UTR of Complement factor H (CFH), a repressor of the inflammatory response, which is downregulated in AD [171]. miRNA-146a is an inducible, 22-nucleotide, small RNA overexpressed in the AD brain. Upregulated miRNA-146a targets several inflammation-related and membrane-associated messenger RNAs (mRNAs), including those encoding complement factor-H (CFH) and the interleukin-1 receptor-associated kinase-1 (IRAK-1), resulting in significant decreases in their expression. The most significant miRNA-146a-CFH changes are found in human microglial (HMG) cells, the “resident scavenging macrophages” of the brain [172]. miR-101 interacts with cyclooxygenase-2 (COX-2), and downregulation of miR-101 might induce COX-2 upregulation in AD, enhancing the inflammatory response [142]. miR-124, -125b, -132, -134, -138 and -219 influence synaptic plasticity. miR-132 is dow-regulated and miR-125b is upregulated in different AD brain regions, probably affecting miniature excitatory postsynaptic currents (mEPSCs) [173].

The INK4b-ARF-INK4a locus encodes for two cyclin-dependent kinase inhibitors, p15(INK4b) and p16(INK4a), and a regulator of the p53 pathway, ADP-ribosylation factor (ARF), antisense noncoding RNA in the INK4 locus (ANRIL), a non-coding RNA, is also transcribed from the locus. ARF, p15(INK4b) and p16(INK4a) are well-established tumor suppressors whose function is frequently disabled in human cancers. SNPs mapping in the vicinity of ANRIL are linked to a wide spectrum of conditions, including cardiovascular disease, ischemic stroke, type 2 diabetes, frailty and AD. The INK4b-ARF-INK4a locus is regulated by Polycomb repressive complexes (PRCs), and its expression can be invoked by activating signals. Other epigenetic modifiers such as the histone demethylases JMJD3 and JHDM1B, the SWI/SNF chromatin remodeling complex and DNA methyltransferases regulate the locus interplaying with PRCs [174].

## 7. Pharmacoepigenetics

Pharmacogenomics accounts for 30%–90% variability in pharmacokinetics and pharmacodynamics; however, pharmacogenetics alone does not predict all phenotypic variations in drug response. Individual differences in drug response are associated with genetic and epigenetic variability and disease determinants [175,176]. The genes involved in the pharmacogenomic response to drugs fall into five major categories: (i) genes associated with disease pathogenesis; (ii) genes associated with the mechanism of action of drugs (enzymes, receptors, transmitters, messengers); (iii) genes associated with drug metabolism such as (a) phase I reaction enzymes (alcohol dehydrogenases, aldehyde dehydrogenases, aldo-keto reductases, amine oxidases, carbonyl reductases, cytidine deaminase, cytochrome P450 family, cytochrome b5 reductase, dihydroprimidine dehydrogenase, esterases, epoxidases, flavin-containing monooxygenases, glutathione reductase/peroxidases, short-chain dehydrogenases/reductases, superoxide dismutases, and xanthine dehydrogenase) and (b) phase II reaction enzymes (amino acid transferases, dehydrogenases, esterases, glucuronosyl transferases, glutathione transferases, methyl transferases, *N*-acetyl transferases, thioltransferase, and sulfotransferases (Table 3)); (iv) genes associated with drug transporters such as *ABC* genes, especially *ABCB1* (ATP-binding cassette, subfamily B, member 1; P-glycoprotein-1, P-gp1; Multidrug Resistance 1, MDR1), *ABCC1*, *ABCG2* (White1), genes of the solute carrier superfamily (*SLC*) and solute carrier organic (*SLCO*) transporter family, responsible for the transport of multiple endogenous and exogenous compounds, including folate (*SLC19A1*), urea (*SLC14A1*, *SLC14A2*), monoamines (*SLC29A4*, *SLC22A3*), aminoacids (*SLC1A5*, *SLC3A1*, *SLC7A3*, *SLC7A9*, *SLC38A1*, *SLC38A4*, *SLC38A5*, *SLC38A7*, *SLC43A2*, *SLC45A1*), nucleotides (*SLC29A2*, *SLC29A3*), fatty acids (*SLC27A1-6*), neurotransmitters (*SLC6A2* (noradrenaline transporter), *SLC6A3* (dopamine transporter), *SLC6A4* (serotonin transporter, SERT), *SLC6A5*, *SLC6A6*, *SLC6A9*, *SLC6A11*, *SLC6A12*, *SLC6A14*, *SLC6A15*, *SLC6A16*, *SLC6A17*, *SLC6A18*, *SLC6A19*), glutamate (*SLC1A6*, *SLC1A7*), and others) (Table 3); and (v) pleiotropic genes [13,177,178,179,180,181].

The tissue-specific expression of genes involved in the pharmacogenetic processes is under epigenetic regulation; consequently, epigenetics plays a key role in drug efficacy and safety, and in drug resistance as well. Epigenetic changes affect cytochrome P450 enzyme expression, major transporter function, and nuclear receptor interactions [175,182]. Pioneering pharmacoepigenetic studies illustrate the epigenetic regulation of genes encoding drug-metabolizing enzymes (*CYP1A1*, *1A2*, *1B1*, *1A6*, *2A13*, *2B6*, *2C8*, *2C9*, *2C18*, *2C19*, *2D6*, *2E1*, *2J2*, *2F1*, *2R1*, *2S1*, *2W1*, *3A4*, *3A5*, *3A7*, *3A43*, *UGT1*, *GSTP1*), drug transporters (*ABCB1*/*MDR1*/*P-gp*, *ABCC1*/*MRP1*, *ABCC11*/*MRP8*, *ABCG2*/*BCRP*, *SLC19A1*, *SLC22A8*), and nuclear receptors (*RARB2*, *ESR1*, *NR1I2*, *HNF41*) [175,182,183].

Epigenetic changes in metabolic genes may affect circadian rythms and secretory patterns regulated by hypothalamo-hypophyseal-peripheral pathways. *Cyp51*, *Cyp11a1*, *Cyp17a1*, *Cyb11b1*, *Cyp11b2* and *Cyp21a1* genes are involved in the adrenal production of corticosteroids under circadian variation. cAMP responsive element modulator (CREM) isoforms contribute to the circadian expression of steroidogenic CYPs in the mouse adrenal gland. CREM-dependent hypomethylation of the Cyp17a1 promoter results in higher Cyp17a1 mRNA and protein expression in the knockout adrenal glands, indicating that products of the *Crem* gene control the epigenetic repression of Cyp17 in mouse adrenal glands [184].

DNA methylation and chromatin accessibility of the P1.5/2 promoter region correlate with expression levels of the *Cyp19* gene, which encodes aromatase cytochrome P450 (P450arom; EC 1.14.14.1). The first step of regulation of P450arom expression, and hence enzyme activity and estrogen production, takes place at the level of Cyp19 transcription, which is driven by a proximal promoter region, P1.5/2, in the sheep placenta. Placental estrogens play an important role as local regulators of placental growth and differentiation during gestation, and toward term they are also involved in the preparation of parturition [185].

**Table 3:** Genes involved in drug metabolism and transport.

Phase	Family	Drug Metabolism-Related Genes
Phase I Enzymes	Alcohol Dehydrogenases	*ADH1*–*7*: Alcohol dehydrogenases 1–7
		*ADHFE1*: Alcohol dehydrogenase, iron containing 1
	Aldehyde Dehydrogenases	*ALDH1A1*–*3*: Aldehyde dehydrogenase family 1, members A1, A2 and A3
		*ALDH1B1*: Aldehyde dehydrogenase family 1, member B1
		*ALDH2*: Aldehyde dehydrogenase family 2 (mitochondrial)
		*ALDH3A1*–*2*: Aldehyde dehydrogenase family 3, members A1 and A2
		*ALDH3B1*: Aldehyde dehydrogenase family 3, members B1 and B2
		*ALDH4*–*9*: Aldehyde dehydrogenase families 4–9
		*AOX1*: Aldehyde oxidase 1
	Aldo-keto Reductases	*AKR1A1*–*D1*: Aldo-keto reductase family 1, members A1, B1, C1 and D1
	Amine Oxidases	*MAOA-B*: Monoamine oxidases A and B
		*SMOX*: Spermine oxidase
	Carbonyl Reductases	*CBR1*–*4*: Carbonyl reductases 1–4
	Cytidine Deaminase	*CDA*: Cytidine deaminase
	Cytochrome P450 family	*CYP1*–*51*: Cytochrome P450, families 1–51
		*POR*: P450 (cytochrome) oxidoreductase
		*TBXAS1*: Thromboxane A synthase 1 (platelet)
	Cytochrome b5 Reductase	*CYB5R3*: Cytochrome b5 reductase 3
	Dihydroprimidine Dehydrogenase	*DPYD*: Dihydropyrimidine dehydrogenase
	Esterases	*AADAC*: Arylacetamide deacetylase
		*CEL*: Carboxyl ester lipase
		*CES1*–*5*: Carboxylesterases 1–5
		*ESD*: Esterase D
		*GZMA*: Granzyme A (granzyme 1, cytotoxic T-lymphocyte-associated serine esterase 3)
		*GZMB*: Granzyme B (granzyme 2, cytotoxic T-lymphocyte-associated serine esterase 1)
		*PON1*–*3*: Paraoxonases 1–3
		*UCHL1 and L3*: Ubiquitin carboxyl-terminal esterases L1 and L3 (ubiquitin thiolesterases)
	Epoxidases	*EPHX1*–*2*: Epoxide hydrolases 1 and 2, microsomal (xenobiotic)
	Flavin-containing Monooxygenases	*FMO1*–*6*: Flavin-containing monooxygenases 1–6
	Glutathione Reductase/Peroxidases	*GPX1*–*7*: Glutathione peroxidases 1–7
		*GSR*: Glutathione reductase
	Peptidases	*DPEP1*: Dipeptidase 1 (renal)
		*METAP1*: Methionyl aminopeptidase 1;
	Prostaglandin-endoperoxide Synthases	*PTGS1*–*2*: Prostaglandin-endoperoxide synthases 1 and 2 (prostaglandin G/H synthases and cyclooxygenases)
	Short-chain Dehydrogenases/Reductases	*DHRS1*–*13*: Dehydrogenase/reductase (SDR family) members 1–13
		*DHRSX*: Dehydrogenase/reductase (SDR family) X-linked
		*HSD11B1*: Hydroxysteroid (11-β) dehydrogenase 1
		*HSD17B10*, *11* and *14*: Hydroxysteroid (17-β) dehydrogenases 10, 11 and 14
	Superoxide Dismutase	*SOD1*–*2*: Superoxide dismutases 1 and 2
	Xanthine Dehydrogenase	*XDH*: Xanthine dehydrogenase
Phase II Enzymes	Amino Acid Transferases	*AGXT*: Alanine-glyoxylate aminotransferase
		*BAAT: Bile acid CoA*: amino acid *N*-acyltransferase (glycine N- choloyltransferase)
		*CCBL1*: Cysteine conjugate-β lyase, cytoplasmic
	Dehydrogenases	*NQO1*–*2*: NAD(P)H dehydrogenase, quinones 1 and 2
		*XDH*: Xanthine dehydrogenase
	Esterases	*CES1*–*5*: Carboxylesterases 1–5
	Glucuronosyl Transferases	*DDOST*: Dolichyl-diphosphooligosaccharide—protein glycosyltransferase subunit (non-catalytic)
		*UGT1*–*8*: UDP glucuronosyltransferase families 1–8
	Glutathione Transferases	*GSTA1*–*5*: Glutathione *S*-transferases alpha 1–5
		*GSTK1*: Glutathione *S*-transferase kappa 1
		*GSTM1*–*5*: Glutathione *S*-transferases mu 1–5
		*GSTCD*: Glutathione *S*-transferase, C-terminal domain containing
		*GSTO1*–*2*: Glutathione *S*-transferases omega 1 and 2
		*GSTP1*: Glutathione *S*-transferase pi 1
		*GSTT1*–*2*: Glutathione *S*-transferases theta 1 and 2
		*GSTZ1*: Glutathione *S*-transferase zeta 1
		*MGST1*–*3*: Microsomal glutathione *S*-transferases 1–3
		*PTGES*: Prostaglandin E synthase
	Methyl Transferases	*AS3MT*: Arsenic (+3 oxidation state) methyltransferase
		*ASMT*: Acetylserotonin *O*-methyltransferase
		*COMT*: Catechol-*O*-methyltransferase
		*GAMT*: Guanidinoacetate *N*-methyltransferase
		*GNMT*: Glycine *N*-methyltransferase
		*HNMT*: Histamine *N*-methyltransferase
		*INMT*: Indolethylamine *N*-methyltransferase
		*NNMT*: Nicotinamide *N*-methyltransferase
		*PNMT*: Phenylethanolamine *N*-methyltransferase
		*TPMT*: Thiopurine *S*-methyltransferase
	*N*-Acetyl Transferases	*AANAT*: Aralkylamine *N*-acetyltransferase
		*ACSL1*–*4*: Acyl-CoA synthetase long-chain family, members 1–4
		*ACSM1*–*3*: Acyl-CoA synthetase medium-chain family, member s1–3
		*NAT1*–*2*: *N*-acetyltransferases 1and 2
		*NAA20*: *N*(α)-acetyltransferase 20, NatB catalytic subunit
		*SAT1*: Spermidine/spermine *N*1-acetyltransferase 1
	Thioltransferase	*GLRX*: Glutaredoxin (thioltransferase)
	Sulfotransferases	*CHST1*–*13*: Carbohydrate sulfotransferases 1–13
		*SULT1A*–*3*: Sulfotransferase family, cytosolic, 1A, phenol-preferring, members 1–3,
		*SULT1B*: Sulfotransferase family, cytosolic, 1B, member 1
		*SULT1C1*–*4*: Sulfotransferase family, cytosolic, 1C, members 1–4
		*SULT1E1*: Sulfotransferase family 1E, estrogen-preferring, member 1
		*SULT2A1*: Sulfotransferase family, cytosolic, 2A, dehydroepiandrosterone (DHEA)-preferring, member 1
		*SULT2B1*: Sulfotransferase family, cytosolic, 2B, member 1
		*SULT4A1*: Sulfotransferase family 4A, member 1
		*SULT6B1*: Sulfotransferase family, cytosolic, 6B, member 1
		*TST*: Thiosulfate sulfurtransferase (rhodanese)
		*GAL3ST1*: Galactose-3-*O*-sulfotransferase 1
	**Family**	**Transporter Genes**
	ATP-binding Cassette Transporters	*ABCA1*–*13*: ATP-binding cassette, sub-family A (ABC1), members 1–13
		*ABCB1*–*11*: ATP-binding cassette, sub-family B (MDR/TAP), members 1–11
		*ABCC1*–*13*: ATP-binding cassette, sub-family C (CFTR/MRP), members1–13
		*ABCD1*–*4*: ATP-binding cassette, sub-family D (ALD), members 1–4
		*ABCE1*: ATP-binding cassette, sub-family E (OABP), member 1
		*ABCF1*–*3*: ATP-binding cassette, sub-family F (GCN20), members 1–3
		*ABCG1*–*8*: ATP-binding cassette, sub-family G (WHITE), members 1–8
	ATPases	*ATP1A1*–*4*: ATPase, Na^+^/K^+^ transporting, polypeptides alpha 1–4
		*ATP2A1*–*2*: ATPase, Ca^++^ transporting, cardiac muscle, members 1 and 2
		*ATP2A3*: ATPase, Ca^++^ transporting, ubiquitous
		*ATP2B1* –*4*: ATPase, Ca^++^ transporting, plasma membranes 1–4
		*ATP2C1*–*2*: ATPase, Ca^++^ transporting, type 2C, members 1 and 2
		*ATP4A*–*B*: ATPase, H^+^/K^+^ exchanging, alpha and β polypeptides
		*ATP7A*–*B*: ATPase, Cu^++^ transporting, alpha and β polypeptides
		*ATP8A1*–*2*: ATPase, aminophospholipid transporter (APLT), Class I, type 8A, members 1 and 2
		*ATP8B1*–*4*: ATPase, Class I, type 8B, members 1–4
		*ATP9A*–*B*: ATPase, class II, types 9A and 9B
		*ATP10A*,*B and D*: ATPase, Class V, types 10A, 10B and 10D
		*ATP11A*–*C*: ATPase, Class VI, type 11A, 11B and 11C
		*ATP12A*: ATPase, H^+^/K^+^ transporting, nongastric, α polypeptide
		*ATP13A1*–*5*: ATPase types 13A1–5
		*ATP6N1C*: T-cell, immune regulator 1, ATPase, H^+^ transporting, lysosomal V0 subunit A3
		*ATP6V1A*: ATPase, H^+^ transporting, lysosomal 70 kDa, V1 subunit A
		*ATP6V1B1*–*2*: ATPase, H^+^ transporting, lysosomal 56/58 kDa, V1 subunit B, isoforms 1 and 2
		*ATP6V1C1*–*2*: ATPase, H^+^ transporting, lysosomal 42 kDa, V1 subunits C1 and C2
		*ATP6V1D*: ATPase, H^+^ transporting, lysosomal 34 kDa, V1 subunit D
		*ATP6V1E1*–*2*: ATPase, H^+^ transporting, lysosomal 31 kDa, V1 subunit E isoforms 1 and 2
		*ATP6V1F*: ATPase, H^+^ transporting, lysosomal 14 kDa, V1 subunit F
		*ATP6V1G1*–*3*: ATPase, H^+^ transporting, lysosomal 13 kDa, V1 subunit G isforms 1–3
		*ATP6V1H*: ATPase, H^+^ transporting, lysosomal 50/57 kDa, V1 subunit H
		*ATP6V0A1*, *A2 and A4*: ATPase, H^+^ transporting, lysosomal V0 subunits a1, a2 and a4
		*ATP6V0B*: ATPase, H^+^ transporting, lysosomal 21k Da, V0 subunit b
		*ATP6V0C*: ATPase, H^+^ transporting, lysosomal 16k Da, V0 subunit c
		*ATP6V0D1*–*2*: ATPase, H^+^ transporting, lysosomal 38 k Da, V0 subunits d1 and d2
		*ATP6V0E1*–*2*: ATPase, H^+^ transporting, lysosomal 9k Da, V0 subunits e1 and e2
		*TCIRG1*: T-cell, immune regulator 1, ATPase, H^+^ transporting, lysosomal V0 subunit A3
		*ATP5A*–*E*: ATP synthase, H^+^ transporting, mitochondrial F1 complex, subunits α, β, γ, δ and epsilon
		*ATP5F*–*L*: ATP synthase, H^+^ transporting, mitochondrial F0 complex, subunits B–G
		*ATP5O*: ATP synthase, H^+^ transporting, mitochondrial F1 complex, O subunit
	Solute Carriers	*SLC1A1*–*7*: Solute carrier family 1 (High-affinity glutamate and neutral amino acid transporters), members1-7
		*SLC2A1*–*14*: Solute carrier family 2 (facilitated glucose transporters), members 1–14
		*SLC3A1*–*2*: Solute carrier family 3 (amino acid transporter heavy chains), members 1 and 2
		*SLC4A1*–*9*: Solute carrier family 4 (Bicarbonate transporters), members 1–9
		*SLC5A1*–*12*: Solute carrier family 5 (sodium/glucose cotransporters), members 1–12
		*SLC1A1*–*7*: Solute carrier family 1 (High-affinity glutamate and neutral amino acid transporters), members1-7
		*SLC2A1*–*14*: Solute carrier family 2 (facilitated glucose transporters), members 1–14
		*SLC3A1*–*2*: Solute carrier family 3 (amino acid transporter heavy chains), members 1 and 2
		*SLC4A1*–*9*: Solute carrier family 4 (Bicarbonate transporters), members 1–9
		*SLC5A1*–*12*: Solute carrier family 5 (sodium/glucose cotransporters), members 1–12
		*SLC6A1*–*20*: Solute carrier family 6 (neurotransmitter transporters), members 1–20
		*SLC7A1*–*14*: Solute carrier family 7 (cationic amino acid transporter, y+ system), members 1–14
		*SLC8A1*–*3*: Solute carrier family 8 (sodium/calcium exchangers), members 1–3
		*SLC8B1*: Solute carrier family 8 (sodium/lithium/calcium exchanger), member B1
		*SLC9A1* –*10*: Solute carrier family 9 (sodium/hydrogen exchangers), members 1–10
		*SLC9B1*–*2*: Solute carrier family 9, subfamily B (NHA1, cation proton antiporters), members 1 and 2
		*SLC10A1*–*7*: Solute carrier family 10 (sodium/bile acid cotransporters), members 1–7
		*SLC11A1*–*2*: Solute carrier family 11 (proton-coupled divalent metal ion transporters), members 1 and 2
		*SLC12A1*–*9*: Solute carrier family 12 (electroneutral cation-coupled chloride cotransporters), members 1–9
		*SLC13A1*–*4*: Solute carrier family 13 (Na-coupled di- and tri-carboxylate/sulfate transporters), members 1–4
		*SLC14A1*–*2*: Solute carrier family 14 (urea transporters), members 1 and 2
		*SLC15A1*–*4*: Solute carrier family 15 (oligopeptide transporters), members 1–4
		*SLC16A1*–*14*: Solute carrier family 16 (monocarboxylic acid transporters), members 1–14
		*SLC17A1*–*4*: Solute carrier family 17 (sodium phosphate), members 1–4
		*SLC17A5*: Solute carrier family 17 (anion/sugar transporters), member 5
		*SLC17A6*–*8*: Solute carrier family 17 (sodium-dependent inorganic phosphate cotransporters), members 6–8
		*SLC17A9*: Solute carrier family 17 (vesicular nucleotide transporter), member 9
		*SLC18A1*–*3*: Solute carrier family 18 ( Vesicular amine transporters), members 1–3
		*SLC18B1*: Solute carrier family 18, subfamily B, member 1
		*SLC19A1*–*3*: Solute carrier family 19 (folate/thiamine transporters), members 1–3
		*SLC20A1*–*2*: Solute carrier family 20 (phosphate transporters), members 1 and 2
		*SLCO1A2*: Solute carrier organic anion transporter family, member 1A2
		*SLCO1B1 and 1B3*: Solute carrier organic anion transporter family, members 1B1and 1B3
		*SLCO1C1*: Solute carrier organic anion transporter family, member 1C1
		*SLCO2A1*: Solute carrier organic anion transporter family, member 2A1
		*SLCO2B1*: Solute carrier organic anion transporter family, member 2B1
		*SLCO3A1*: Solute carrier organic anion transporter family, member 3A1
		*SLCO4A1*: Solute carrier organic anion transporter family, member 4A1
		*SLCO4C1*: Solute carrier organic anion transporter family, member 4C1
		*SLCO5A1*: Solute carrier organic anion transporter family, member 5A1
		*SLC22A1*–*25*: Solute carrier family 22 (Organic cation/anion/zwitterion transporters), members 1–25
		*SLC23A1*–*3*: Solute carrier family 23 (nucleobase transporters), members 1–3
		*SLC24A1*–*-5*: Solute carrier family 24 (sodium/potassium/calcium exchangers), members 1–3
		*SLC25A1*–*53*: Solute carrier family ( mitochondrial transporters) 24, members 1–53
		*SLC26A1*–*11*: Solute carrier family 26 (anion exchangers), members 1–11
		*SLC27A1*–*6*: Solute carrier family 27 (fatty acid transporters), members 1–6
		*SLC28A1*–*3*: Solute carrier family 28 (sodium-coupled nucleoside transporters), members 1–3
		*SLC29A1*–*4*: Solute carrier family 29 (nucleoside transporters), members 1–4
		*SLC30A1*–*10*: Solute carrier family 30 (zinc transporters), members 1–10
		*SLC31A1*–*2*: Solute carrier family 31 (copper transporters), members 1 and 2
		*SLC32A1*: Solute carrier family 32 (GABA vesicular transporter), member 1
		*SLC33A1*: Solute carrier family 33 (acetyl-CoA transporter), member 1
		*SLC34A1*–*3*: Solute carrier family 34 (sodium phosphates), members 1–3
		*SLC35A1*–*5*: Solute carrier family 35, members A1–5
		*SLC35B1*–*4*: Solute carrier family 35, members B1–4
		*SLC35C1*–*2*: Solute carrier family 35 (GDP-fucose transporters), members C1 and C2
		*SLC35D1*–*3*: Solute carrier family 35 (UDP-glucuronic acid/UDP-*N*-acetylgalactosamine dual transporters), members D1–3
		*SLC35E1*–*4*: Solute carrier family 35, members E1–4
		*SLC35F1*–*6*: Solute carrier family 35, members F1–6
		*SLC35G1*–*6*: Solute carrier family 35, members G1–6
		*SLC36A1*–*4*: Solute carrier family 36 (proton/amino acid symporters), members 1–4
		*SLC37A1*–*4*: Solute carrier family 37 (sugar-phosphate/phosphate exchangers), members 1–4
		*SLC38A1*–*11*: Solute carrier family 38, member 1
		*SLC39A1*–*14*: Solute carrier family 39 (zinc/metal ion transporters), members 1–14
		*SLC40A1*: Solute carrier family 40 (iron-regulated transporter), member 1
		*SLC41A1*–*3*: Solute carrier family 41, members 1–3
		*RHAG*, *BG and CG*: Rhesus blood group, A, B and C glycoproteins
		*SLC43A1*–*3*: Solute carrier family 43, members 1–3
		*SLC44A1*–*5*: Solute carrier family 44, members 1–5
		*SLC45A1*–*4*: Solute carrier family 45, members 1–4
		*SLC46A1–3*: Solute carrier family 46, members 1–3
		*SLC47A1*–*2*: Solute carrier family 47, members 1 and 2
		*SLC48A1*: Solute carrier family 48 (heme transporter), member 1
		*FLVCR1*–*2*: Feline leukemia virus subgroup C cellular receptor family, members 1and 2
		*SLC50A1*: Solute carrier family 50 (sugar transporter), member 1
		*SLC51A and B*: Solute carrier family 51, subunits alpha and β
		*SLC52A1*–*3*: Solute carrier family 52, (riboflavin transporters), members 1–3
	Miscellanea	*AQP1*: Aquaporin 1 (Colton blood group)
		*AQP7*: Aquaporin 7
		*AQP9*: Aquaporin 9
		*MVP*: Major vault protein
		*MT2A*: Metallothionein 2A
		*MT3*: Metallothionein 3

Park *et al.* [186] studied the epigenetic regulation of *CYP* genes (*CYP1A1*, *CYP1A2*, *CYP1B1*, *CYP2D6*, *CYP2E1*) in human pluripotent stem cell–derived hepatocytes and in primary hepatocytes. Transcript levels of major *CYP* genes were much lower in human embryonic stem cell–derived hepatocytes (hESC-Hep) than in human primary hepatocytes (hPH). CpG islands of *CYP* genes were hypermethylated in hESC-Hep, whereas they had an open chromatin structure, as represented by hypomethylation of CpG sites and permissive histone modifications, in hPH. Inhibition of DNA methyltransferases (DNMTs) during hepatic maturation induced demethylation of the CpG sites of CYP1A1 and CYP1A2, leading to the upregulation of their transcription. Combinatorial inhibition of DNMTs and histone deacetylases (HDACs) increased the transcript levels of CYP1A1, CYP1A2, CYP1B1, and CYP2D6. According to these data, it is likely that the limited expression of *CYP* genes in hESC-Hep is modulated by epigenetic regulatory factors such as DNMTs and HDACs.

Exposure to toxicants and pollutants, such as tobacco and alcohol, potentially associated with head and neck squamous cell carcinoma (HNSCC), may alter the expression of metabolic genes contributing to toxicity and disease. For instance, the study of genome-wide RNA-seq of tongue samples of the combined 4-nitroquinoline-1-oxide (4-NQO) oral carcinogenesis and Meadows-Cook alcohol mouse models revealed changes in transcripts that mediate alcohol metabolism and oxidative stress (Aldh2, Aldh1a3, Adh1, Adh7, and Cyp2a5) in mice treated with 4-NQO followed by ethanol (4-NQO/EtOH). Global increases in specific histone acetylation and methylation epigenetic marks (H3K27ac, H3K9/14ac, H3K27me3, and H3K9me3) were found in the oral cavities. The Aldh2 promoter showed increased H3K27me3 marks, and Aldh2 mRNA levels were reduced 10-fold in 4NQO/EtOH samples [187].

Phthalates are the largest group of environmental pollutants and are considered toxicants to the endocrine system. Sekaran and Jadadeesan [188] studied the effect of *in utero* exposure of di(2-ethylhexyl)phthalate (DEHP) on Leydig cell steroidogenesis in F1 male offspring, and demonstrated a coordinate, dose-dependent disruption of genes involved in steroidogenesis. The gene expressions of StAR, Cyp11a1, 3β-HSD, 17β-HSD, 5α-reductase and cytochrome P450 19a1 or aromatase (Cyp19) were significantly decreased. The transcription factors, such as steroidogenic factor-1 (SF-1) and specific protein-1 (Sp-1), showed a significant decrease. DNA methylation analysis showed hypermethylation in the SF-1 and Sp-1 promoter regions. The mRNA and protein expressions of Dnmt3a, Dnmt3b, and Dnmt1 were stimulated in 10 and 100 mg DEHP treatment groups, whereas no significant change was seen in Dnmt3l expression, suggesting that increased Dnmt3a/b, Dnmt1 may cause DNA hypermethylation in testicular Leydig cells. These data indicate that gestational exposure to DEHP affects adult testicular function via altered methylation patterns.

Other environmental xenobiotics and endocrine disrupters (EDs) that interfere with the normal development of the male and female reproductive systems can act at different levels of epigenetic control. Vinclozolin (VZ) and methoxychlor (MXC) promote epigenetic transgenerational effects [189]. Polychlorinated biphenyls (PCBs), the most widespread environmental EDs, affect histone post-translational modifications in a dimorphic way, possibly as the result of an alteration of gene expression of the enzymes involved in histone modification, such as demethylase Jarid1b, an enzyme also involved in regulating the interaction of androgens with their receptor [190].

Many enzymes involved in xenobiotic metabolism, including CYP1A1, are regulated by the aryl hydrocarbon receptor (AhR); 3,3′,4,4′,5-penta chlorobiphenyl (PCB 126) is a potent ligand for AhR and can thus induce the expression of CYP1A1. Vorrink *et al.* [191] studied the epigenetic determinants of CYP1A1 induction in carcinoma cell lines. In contrast to HepG2 hepatocarcinoma cells, HeLa cervical carcinoma cells showed significantly lower levels of CYP1A1 mRNA expression following PCB 126 exposure. The two cell lines maintained differences in the chromatin architecture along the CYP1A1 promoter region. Treatment with the epigenetic modifiers trichostatin A (TSA) and 5-aza-2′-deoxycytidine (5-Aza-dC) significantly increased the expression of CYP1A1 after PCB 126 treatment in HeLa cells. No apparent differences were observed in methylation levels or the specific location of CpG DNA methylation between the two cell lines in the CYP1A1 promoter region. The differences in CYP1A1 expression between HepG2 and HeLa cells might be due to differences in the chromatin architecture of the CYP1A1 promoter and thus establish a role of epigenetic regulation in cell-specific CYP1A1 expression.

Naselli *et al.* [192] studied the polymorphisms and methylation of sites contained in the 5′ flanking region of the metabolizing enzyme CYP2E1 in correlation to its expression in both tumor and non-neoplastic liver cell lines. In treated cells, reduced DNA methylation was not consistently associated with the increase of enzyme expression. The Rsa/Pst haplotype differentially influenced CYP2E1 enzyme expression. Cells with the *VNTR A4/A4* genotype showed a reduced (20%–30%) inhibition of expression compared with the *A2/A2* genotypes. Cells with the *A2/A3* genotype showed an increased expression (25%). The A2 and A3 *CYP2E1* alleles may play a more important role in the expression of the enzyme compared with other epigenetic factors, since they are binding sites for trans-acting proteins.

CYP2E1 is a pleiotropic phase I drug-metabolizing enzyme responsible for the biotransformation of most volatile organic compounds, including toluene. Human toluene exposure increases CYP2E1 mRNA and modifies its activity in leucocytes. In the blood from tannery workers exposed to toluene, Jiménez-Garza *et al.* [193] found significant correlations between airborne levels of toluene and CYP2E1 promoter methylation, as well as IL6 promoter methylation levels. CYP2E1 promoter methylation levels were higher in toluene-exposed smokers compared to non-smokers. Significant correlations were also observed between CYP2E1 promoter methylation and GSTP1 and SOD1 promoter methylation levels.

Prenatal serotonin reuptake inhibitors (SRI) antidepressant exposure and maternal depressed mood were associated with altered neonatal CYP2E1 DNA methylation status, which, in turn, appeared to be associated with birth weight [194].

*CYP1A1* and *CYP1B1* are dioxin-inducible *CYP1s* associated with carcinogenesis in extrahepatic tissues. In the carcinogenesis of hormone-responsive tissues, the CYP1B1 level is high. Abnormal expression of these CYPs is observed in cancers that are not related to hormone response. The DNA methylation status of the CpG islands within the 5′ flanking region of the *CYP1B1* and *CYP1A1* genes in seven colorectal cancer cell lines and 40 primary colorectal cancers showed *CYP1B1* gene methylation in two cell lines (SW48 and Caco-2) and two (5%) cancers, but not in corresponding normal tissues. Treatment of the cells with 5-aza-2′-deoxycytidine revealed a clear increase in the CYP1B1 mRNA levels in SW48 and Caco-2 cells, while the amount of methylated alleles decreased. Only HT29 cells showed a clear increase in CYP1A1 mRNA, although there were no apparent differences in methylation status among these cell lines. None of these cell lines showed a significant change in mRNA levels of aryl hydrocarbon receptor (AhR) and AhR nuclear translocator (ARNT), which are known to directly activate CYP1 transcription. CpG methylation of the CYP1B1 promoter region epigenetically regulates CYP1B1 expression during the development of some colorectal cancers, and it is likely that cancers with aberrant CYP1B1 expression might show altered response to procarcinogen metabolism and chemotherapy [195].

Pregnane X receptor (PXR) is a key transcription factor that regulates drug-metabolizing enzymes such as CYP3A4, and plays important roles in intestinal first-pass metabolism. Epigenetic mechanisms are involved in the regulation of PXR/CYP3A4 pathways in colon cancer cells. PXR promoter methylation is involved in the regulation of intestinal PXR and CYP3A4 mRNA expression and might be associated with the inter-individual variability of the drug responses of colon cancer cells. DNA methylation of the CpG-rich sequence of the PXR promoter was more densely detected in low-expression cells (Caco-2, HT29, HCT116, and SW48) than in high-expression cells (LS180 and LoVo), and methylation was reversed by treatment with 5-aza-dC, in association with re-expression of PXR and CYP3A4 mRNA. PXR transcription was silenced by promoter methylation in the low-expression cells, which most likely led to downregulation of CYP3A4 transactivation. A lower level of PXR promoter methylation was observed in colorectal cancer tissues compared with adjacent normal mucosa, suggesting the upregulation of the PXR/CYP3A4 mRNAs during carcinogenesis [196].

Carcinogenic compounds such as polycyclic aromatic hydrocarbons are metabolized sequentially in two phases: in phase I, CYP1A1 catalyzes conversion into harmful hydrophilic DNA adducts, whereas in phase II, GSTT1 enables excretion via conjugation into polar electrophiles. Deletion of fetal GSTT1 significantly modifies birth weight in smokers, but no polymorphism fully accounted for fetal growth restriction. Because smoking upregulates CYP1A1 expression, Suter *et al.* [197] hypothesized that epigenetic dysregulation of placental CYP1A1 expression via alterations in DNA methylation (meCpG) may further modify fetal growth. Placental expression of multiple CYP family members among gravidae revealed a significant increase in CYP1A1 expression among smokers relative to controls. CpG sites immediately proximal to the 5′-xenobiotic response element transcription factor binding element were significantly hypomethylated among smokers, a finding that uniquely correlated with placental gene expression, indicating that *in utero* tobacco exposure significantly increases placental CYP1A1 expression in association with differential methylation at a critical xenobiotic response element.

Sexual differences are only partially attributable to hormones. Cultured male or female cells, even from embryos before sexual differentiation, differ in gene expression and sensitivity to toxins, and these differences persist in isolated primary cells. Male and female cells from Swiss Webster (CWF) mice manifest sex-distinct patterns of DNA methylation for X-ist and for cytochrome P450 (CYP; family members 1a1, 2e1m, and 7b1). Dnmt3l is differentially expressed but not differentially methylated, and Gapdh is neither differentially methylated nor expressed. *CYP* family genes differ in expression in whole-tissue homogenates and cell cultures, with female Cyp expression 2two- to 355-fold higher and Dnmt3l 12- to 32-fold higher in males. DNA methylation in the promoters of these genes is sex-dimorphic; reducing methylation differences reduces the differences in the expression of these genes one- to six-fold. Stress or estradiol alters both methylation and gene expression. As reported by Penazola *et al.* [198], sex-differential methylation may have medical effects, and different methylation patterns partially explain the sex-based differences in the expression of CYP family members and X-ist, which potentially lead to inborn differences between males and females and their different responses to chronic and acute changes.

Growth hormone (GH) exerts sex-dependent effects on the liver in many species, with many hepatic genes, most notably genes coding for CYP enzymes, being transcribed in a sex-dependent manner. Sex differences in CYP expression are most striking in rats and mice (up to 500-fold male and female differences), but are also seen, albeit to a much smaller degree, in humans, where they are an important determinant of the sex-dependence of hepatic drug and steroid metabolism. GH, via its sex-dependent temporal patterns of pituitary release, activates intracellular signaling, leading to the sexually dimorphic transcription of CYPs and other liver-expressed genes. GH-regulated transcription factor STAT5b (signal transducer and activator of transcription 5b), hepatocyte nuclear factors 3β, 4alpha and 6, and sex differences in DNA methylation and chromatin structure are involved in the sex-dependent actions of GH [199].

Lipopolysaccharide (LPS) inhibits CYP19A1 expression and 17β-estradiol (E2) production in granulosa cells (GCs). This is one of the major causes of infertility underlying postpartum uterine infections. GCs exposed to LPS transiently increased expression of the pro-inflammatory cytokine genes (*IL-1β*, *TNF-α*, *IL-6*), followed by the inhibition of CYP19A1 expression and E2 production. The transient increase in pro-inflammatory cytokines was associated with HDACs. Trichostatin A (TSA) (Table 2), a HDAC inhibitor, can attenuate LPS-induced pro-inflammatory cytokine gene expression and can prevent LPS-mediated downregulation of CYP19A1 expression and E2 in GCs [200].

Human UDP-glucuronosyltransferase (UGT) 1A10 is exclusively expressed in the intestine, contributing to presystemic first-pass metabolism. Hepatocyte nuclear factor (HNF) 1α and Sp1, as well as an intestine-specific transcription factor, caudal type homeobox (Cdx) 2, are involved in the constitutive expression of UGT1A10. UGT1A10 is not expressed in the liver, where HNF1α and Sp1 are abundantly expressed. Oda *et al.* [201] demonstrated that the CpG-rich region (−264 to + 117) around the *UGT1A10* promoter was hypermethylated (89%) in hepatocytes, whereas the *UGT1A10* promoter was hypomethylated (11%) in the epithelium of the small intestine. The methylation of the *UGT1A10* promoter by SssI methylase abrogated transactivity even with overexpressed Cdx2 and HNF1α. The *UGT1A10* promoter was highly methylated (86%) in liver-derived HuH-7 cells, where UGT1A10 is not expressed. In contrast, the *UGT1A10* promoter was hardly methylated (19%) in colon-derived LS180 cells, where UGT1A10 is expressed. Treatment with 5-aza-2′-deoxycitidine (5-Aza-dC), an inhibitor of DNA methylation, resulted in an increase in UGT1A10 expression only in HuH-7 cells. Overexpression of HNF1α and Cdx2 further increased UGT1A10 expression only in the presence of 5-Aza-dC. According to these results, DNA hypermethylation would interfere with the binding of HNF1α and Cdx2, resulting in the defective expression of UGT1A10 in human liver. Epigenetic regulation would be one of the mechanisms that determines the tissue-specific expression of UGT1A10 [201].

## 8. Epigenetics of Drug Resistance

Epigenetic modifications are associated with drug resistance. Chemotherapy resistance remains an important problem in cancer. The acquisition of drug resistance is tightly regulated by post-transcriptional regulators such as RNA-binding proteins (RBPs) and miRNAs, which change the stability and translation of mRNA encoding factors involved in cell survival, proliferation, epithelial-mesenchymal transition, and drug metabolism [202]. Alterations mediated by epigenetic mechanisms are important factors in cancer progression and in response to treatment in different types of cancer. Alterations in chromatin acetylation and DNA double-strand breaks (DSBs) in oral lichen planus (OLP) are accompanied by different responses to therapy. Patients with high levels of acetyl-histone H3 at lys9 (H3K9ac), which is associated with enhanced transcription and nuclear decondensation, failed to respond to therapy or experienced disease recurrence shortly after therapy. Similarly to H3K9ac, patients who responded poorly to therapy had increased accumulation of DNA DSB, indicating genomic instability [203]. Let-7 miRNA may be involved in the chemosensitivity of cancer cell lines *in vitro*. Lower let-7a expression was associated with epirubicin resistance in primary breast tumors. Upregulation of let-7a expression sensitized breast tumor cell lines resistant to epirubicin by enhancing cellular apoptosis *in vitro*. Lower expression of let-7a miRNA can induce chemoresistance in breast cancer by enhancing cellular apoptosis and suggests that let-7a may be used as a therapeutic target to modulate epirubicin-based chemotherapy resistance [204].

About two-thirds of all breast cancers are ERα-positive and can be treated with the antiestrogen tamoxifen, and over 30% of women treated with tamoxifen (Table 4) develop drug resistance. Aberrant DNA methylation, together with other pharmacogenetic factors [182], is thought to play a role in this resistance [205]. ERα-positive thioredoxin-related transmembrane protein (TMX)2-11-resistant cells have 4000 hypermethylated sites and ERα-negative TMX2-28-resistant cells have over 33,000. Analysis of CpG sites altered in both TMX2-11 and TMX2-28 revealed that the tamoxifen-resistant cell lines share 3000 hypermethylated and 200 hypomethylated CpGs. The *ZNF350* and *MAGED1* genes are hypermethylated in both cell lines, and treatment with 5-aza-2′-deoxycitidine causes a significant reduction in promoter methylation of both ZNF350 and MAGED1 and a corresponding increase in expression in TMX2-28 [205].

*RASSF10* is located on chromosome 11p15.2, a region that shows frequent loss of heterozygosity (LOH) in several cancer types. RASSF10 suppresses colorectal cancer growth by activating P53 signaling. RASSF10 is methylated in 82.6% of human primary hepatocellular carcinoma cells (HCC) and methylation of RASSF10 is associated with tumor size and TNM stage. The expression of RASSF10 is regulated by promoter region methylation. Restoration of RASSF10 expression suppresses cell proliferation, induces apoptosis and G2/M phase arrest, sensitizes cells to docetaxel (Table 4), and activates P53 signaling in HepG2 and QGY7703 cells [206].

The chemoresistance of pancreatic cancer to gemcitabine (Table 4) is dependent on PKM2 expression. Pyruvate kinase M2 (PKM2) is an important therapeutic target for cancer treatment. This enzyme of aerobic glycolysis is involved in the metabolic reprogramming of cancer cells and has unexpected non-metabolic functions that are heavily involved in tumor growth and survival. Knocking-down of PKM2 significantly enhances gemcitabine-induced cell apoptosis through the activation of caspase 3/7 and poly ADP ribose polymerase (PARP) cleavage, and this inhibitory activity is associated with p38-mediated activation of p53 phosphorylation at serine 46 [207]. Inhibition of miR-125b and miR-141 expression reduces cellular survival in response to taxane-anthracycline treatment in breast cancer cells. Co-transfection with miR-125b and miR-141 mimics increased resistance of (MCF7) and BT549 cells to taxane-anthracycline-induced cytotoxicity. Many of the target proteins of miR-125b are involved in apoptotic pathways and cell cycle control. Elevated miR-125b and 141 expression predicts a poor clinical responsiveness of taxane-anthracycline-based neoadjuvant chemotherapy [208].

Bromodomain and extraterminal (BET) domain proteins are promising therapeutic targets in glioblastoma and in some other types of cancer. Small-molecule inhibitors of BET bromodomain proteins reduce expression of several oncogenes required for glioblastoma multiforme (GBM) progression. Pastori *et al.* [209] identified a subset of GBM-specific lncRNAs whose expression is regulated by BET proteins. Treatment of GBM cells with the BET bromodomain inhibitor I-BET151 reduced levels of the tumor-promoting lncRNA HOX transcript antisense RNA (HOTAIR) and restored the expression of several other GBM downregulated lncRNAs. Conversely, overexpression of HOTAIR in conjunction with I-BET151 treatment abrogates the antiproliferative activity of the BET bromodomain inhibitor. Bromodomain Containing 4 (BRD4) binds to the HOTAIR promoter, suggesting that BET proteins can directly regulate lncRNA expression. BET proteins control tumor growth of glioblastoma cells and the modulation of lncRNA networks may, in part, mediate the antiproliferative effects of many epigenetic inhibitors currently under clinical trials for cancer and other diseases.

Oxidative stress is a cause of inflammation-related diseases and cancers such as cholangiocarcinoma. Hydrogen peroxide (H_2_O_2_) can generate hydroxyl radicals, which damage lipids, proteins, and nucleic acids, leading to cell death. Some cells can survive by adapting to oxidative stress conditions, and selective clonal expansion of these resistant cells would be involved in oxidative stress–related carcinogenesis. Thanan *et al.* [210] generated an H_2_O_2_-resistant cell line from an immortal cholangiocyte cell line (MMNK1) by chronic treatment with low-concentration H_2_O_2_. The resistant ox-MMNK1-L cell line showed H_2_O_2_-resistant properties, increasing the expression of the anti-oxidant genes catalase (*CAT*), superoxide dismutase-1 (*SOD1*), superoxide dismutase-2 (*SOD2*), and superoxide dismutase-3 (*SOD3*) and the enzyme activities of CAT and intracellular SODs. These resistant cells showed increased expression levels of an epigenetics-related gene, DNA methyltransferase-1 (DNMT1), when compared to the parental cells. The ox-MMNK1-L cell line had a significantly higher cell proliferation rate than the MMNK1 normal cell line.

Pharmacoresistance to antiepileptic drugs (AEDs) is a major clinical problem in patients with mesial temporal lobe epilepsy (mTLE) [211]. Levetiracetam (LEV) represents a unique type of AED as its high-affinity binding site, the synaptic vesicle protein SV2A, is a component of the presynaptic release machinery. Approximately 30% of LEV-treated mTLE patients are non-responders. This unexpected phenomenon prompted genetic studies, which failed to characterize SV2A (responsible synaptic vesicle glycoprotein 2A) sequence alterations. Transcriptome and subsequent multimodal cluster analyses uncovered strikingly abundant synapse-associated molecule mRNA signatures in LEV non-responders. The SNP rs9305614 G-allele accumulates in non-responders, correlating with mRNAs of phosphatidylinositol *N*-acetylglucosaminyltransferase (PIGP), a key component of the Wnt signaling pathway. A hyperactivation by the LBP-1 transcription factor of the rs9305614 G-allele *PIGP* promoter was observed in patients resistant to LEV treatment. These data reported by Grimminger *et al.* [212] argue for epigenetic factors predisposing for *a priori* LEV pharmacoresistance.

Studies indicate that histone deacetylation is important for long-term changes related to stress and antidepressant treatment [213]. The classic antidepressant imipramine, and ketamine, an antagonist of the *N*-methyl-d-asparte (NMDA) receptor, decrease HDAC activity in selected brain regions (nucleus accumbens) of maternally deprived adult rats [214]. Transcriptional differences in IL11 after antidepressant treatment correspond to clinical response in patients with major depressive disorder. Potential predictors of antidepressant response are the SNP rs1126757 and DNA methylation at a CpG unit predictor in IL11 [215].

**Table 4:** Pharmacological properties and pharmacogenetics of selected antineoplastic agents.

Drug	Properties	Pharmacogenetics
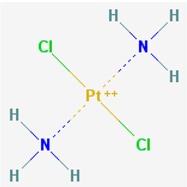	Name: Cisplatin; Cisplatinum; Lederplatin; Briplatin; Cismaplat; Cisplatine; Cisplatino; IUPAC Name: dichloroplatinumdiamine; Molecular Formula: Cl_2_H_6_N_2_Pt^2+^; Molecular Weight: 300.05104 g/mol; Category: Platinum Compounds; Mechanism: Forms platinum complexes that bind to specific DNA base sequences, producing intrastrand and interstrand DNA cross-links, which inhibit DNA replication, transcription, and cell division. Denatures the double helix and disrupts DNA function. May bind to proteins; Effect: Antineoplastic Agent; Cross-Linking Reagent; Radiation-Sensitizing Agent; Immunosuppressive effects; Antimicrobial properties	Pathogenic genes: *BRCA2*, *EGFR*, *ERBB2*, *ERCC1*, *GSTT1*, *GSTP1*, *IL6*, *MGMT*, *NQO1*, *TNF*, *TP53*, *TYMS*, *XRCC1*; Mechanistic genes: *ABCC5*, *ERBB2*, *ERCC1*, *ERCC2*, *FIS1*, *MGMT*, *MSH2*, *XPA*, *XRCC1*; Metabolic genes: Substrate: *ACSL3*, *BRCA1*, *CES2*, *CYP2E1* (major), *CYP3A4* (major), *DPYD*, *ERCC1*, *GSTA1*, *GSTM1*, *GSTO1*, *GSTP1*, *GSTT1*, *NQO1*, *SULT1A1*, *UGT1A1*, *UGT1A3*, *UGT1A6*, *XRCC1*, *XRCC3*, *XRCC4*; Inhibitor: *BCHE*, *CYP2B6* (strong), *CYP2C9* (weak); Inducer: *ABCB1*, *CYP2E1*, *CYP3A4*; Transporter genes: *ABCB1*, *ABCC2*, *ABCC1*, *ABCC3*, *ABCC4*, *ABCC5*, *ABCC8*, *ABCG2*, *ATP7A*, *TAP1*, *SLC15A1*, *SLC22A1*, *SLC22A2*; Pleiotropic genes: *ABCC3*, *ABL1*, *EDNRA*, *ERBB2*, *ERCC2*, *GGH*, *FIS1*, *FOS*, *HLA-A*, *ICAM1*, *IL1B*, *IL6*, *ITPA*, *MMP3*, *NOX1*, *NQO1*, *NR1I2*, *PRNP*, *PTGS1*, *PTGS2*, *TGFB1*, *TNF*, *TNFRSF1B*, *TPMT*, *UCP2*, *VEGFA*
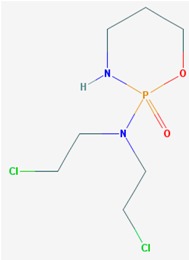	Name: Cyclophosphamide; Neosar; Cyclophosphamid; Procytox; Clafen; Cytoxan; IUPAC Name: *N*,*N*-bis(2-chloroethyl)-1-oxo-6-oxa-2-aza-1λ5-phosphacyclohexan-1-amineMolecular Formula: C_7_H_15_Cl_2_N_2_O_2_P; Molecular Weight: 261.085962 g/mol; Category: Nitrogen mustard analogues; Mechanism: Prevents cell division by cross-linking DNA strands and decreasing DNA synthesis; also possesses potent immunosuppressive activity, has phosphorylating properties which enhance its cytotoxicity; Effect: Alkylating agent; Immunosuppressive agent; Phosphorylating properties; Antirheumatic agent; Myeloablative agonist; Mutagen	Pathogenic genes: *CBR3*, *EGFR*, *ERBB2*, *ERBB4*, *ERCC1*, *ESR2*, *FOS*, *GSTP1*, *IL6*, *IL10*, *MGMT*, *MTHFR*, *MSH2*, *NQO1*, *PTGS2*, *SOD2*, *TGFB1*, *TNF*, *TP53*; Metabolic genes: Substrate: *ALDH1A1*, *ALDH2*, *ALDH3A1*, *ABCC4*, *CYP2A6* (minor), *CYP2B6* (minor), *CYP2C9* (minor), *CYP2C19* (minor), *CYP3A4* (major), *CYP1A2*, *CYP1B1*, *CYP2D6*, *GSTA1*, *GSTM1*, *GSTP1*; Inhibitor: *CYP3A4* (weak); Inducer: *ABCC4*, *CYP2B6*, *CYP2C8*, *CYP2C9*, *CYP3A4*; Transporter genes: *ABCB1*, *ABCG2*, *ABCC1*, *ABCC4*, *SLC5A5*; Pleiotropic genes: *CBR3*, *CRHR1*, *CRHR2*, *EGFR*, *ERBB2*, *ERBB4*, *ERCC1*, *ERCC2*, *ESR1*, *HTR3B*, *HTR3C*, *ICAM1*, *IL1B*, *IL1RN*, *IL4*, *IL6*, *IL10*, *IL12B*, *MAOA*, *MMP3*, *MTHFR*, *NQO1*, *SOD2*, *TGFB1*, *TNF*, *VCAM1*
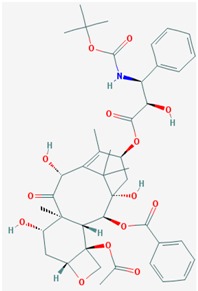	Name: Docetaxel; Taxotere; Docetaxel anhydrous; 114977-28-5; EmDOC; IUPAC Name: (1*S*,2*S*,3*R*,4*S*,7*R*,9*S*,10*S*,12*R*,15*S*)-4-(acetyloxy)-15-[[(2*R*,3*S*)-3-[[(tert-butoxy)carbonyl]amino]-2-hydroxy-3-phenylpropanoyl]oxy]-1,9,12-trihydroxy-10,14,17,17-tetramethyl-11-oxo-6-oxatetracyclo[11.3.1.03,10.04,7]heptadec-13-en-2-yl benzoate; Molecular Formula: C_43_H_53_NO_14_; Molecular Weight: 807.87922 g/mol; Category: Plant Alkaloids and Other Natural Products; Mechanism: Promotes the assembly of microtubules from tubulin dimers, and inhibits the depolymerization of tubulin which stabilizes microtubules in the cell, resulting in inhibition of DNA, RNA, and protein synthesis. Induces apoptosis in cancer cells by binding to an apoptosis-stopping protein called Bcl-2 and arresting its function; Effect: Tubulin modulator; Antineoplastic agent; Photosensitizing agent; Antimalarial	Pathogenic genes: *BRCA1*, *BRCA2*, *DPYD*, *ERBB2*, *GSTP1*, *IGF2*, *ILB1*, *IL6*, *PPARD*, *PPARG*, *PIK3CA*, *PTGS1*, *PTGS2*, *RASSF10*, *TGFBR2*, *TGFBR3*, *TNF*, *TP53*, *TYMS*, *VEGFA*, *XPC*; Mechanistic genes: *BCL2*, *EGFR*, *GHRHR*, *MAP2*, *MAP4*, *MAPK7*, *NR1I2*, *PTGS2*, *TUBB*; Metabolic genes: Substrate: *ABCB1*, *ABCC1*, *ABCC2*, *ABCC10*, *ABCG2*, *CYP1B1*, *CYP2C8* (major), *CYP3A4/5* (major), *CYP2D6*, *CYP3A7*, *CYP4B1*, *GSTMs*, *GSTP1*, *GSTTs*, *NAT2*, *SLCO1B3*, *SLC22A7*, *SULT1C2*, *UGT1A1*; Inhibitor: *ABCB1*, *CYP1B1*, *CYP3A4* (weak), *CYP19A1*; Inducer: *CYP1B1*; Transporter genes: *ABCB1*, *ABCC2*, *ABCC6*, *ABCC10*, *ABCG2*, *ATP7A*, *ABCC1*, *ABCC8*, *SLC10A2*, *SLCO1B3*; Pleiotropic genes: *ABCC6*, *ATP7A*, *EPHX1*, *ERCC2*, *HNF4A*, *IL1R2*, *IL6*, *MTHFR*, *NDUFB4*, *PPARD*, *PPARG*, *SPG7*, *TNF*, *TP53*, *TPMT*, *XPC*, *XRCC4*, *PTGES*, *VEGFA*
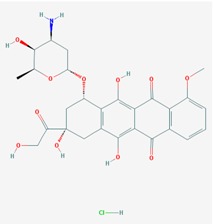	Name: Doxorubicin; Adriamycin; Doxil; Adriablastin; Rubex; Doxorubicine; IUPAC Name: (7*S*,9*S*)-7-[(2*R*,4*S*,5*S*,6*S*)-4-amino-5-hydroxy-6-methyloxan-2-yl]oxy-6,9,11-trihydroxy-9-(2-hydroxyacetyl)-4-methoxy-8,10-dihydro-7H-tetracene-5,12-dione; Molecular Formula: C_27_H_29_NO_11_; Molecular Weight: 543.51926 g/mol; Category: Cytotoxic Antibiotics and Related Substances; Mechanism: Antineoplastic action involves free radical formation secondary to metabolic activation by electron reduction, intercalation into DNA, induction of DNA breaks and chromosomal aberrations, and alterations in cell membranes induced by the drug (apoptosis may also be involved). It binds DNA and cell membranes and produce free radicals which immediately cleave the DNA and cell membranes; Effect: Topoisomerase II Inhibitor; Antibiotic	Pathogenic genes: *BRCA1*, *BRCA2*, *CCND1*, *ERBB2*, *FCGR3A*, *FOS*, *GNAS*, *GSTM1*, *GSTP1*, *IL6*, *MET*, *MLH1*, *MSH2*, *MTHFR*, *NOS3*, *NQO1*, *TGFB1*, *TNF*, *TP53*, *TYMS*, *VEGFA*; Mechanistic genes: *ABCB1*, *CAT*, *CFTR*, *ERCC2*, *ESR1*, *ESR2*, *GATA4*, *GPX1*, *GSTP1*, *MLH1*, *MGMT*, *MMP2*, *MSH2*, *NOX1*, *NFKB1*, *S0D1*, *TOP2A*, *TP53*; Metabolic genes: Substrate: *ABCB1*, *ABCC1*, *ABCG2*, *CBR1*, *CBR3*, *CYP2D6* (major), *CYP2J2*, *CYP3A4* (major), *CYP3A5*, *G6PD*, *GSTA1*, *GSTP1*, *NOS3*, *NQO1*, *NR1I2*, *SLC22A16*, *S0D1*, *XDH* Inhibitor: *CYP2B6* (moderate), *CYP2D6* (weak), *CYP3A4* (weak), *CYP2C8*, *NR1I2*; Inducer: *CYP1A1*, *CYP1A2*, *CYP1B1*, *CYP2B2*, *CYP2C11*, *CYP2E1*, *CYP2J3*, *EPHX1*; Transporter genes: *ABCB1*, *ABCC1*, *ABCC2*, *ABCC3*, *ABCC6*, *ABCG2*, *KCNH2*, *RALBP1*, *SCN5A*, *SCNN1G*, *SLC5A5*, *SLC22A16*; Pleiotropic genes: *ADRB1*, *ADRB2*, *AOX1*, *APOA1*, *APP*, *CES1*, *CES2*, *CFTR*, *CNR1*, *ERBB4*, *F7*, *FCGR3A*, *FKBP5*, *GGCX*, *GNAS*, *GSK3B*, *HFE*, *IFNA1*, *IL1B*, *IL6*, *MMP2*, *MTHFR*, *MT-ND6*, *MTR*, *NOS3*, *NPPA*, *NQO1*, *PPARA*, *PRNP*, *PROC*, *PTGS1*, *PTGS2*, *SULT1A*, *TGFB1*, *TIMP3*, *TNF*, *TNFRSF1B*, *TP53*, *VEGFA*, *XDH*
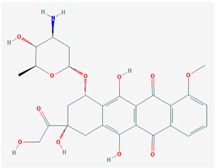	Name: Epirubicin; 4′-Epiadriamycin; Ellence; Epiadriamycin; Epidoxorubicin; 4′-epidoxorubicin; IUPAC Name: (7*S*,9*S*)-7-[(2*R*,4*S*,5*R*,6*S*)-4-amino-5-hydroxy-6-methyloxan-2-yl]oxy-6,9,11-trihydroxy-9-(2-hydroxyacetyl)-4-methoxy-8,10-dihydro-7H-tetracene-5,12-dione; Molecular Formula: C_27_H_29_NO_11_; Molecular Weight: 543.51926 g/mol; Category: Anthracycline Agent; Mechanism: Intercalates into DNA and inhibits topoisomerase II, thereby inhibiting DNA replication and, ultimately, interfering with RNA and protein synthesis. This agent also produces toxic free-radical intermediates and interacts with cell membrane lipids causing lipid peroxidation; Effect: Topoisomerase II Inhibitor; Antibiotic; Antineoplastic agent	Pathogenic genes: *CBR3*, *DPYD*, *EGFR*, *ERBB2*, *NQO1*, *MLH1*, *SOD2*; Mechanistic genes: *CHD1*, *TOP2A*; Metabolic genes: Substrate: *ABCB1*, *ABCC1*, *UGT2B7*; Inhibitor: *PLA2G4A*; Transporter genes: *ABCC1*, *ABCG2*; Pleiotropic genes: *CAT*, *HTR3B*, *HTR3C*, *NQO1*, *SOD2*
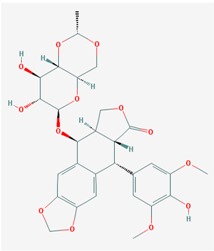	Name: Etoposide; VePesid; Lastet; Toposar; Trans-Etoposide; VP-16; IUPAC Name: (5*S*,5a*R*,8a*R*,9*R*)-5-[[(2*R*,4a*R*,6*R*,7*R*,8*R*,8a*S*)-7,8-dihydroxy-2-methyl-4,4a,6,7,8,8a-hexahydropyrano[3,2-d][1,3]dioxin-6-yl]oxy]-9-(4-hydroxy-3,5-dimethoxyphenyl)-5a,6,8a,9-tetrahydro-5H-[2]benzofuro[6,5-f][1,3]benzodioxol-8-one; Molecular Formula: C_29_H_32_O_13_; Molecular Weight: 588.55658 g/mol; Category: Podophyllotoxin Derivative; Mechanism: Stabilizes the double-stranded DNA cleavage normally catalyzed by topoisomerase II and inhibits faithful religation of DNA breaks. Delays transit of cells through the S phase and arrests cells in late S or early G2 phase. Inhibits mitochondrial transport at the NADH dehydrogenase level or inhibits uptake of nucleosides into HeLa cells; Effect: Antineoplastic agent (Phytogenic); Topoisomerase II inhibitor	Pathogenic genes: *BRCA2*, *ERBB2*, *ERBB4*, *FOS*, *GSTA1*, *GSTT1*, *GSTP1*, *IL6*, *MTHFR*, *NQO1*, *TNF*, *TP53*, *TYMS*, *VEGFA*; Mechanistic genes: *NQO1*, *TOP2A*, *TOP2B*; Metabolic genes: Substrate: *ABCB1*, *ABCC1*, *ABCC2*, *ABCC3*, *ABCC6*, *ABCG2*, *CYP1A2* (minor), *CYP2B6*, *CYP2E1* (minor), *CYP3A4/5* (major), *GSTM1*, *GSTP1*, *GSTT1*, *UGT1A1*, *VDR*; Inhibitor: *CYP2A6*, *CYP2C8*, *CYP2C9* (weak), *CYP3A4* (weak), *TOP2s*, *UGT1A3*; Inducer: *CYP3A4*, *CYP3A5*; Transporter genes: *ABCB1*, *ABCC1*, *ABCC2*, *ABCC3*, *ABCC6*, *ABCG2*, *SLC19A1*, *TAP1*; Pleiotropic genes*: ACSL3*, *ACSL5*, *AGPAT2*, *ALDH3A1*, *ERBB4*, *GSTA1*, *IL1B*, *IL6*, *MTHFR*, *NQO1*, *NR3C1*, *PRNP*, *TNF*, *TP53*, *TPMT*, *VDR*, *VEGFA*, *WNT5B*
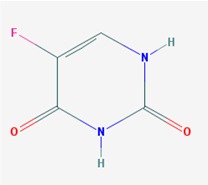	Name: Fluorouracil; 5-Fluorouracil; 5-FU; 51-21-8; Fluoroplex; Efudex; IUPAC Name: 5-fluoro-1H-pyrimidine-2,4-dione; Molecular Formula: C_4_H_3_FN_2_O_2_; Molecular Weight: 130.077223 g/mol; Category: Antimetabolites; Mechanism: Firstly, it has to be converted to its active form, 5-fluoro-2 deoxyuridine monophophate (5-FdUMP). This then interferes with DNA synthesis by binding to the enzyme thymidylate synthetase, causing it to be inactivated. The binding can be stabilized by the addition of folinic acid; Effect: Immunosuppressive agent; Antimetabolite; Antineoplastic agent; Pyrimidine antagonist	Pathogenic genes: *ALDH1A1*, *BRCA1*, *BRCA2*, *CCND1*, *CDA*, *ERBB2*, *ERBB4*, *ERCC1*, *FOS*, *GSTM1*, *GSTP1*, *GSTT1*, *IL6*, *KRAS*, *MGMT*, *MLH1*, *MMP2*, *MMP3*, *MSH2*, *MTHFR*, *PPARG*, *RB1*, *STAT3*, *TERT*, *TGFB1*, *TNF*, *TNFRSF1B*, *TP53*, *VEGFA*; Mechanistic genes: *CCND1*, *CDA*, *CDK2*, *DHFR*, *EGFR*, *ERCC2*, *FPGS*, *GGH*, *MTHFR*, *PTGS2*, *PPARG*, *RRM*, *SMUG1*, *TDG*, *TNF*, *TP53*, *TYMS*, *XRCC3*; Metabolic genes: Substrate: *ABCG2*, *CYP1A1*, *CYP2A6* (major), *DPYD*, *DPYS*, *MTHFR*, *TPMT*, *TYMS*, *UGT1A1*; Inhibitor: *CYP2C9* (strong), *CYP2C19* (strong); Inducer: *CES2*; Transporter genes: *ABCB1*, *ABCC1*, *ABCC2*, *ABCC3*, *ABCC4*, *ABCC6*, *SLC15A1*, *SLC19A1*, *SLC22A7*, *SLC22A8*, *TAP1*; Pleiotropic genes: *ADCY9*, *APC*, *BDKRB2*, *CBS*, *CDK1*, *CDKN2A*, *CES1*, *CHRNA4*, *CSNK1E*, *DCK*, *DRD5*, *EDNRA*, *EGFR*, *EPHX1*, *ERBB4*, *ERCC1*, *F2*, *FKBP5*, *GRINA*, *HBB*, *IFNA1*, *IFNB1*, *IFNG*, *IL1B*, *IL6*, *IL8RA*, *IL12B*, *IRF1*, *MAPT*, *MT-ATP6*, *MTHFR*, *NTRK2*, *POLG*, *PPARG*, *PRNP*, *PTGS2*, *RGS2*, *RGS4*, *STAT3*, *TGFB1*, *TNF*, *TOP1*, *TP53*, *VEGFA*
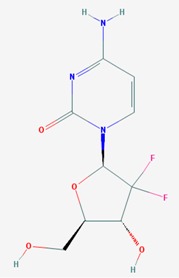	Name: Gemcitabine; 95058-81-4; Zefei; 2′,2′-difluorodeoxycytidine; Gemcitabina; Gemcitabinum; IUPAC Name: 4-amino-1-[(2*R*,4*R*,5*R*)-3,3-difluoro-4-hydroxy-5-(hydroxymethyl)oxolan-2-yl]-1,2-dihydropyrimidin-2-one; Molecular Formula: C_9_H_11_F_2_N_3_O_4_; Molecular Weight: 263.198146 g/mol; Category: Pyrimidine Analog; Mechanism: Inhibits DNA synthesis by inhibition of DNA polymerase and ribonucleotide reductase, specific for S phase of the cycle; Effect: Antimetabolite; Antineoplastic agent; Radiation-sensitizing agent; Enzyme inhibitor; Immunosuppressive agent; Antiviral agent	Pathogenic genes: *BRCA2*, *CCND1*, *CDKN2*, *EGFR*, *ERBB2*, *IL6*, *MGMT*, *MTHFR*, *PKM2*, *TERT*, *TNF*, *TP53*, *VEGFA*; Mechanistic genes: *CMPK1*, *PKM2*, *POLA2*, *RRM1*, *TYMS*; Metabolic genes: Substrate: *ABCB1*, *ABCC10*, *CDA*, *DCK*, *DCTD*, *RRM1*, *SLC28A3*, *SLC29A1*; Transporter genes: *ABCB1*, *ABCC10*, *ABCC3*, *ABCC6*, *ABCC8*, *ABCG2*, *AGPAT2*, *SLC28A1*, *SLC28A2*, *SLC28A3*, *SLC29A1*, *SLC29A2*; Pleiotropic genes: *EGFR*, *ERCC2*, *HSPA1L*, *ICAM1*, *IL6*, *MTHFR*, *NNMT*, *PKM2*, *PTGS1*, *RGS2*, *TNF*, *TOP1*, *TP53*, *USF2*, *VEGFA*, *VGF*
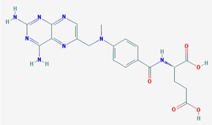	Name: Methotrexate; Amethopterin; Rheumatrex; 59-05-2; Trexall; Abitrexate; IUPAC Name: (2*S*)-2-[[4-[(2,4-diaminopteridin-6-yl)methyl-methylamino]benzoyl]amino]pentanedioic acid; Molecular Formula: C_20_H_22_N_8_O_5_; Molecular Weight: 454.43928 g/mol; Category: Antimetabolites; Mechanism: Inhibits DNA synthesis. Irreversibly binds to dihydrofolate reductase, inhibiting formation of reduced folates and thymidylate synthetase, resulting in inhibition of purine and thymidylic acid synthesis. It is a folic acid metabolism inhibitor; Effect: Antirheumatic agent; Dermatologic agent; Immunosuppressive agent; Immune modulator; Anti-inflammatory activity; Folic acid antagonist; Abortifacient agent (nonsteroidal); Nucleic acid synthesis inhibitor; Antineoplastic agent; Enzyme inhibitor	Pathogenic genes: *ALOX5*, *DPYD*, *ERBB2*, *GSTP1*, *GSTT1*, *IL1RN*, *IL6*, *MTHFR*, *NQO1*, *RB1*, *TGFB1*, *TNFRSF1B*, *TNF*, *TP53*, *VDR*; Mechanistic genes: *ADA*, *ADORA2A*, *ATIC*, *DHFR*, *FPGS*, *GGH*, *IL1B*, *IL1RN*, *IL6*, *MTHFR*, *MTR*, *PGD*, *TYMS*, *SHMT1*, *SLC19A1*, *TNF*, *TP53*; Metabolic genes: Substrate: *ABCB1*, *ABCC1*, *ABCC3*, *ABCC4*, *ABCG2*, *AOX1*, *CYP3A4* (major), *DHFR*, *FPGS*, *GGH*, *GSTM1*, *GSTP1*, *GSTT1*, *MTHFR*, *SLC19A1*, *SLC22A6*, *SLC22A8*, *SLCO1A2*, *SLCO1B1*, *SLCO1B3*, *TYMS*, *XDH*; Inhibitor: *CYP1A2*, *CYP7A1*, *DHFR*, *NAT2*, *TPMT*; Transporter genes: *ABCB1*, *ABCC1*, *ABCC2*, *ABCC3*, *ABCC4*, *ABCC10*, *ABCG2*, *SLC19A1*, *SLC22A6*, *SLC22A7*, *SLC22A8*, *SLCO1A2*, *SLCO1B1*, *SLCO1B3*; Pleiotropic genes: *ACOX1*, *ALB*, *ALOX5*, *CBS*, *COMT*, *CREB1*, *FABP1*, *FMR1*, *G6PD*, *HLA-A*, *HLA-C*, *IL1B*, *IL6*, *LIPC*, *MTHFR*, *MMP3*, *NOS3*, *NQO1*, *NR3C1*, *TGFB1*, *TNF*, *TP53*, *UGT1A1*, *UGT2B15*, *VDR*, *XDH*
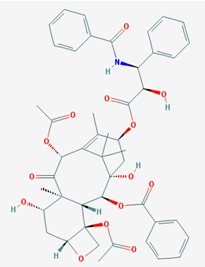	Name: Paclitaxel; Taxol; 33069-62-4; Paxene; Abraxane; Onxol; IUPAC Name: (1*S*,2*S*,3*R*,4*S*,7*R*,9*S*,10*S*,12*R*,15*S*)-4,12-bis(acetyloxy)-1,9-dihydroxy-15-[[(2*R*,3*S*)-2-hydroxy-3-phenyl-3-(phenylformamido)propanoyl]oxy}-10,14,17,17-tetramethyl-11-oxo-6-oxatetracyclo[11.3.1.03,10.04,7]heptadec-13-en-2-yl benzoate; Molecular Formula: C_47_H_51_NO_14_; Molecular Weight: 853.90614 g/mol; Category: Taxane Derivative; Mechanism: Promotes microtubule assembly, and can distort mitotic spindles, resulting in breakage of chromosomes. May also suppress cell proliferation. Induces programmed cell death in cancer cells by binding to an apoptosis-stopping protein called Bcl-2 and arresting its function; Effect: Tubulin modulator; Antineoplastic agent (phytogenic); Immune modulator	Pathogenic genes: *BRCA1*, *BRCA2*, *CCND1*, *DPYD*, *EGFR*, *ERBB2*, *ERCC1*, *GSTM1*, *GSTP1*, *GSTT1*, *MEN1*, *MGMT*, *MLH1*, *MSH2*, *MTHFR*, *NQO1*, *PTGS2*, *RB1*, *TERT*, *TLR4*, *TNF*, *TP53*, *TYMS*, *VEGFA*, *XPA*; Mechanistic genes: *BCL2*, *CASP3*, *CASP6*, *CASP8*, *CASP10*, *CDA*, *CDK2*, *MAP2*, *MAP4*, *MAPT*, *MAPK1*, *MAPK14*, *TLR4*, *TP53*, *TUBB*, *TUBB3*; Metabolic genes: Substrate: *ABCB1*, *ABCB11*, *ABCC2*, *ABCC3*, *ABCC6*, *CYP1B1* (major), *CYP2A6*, *CYP2B6*, *CYP2C8* (major), *CYP2C9* (major), *CYP2C18*, *CYP2C19*, *CYP3A4* (major), *CYP3A5*, *CYP7A1*, *CYP19A1*, *GSTM1*, *GSTT1*, *NR1I2*, *SLC22A8*, *SLC28A2*, *SLCO1B3*, *SLC22A7*; Inhibitor*: CYP1A2*, *CYP1B1*, *CYP2C8* (strong), *CYP2D6*, *CYP2D6*; Inducer: *ABCB1*, *ABCC4*, *CYP2C8*, *CYP3A4*, *NR1I2*; Transporter genes: *ABCB1*, *ABCC1*, *ABCC4*, *ABCC5*, *ABCG2*, *ATP7A*, *SLC22A8*, *SLC28A2*, *SLCO1B1*, *SLCO1B3*, *SLC22A7*; Pleiotropic genes: *AOX1*, *ATP7A*, *CAT*, *CDK1*, *ERBB4*, *ERCC1*, *FGB*, *HSPA1L*, *ICAM1*, *IL12B*, *IL17RB*, *LDLR*, *MTHFR*, *NQO1*, *PTGER4*, *TNF*, *TP53*, *TOP1*, *VCAM1*, *VEGFA*
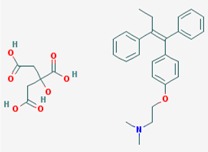	Name: Tamoxifen citrate; 54965-24-1; Istubal; ICI-46474; ICI 46474; Zitazonium; IUPAC Name: 2-[4-[(*Z*)-1,2-diphenylbut-1-enyl]phenoxy]-*N*,*N*-dimethylethanamine;2-hydroxypropane-1,2,3-tricarboxylic acid; Molecular Formula: C_32_H_37_NO_8_; Molecular Weight: 563.63808 g/mol; Category: Anti-estrogens; Mechanism: Competitively binds to estrogen receptors on tumors and other tissue targets, producing nuclear complex which decreases DNA synthesis and inhibits estrogen effects. Upregulates the production of transforming growth factor B and downregulates insulin-like growth factor 1 and protein kinase C expression in a dose-dependant manner, inhibiting signal transduction and producing an antiproliferative effect in tumors; Effect: Antineoplastic agent (hormonal); Bone density conservation agent; Estrogen antagonist; Selective estrogen receptor modulator	Pathogenic genes: *ABL1*, *BCAR1*, *BRAF*, *BRCA1*, *BRCA2*, *CCND1*, *EGFR*, *EPHX1*, *ERBB2*, *FOS*, *GRK5*, *GSTM1*, *GSTT1*, *GSTP1*, *IL6*, *KRAS*, *MMP2*, *MMP3*, *MTHFR*, *NNMT*, *NOS*, *PTGS2*, *RRAS2*, *TNF*, *TP53*, *VEGFA*; Mechanistic genes*: AR*, *BCAR1*, *ESR1*, *ESR2*, *FABP1*, *FOS*, IGF1, *GHR*, *MMP2*, *NRF1*,PRKCs, *PROC*, TGFB1, *TIMP3*, *SSTR2*; Metabolic genes: Substrate: *ABCB1*, *ABCC2*, *ABCG2*, *CYP1A1* (minor), *CYP1A2*, *CYP2A6* (minor), *CYP2B6* (minor), *CYP2C9* (major), *CYP2C19* (major), *CYP2D6* (major), *CYP2E1* (minor), *CYP3A4* (major), *CYP4B1*, *FMO1*, *FMO3*, *POR*, *UGT1A1*, *UGT1A3*, *UGT1A4*, *UGT1A9*, *UGT1A10*, *UGT2B7*, *UGT2B15*, *SULT1A1*, *SULT1E1*, *NR1I2*; Inhibitor: *ABCB1*, *ABCB11*, *CYP1B1* (moderate), *CYP2B6* (weak), *CYP2C8* (moderate), *CYP2C9* (weak), *CYP3A4* (weak), *CYP19A1* (weak), *COMT*, *GSTA1*; Inducer*: ABCB1*, *CYP3A4*, *NQO1*, *UGT1A6*; Transporter genes: *ABCB1*, *ABCB11*, *ABCC1*, *ABCC3*, *SLC14A2*, *SLC15A2*; Pleiotropic genes: *ACSL1*, *ADA*, *AGT*, *APOA1*, *APP*, *ARG1*, *AS3MT*, *CDK1*, *CFH*, *CFTR*, *CHRNA4*, *CHRNB2*, *COL1A1*, *CRHR2*, *DTNBP1*, *EPHX1*, *F7*, *FABP1*, *FMR1*, *G6PD*, *GRK5*, *HOXB13*, *HSPA1L*, *HTR2A*, *IL4*, *IL6*, *IL17RB*, *ITGB3*, *KCNH2*, *LPL*, *MAPK7*, *MMP3*, *MTHFR*, *MTTP*, *NOS3*, *NOTCH3*, *NR3C1*, *PPARGC1A*, *PROC*, *PTGER2*, *PTGER4*, *PTGES*, *RGS4*, *TNF*, *TP53*, *UCP2*, *VEGFA*

*ABCB1*: ATP-binding cassette, sub-family B (MDR/TAP), member 1; *ABCB11*: ATP-binding cassette, sub-family B (MDR/TAP), member 11; *ABCC1*: ATP-binding cassette, sub-family C (CFTR/MRP), member 1; *ABCC2*: ATP-binding cassette, sub-family C (CFTR/MRP), member 2; *ABCC3*: ATP-binding cassette, sub-family C (CFTR/MRP), member 3; *ABCC4*: ATP-binding cassette, sub-family C (CFTR/MRP), member 4; *ABCC5*: ATP-binding cassette, sub-family C (CFTR/MRP), member 5; *ABCC6*: ATP-binding cassette, sub-family C (CFTR/MRP), member 6; *ABCC8*: ATP-binding cassette, sub-family C (CFTR/MRP), member 8; *ABCC10*: ATP-binding cassette, sub-family C (CFTR/MRP), member 10; *ABCG2*: ATP-binding cassette, sub-family G (WHITE), member 2; *ABCs*: ATP-binding cassette family; *ABL1*: ABL proto-oncogene 1, non-receptor tyrosine kinase; *ACOX1*: acyl-CoA oxidase 1, palmitoyl; *ACSL1*: acyl-CoA synthetase long-chain family member 1; *ACSL3*: acyl-CoA synthetase long-chain family member 3; *ACSL5*: acyl-CoA synthetase long-chain family member 5; *ADA*: adenosine deaminase; *ADCY9*: adenylate cyclase 9; *ADORA2A*: adenosine A2a receptor; *ADRB1*: adrenoceptor β 1; *ADRB2*: adrenoceptor β 2, Surface; *AGPAT2*: 1-acylglycerol-3-phosphate *O*-acyltransferase 2; *AGT*: angiotensinogen (serpin peptidase inhibitor, clade A, member 8);*ALDH1A1*: aldehyde dehydrogenase 1 family, member A1; *ALDH2*: aldehyde dehydrogenase 2 family (mitochondrial); *ALDH3A1*: aldehyde dehydrogenase 3 family, member A1; *ALOX5*: arachidonate 5-lipoxygenase; *AOX1*: aldehyde oxidase 1; *APAF1*: apoptotic peptidase activating factor 1; *APC*: adenomatous polyposis coli; *APOA1*: apolipoprotein A-I; *APP*: amyloid β (A4) precursor protein; *AR*: androgen receptor; *ARG1*: arginase 1; *AS3MT*: arsenite methyltransferase; *ATIC*: 5-aminoimidazole-4-carboxamide ribonucleotide formyltransferase/IMP cyclohydrolase; *ATP7A*: ATPase, Cu++ transporting, alpha polypeptide; *BCAR1*: breast cancer anti-estrogen resistance 1; *BCHE*: butyrylcholinesterase; *BCL2*: B-cell CLL/lymphoma 2; *BDKRB2*: bradykinin receptor B2; *BRAF*: B-Raf proto-oncogene, serine/threonine kinase; *BRCA1*: breast cancer 1, early onset; *BRCA2*: breast cancer 2, early onset; *CASP3*: caspase 3, apoptosis-related cysteine peptidase; *CASP6*: caspase 6, apoptosis-related cysteine peptidase; *CASP8*: caspase 8, apoptosis-related cysteine peptidase; *CASP10*: caspase 10, apoptosis-related cysteine peptidase; *CAT*: catalase; *CBR1*: carbonyl reductase 1; *CBR3*: carbonyl reductase 3; *CBS*: cystathionine-β-synthase; *CCND1*: cyclin D1; *CDA*: cytidine deaminase; *CDK1*: cyclin-dependent kinase 1; *CDK2*: cyclin-dependent kinase 2; *CDKN2A*: cyclin-dependent kinase inhibitor 2A; *CDKN2B*: cyclin-dependent kinase inhibitor 2B (p15, inhibits CDK4); *CES1*: carboxylesterase 1; *CES2*: carboxylesterase 2; *CFH*: complement factor H; *CFTR*: cystic fibrosis transmembrane conductance regulator (ATP-binding cassette sub-family C, member 7); *CHD1*: chromodomain helicase DNA binding protein 1; *CHRNA4*: cholinergic receptor, nicotinic, alpha 4 (neuronal); *CHRNB2*: cholinergic receptor, nicotinic, β 2 (neuronal);*CMPK1*: cytidine monophosphate (UMP-CMP) kinase 1, cytosolic; *CNR1*: cannabinoid receptor 1 (brain); *COL1A1*: collagen, type I, alpha 1; *COMT*: catechol-*O*-methyltransferase; *CREB1*: cAMP responsive element binding protein 1; *CRHR1*: corticotropin releasing hormone receptor 1; *CRHR2*: corticotropin releasing hormone receptor 2; *CSNK1E*: casein kinase 1, epsilon; *CYP19A1*: cytochrome P450, family 19, subfamily A, polypeptide 1; *CYP1A1*: cytochrome P450, family 1, subfamily A, polypeptide 1; *CYP1A2*: cytochrome P450, family 1, subfamily A, polypeptide 2; *CYP1B1*: cytochrome P450, family 1, subfamily B, polypeptide 1; *CYP2A6*: cytochrome P450, family 2, subfamily A, polypeptide 6; *CYP2B6*: cytochrome P450, family 2, subfamily B, polypeptide 6; *CYP2B2*: cytochrome P450, family 2, subfamily B, polypeptide 2; *CYP2C11*: cytochrome P450, family 2, subfamily C, polypeptide 11; *CYP2C18*: cytochrome P450, family 2, subfamily C, polypeptide 18; *CYP2C19*: cytochrome P450, family 2, subfamily C, polypeptide 19; *CYP2C8*: cytochrome P450, family 2, subfamily C, polypeptide 8; *CYP2C9*: cytochrome P450, family 2, subfamily C, polypeptide 9; *CYP2D6*: cytochrome P450, family 2, subfamily D, polypeptide 6; *CYP2E1*: cytochrome P450, family 2, subfamily E, polypeptide 1; *CYP2J2*: cytochrome P450, family 2, subfamily J, polypeptide 2; *CYP2J3*: cytochrome P450, family 2, subfamily J, polypeptide 3; *CYP3A4*: cytochrome P450, family 3, subfamily A, polypeptide 4; *CYP3A4/5*: cytochrome P450, family 3, subfamily A, polypeptide 4/5; *CYP3A5*: cytochrome P450, family 3, subfamily A, polypeptide 5; *CYP3A7*: cytochrome P450, family 3, subfamily A, polypeptide 7; *CYP4B1*: cytochrome P450, family 4, subfamily B, polypeptide 1; *CYP7A1*: cytochrome P450, family 7, subfamily A, polypeptide 1; *DCK*: deoxycytidine kinase; *DCTD*: dCMP deaminase; *DHFR*: dihydrofolate reductase; *DNMT1*: DNA (cytosine-5-)-methyltransferase 1; *DNMT3A*: DNA (cytosine-5-)-methyltransferase 3 alpha; *DNMT3B*: DNA (cytosine-5-)-methyltransferase 3 β; *DPYD*: dihydropyrimidine dehydrogenase; *DPYS*: dihydropyrimidinase; *DRD5*: dopamine receptor D5; *DTNBP1*: dystrobrevin binding protein 1; *EDNRA*: endothelin receptor type A; *EGFR*: epidermal growth factor receptor; *EPHX1*: epoxide hydrolase 1, microsomal (xenobiotic); *ERBB2*: erb-b2 receptor tyrosine kinase 2; *ERBB4*: erb-b2 receptor tyrosine kinase 4; *ERCC1*: excision repair cross-complementation group 1; *ERCC2*: excision repair cross-complementation group 2; *ESR1*: estrogen receptor 1; *ESR2*: estrogen receptor 2 (ER β); *F2*: coagulation factor II (thrombin); *F7*: coagulation factor VII (serum prothrombin conversion accelerator); *FABP1*: fatty acid binding protein 1, liver; *FCGR3A*: Fc fragment of IgG, low affinity IIIa, receptor (CD16a); *FGB*: fibrinogen β chain; *FIS1*: fission, mitochondrial 1; *FKBP5*: FK506 binding protein 5; *FMO1*: flavin containing monooxygenase 1; *FMO3*: flavin containing monooxygenase 3; *FMR1*: fragile X mental retardation 1; *FOS*: FBJ osteosarcoma oncogene; *FPGS*: folylpolyglutamate synthase; *G6PD*: glucose-6-phosphate dehydrogenase; *GATA4*: GATA binding protein 4; *GFR*: Rap guanine nucleotide exchange factor (GEF) 5; *GGCX*: gamma-glutamyl carboxylase; *GGH*: gamma-glutamyl hydrolase (conjugase, folylpolygammaglutamyl hydrolase); *GHR*: growth hormone receptor; *GHRHR*: growth hormone releasing hormone receptor; *GNAS*: GNAScomplex locus; *GPX1*: glutathione peroxidase 1; *GRINA*: glutamate receptor, ionotropic, *N*-methyl d-aspartate-associated protein 1 (glutamate binding); *GRK5*: G protein-coupled receptor kinase 5; *GSK3B*: glycogen synthase kinase 3 β; *GSTA1*: glutathione *S*-transferase alpha 1; *GSTM1*: glutathione *S*-transferase mu 1; *GSTO1*: glutathione *S*-transferase omega 1; *GSTP1*: glutathione *S*-transferase pi 1; *GSTT1*: glutathione *S*-transferase theta 1; *HBB*: hemoglobin, β; *HBG1*: hemoglobin, γ A; *HFE*: hemochromatosis; *HLA-A*: major histocompatibility complex, class I, A; *HLA-CIL1B*: major histocompatibility complex, class II, DR β 1; *HNF4A*: hepatocyte nuclear factor 4, α; *HOXB13*: homeobox B13; *HSPA1L*: heat shock 70kDa protein 1-like; *HTR2A*: 5-hydroxytryptamine (serotonin) receptor 2A, G protein-coupled; *HTR3B*: 5-hydroxytryptamine (serotonin) receptor 3B, ionotropic; *HTR3C*: 5-hydroxytryptamine (serotonin) receptor 3C, ionotropic; *ICAM1*: intercellular adhesion molecule 1; *IFNA1*: interferon, alpha 1; *IFNB1*: interferon, β 1, fibroblast; *IFNG*: interferon, gamma; *IGF1*: insulin-like growth factor 1; *IGF2*: insulin-like growth factor 2; *IL10*: interleukin 10; *IL12B*: interleukin 12B; *IL17RB*: interleukin 17 receptor B; *IL1B*: interleukin 1, β; *IL1R2*: interleukin 1 receptor, type II; *IL1RN*: interleukin 1 receptor antagonist; *IL4*: interleukin 4; *IL6*: interleukin 6; *IL8RA*: chemokine (C-X-C motif) receptor 2; *IRF1*: interferon regulatory factor 1; *ITGB3*: integrin, β 3 (platelet glycoprotein IIIa, antigen CD61; *ITPA*: inosine triphosphatase (nucleoside triphosphate pyrophosphatase); *KCNH2*: potassium channel, voltage gated eag related subfamily H, member 2; *KRAS*: Kirsten rat sarcoma viral oncogene homolog; *LDLR*: low density lipoprotein receptor; *LIPC*: lipase, hepatic; *LPL*: lipoprotein lipase; *MAOA*: monoamine oxidase A; *MAPK1*: mitogen-activated protein kinase 1; *MAPK7*: mitogen-activated protein kinase 7; *MAPK14*: mitogen-activated protein kinase 14; *MAP2*: microtubule-associated protein 2; *MAP4*: microtubule-associated protein 4; *MAPT*: microtubule-associated protein tau; *MEN1*: multiple endocrine neoplasia I; *MET*: MET proto-oncogene, receptor tyrosine kinase; *MGMT*: *O*-6-methylguanine-DNA methyltransferase; *MLH1*: mutL homolog 1; *MMP2*: matrix metallopeptidase 2; *MMP3*: matrix metallopeptidase 3; *MMP9*: matrix metallopeptidase 9; *MSH2*: mutS homolog 2; *MT-ATP6*: mitochondrially encoded ATP synthase 6; *MTHFR*: methylenetetrahydrofolate reductase (NAD(P)H); *MT-ND6*: mitochondrially encoded NADH dehydrogenase 6; *MTR*: 5-methyltetrahydrofolate-homocysteine methyltransferase; *MTTP*: microsomal triglyceride transfer protein; *MYC*: v-myc avian myelocytomatosis viral oncogene homolog; *NAT2*: *N*-acetyltransferase 2 (arylamine *N*-acetyltransferase); *NDUFB4*: NADH dehydrogenase (ubiquinone) 1 β subcomplex, 4, 15 kDa; *NNMT*: nicotinamide *N*-methyltransferase; *NOS3*: nitric oxide synthase 3 (endothelial cell); *NOTCH3*: notch 3; *NOX1*: NADPH oxidase 1; *NPPA*: natriuretic peptide A; *NQO1*: NAD(P)H dehydrogenase, quinone 1; *NR1I2*: nuclear receptor subfamily 1, group I, member 2; *NR3C1*: nuclear receptor subfamily 3, group C, member 1 (glucocorticoid receptor); NRF1: nuclear respiratory factor 1; *NTRK2*: neurotrophic tyrosine kinase, receptor, type 2; *PIK3CA*: phosphatidylinositol-4,5-bisphosphate 3-kinase, catalytic subunit alpha; *PLA2G4A*: phospholipase A2, group IVA (cytosolic, calcium-dependent); *PKM2*: Pyruvate kinase M2; *POLA2*: polymerase (DNA directed), alpha 2, accessory subunit; *POLG*: polymerase (DNA directed), gamm; *POR*: P450 (cytochrome) oxidoreductase; *PPARA*: peroxisome proliferator-activated receptor alpha; *PPARD*: peroxisome proliferator-activated receptor delta; *PPARG*: peroxisome proliferator-activated receptor γ; *PPARGC1A*: peroxisome proliferator-activated receptor gamma, coactivator 1 α; *PRDX4*: peroxiredoxin 4; *PRNP*: prion protein; *PROC*: protein C (inactivator of coagulation factors Va and VIIIa); *PTGER2*: prostaglandin E receptor 2 (subtype EP2), 53kDa; *PTGER4*: prostaglandin E receptor 4 (subtype EP4); *PTGES*: prostaglandin E synthase; *PTGS1*: prostaglandin-endoperoxide synthase 1 (prostaglandin G/H synthase and cyclooxygenase); *PTGS2*: prostaglandin-endoperoxide synthase 2 (prostaglandin G/H synthase and cyclooxygenase); *PRCKS*: protein kinase C family; *RASSF10*: Ras association (RalGDS/AF-6) domain family (*N*-terminal) member 10; *RALBP1*: ralA binding protein 1; *RB1*: retinoblastoma 1; *RGS2*: regulator of G-protein signaling 2; *RGS4*: regulator of G-protein signaling 4; *RRAS2*: related RAS viral (r-ras) oncogene homolog 2; *RRM1*: ribonucleotide reductase M1; *SCN5A*: sodium channel, voltage gated, type V α subunit; *SCNN1G*: sodium channel, non voltage gated 1 γ subunit; *SHMT1*: serine hydroxymethyltransferase 1 (soluble); *SLC10A2*: solute carrier family 10 (sodium/bile acid cotransporter), member 2; *SLC14A2*: solute carrier family 14 (urea transporter), member 2; *SLC15A1*: solute carrier family 15 (oligopeptide transporter), member 1; *SLC15A2*: solute carrier family 15 (oligopeptide transporter), member 2; *SLC15s*: solute carrier family 15 (oligopeptide transporter); *SLC19A1*: solute carrier family 19 (folate transporter), member 1; *SLC22A1*: solute carrier family 22 (organic cation transporter), member 1; *SLC22A2*: solute carrier family 22 (organic cation transporter), member 2; *SLC22A6*: solute carrier family 22 (organic anion transporter), member 6; *SLC22A7*: solute carrier family 22 (organic anion transporter), member 7; *SLC22A8*: solute carrier family 22 (organic anion transporter), member 8; *SLC22A16*: solute carrier family 22 (organic cation/carnitine transporter), member 16; *SLC22s*: solute carrier family 22; *SLC28A1*: solute carrier family 28 (concentrative nucleoside transporter), member 1; *SLC28A2*: solute carrier family 28 (concentrative nucleoside transporter), member 2; *SLC28A3*: solute carrier family 28 (concentrative nucleoside transporter), member 3; *SLC29A1*: solute carrier family 29 (equilibrative nucleoside transporter), member 1; *SLC29A2*: solute carrier family 29 (equilibrative nucleoside transporter), member 2; *SLC29As*: solute carrier family 29; *SLC5A5*: solute carrier family 5 (sodium/iodide cotransporter), member 5; *SLCO1A2*: solute carrier organic anion transporter family, member 1A2; *SLCO1B1*: solute carrier organic anion transporter family, member 1B1; *SLCO1B3*: solute carrier organic anion transporter family, member 1B3; *SMUG1*: single-strand-selective monofunctional uracil-DNA glycosylase 1; *SOD1*: superoxide dismutase 1, soluble; *SOD2*: superoxide dismutase 2, mitochondrial; *SPG7*: spastic paraplegia 7 (pure and complicated autosomal recessive); *SSTR2*: somatostatin receptor 2; *STAT3*: signal transducer and activator of transcription 3 (acute-phase response factor); *SULT1A1*: sulfotransferase family, cytosolic, 1A, phenol-preferring, member 1; *SULT1C2*: sulfotransferase family, cytosolic, 1C, member 2; *SULT1E1*: sulfotransferase family 1E, estrogen-preferring, member 1; *TAP1*: transporter 1, ATP-binding cassette, sub-family B (MDR/TAP); *TDG*: thymine DNA glycosylase; *TERT*: telomerase reverse transcriptase; *TGFB1*: transforming growth factor, β 1; *TGFBR2*: transforming growth factor, β receptor II (70/80kDa); *TGFBR3*: transforming growth factor, β receptor III; *TIMP3*: TIMP metallopeptidase inhibitor 3; *TLR4*: toll-like receptor 4; *TNF*: tumor necrosis factor; *TNFRSF1B*: tumor necrosis factor receptor superfamily, member 1B; *TOP1*: topoisomerase (DNA) I; *TOP2A*: topoisomerase (DNA) II alpha; *TOP2B*: topoisomerase (DNA) II β; *TOP2s*: topoisomerase (DNA) II family; *TP53*: tumor protein p53; *TPMT*: thiopurine S-methyltransferase; *TUBB*: tubulin, β class I; *TUBB3*: tubulin, β 3 class III; *TYMS*: thymidylate synthetase; *UCP2*: uncoupling protein 2 (mitochondrial, proton carrier); *UGT1A1*: UDP glucuronosyltransferase 1 family, polypeptide A1; *UGT1A10*: UDP glucuronosyltransferase 1 family, polypeptide A10; *UGT1A3*: UDP glucuronosyltransferase 1 family, polypeptide A3; *UGT1A4*: UDP glucuronosyltransferase 1 family, polypeptide A4; *UGT1A6*: UDP glucuronosyltransferase 1 family, polypeptide A6; *UGT1A9*: UDP glucuronosyltransferase 1 family, polypeptide A9; *UGT2B15*: UDP glucuronosyltransferase 2 family, polypeptide B15; *UGT2B7*: UDP glucuronosyltransferase 1 family, polypeptide A7; *USF2*: upstream transcription factor 2, c-fos interacting; *VCAM1*: vascular cell adhesion molecule 1; *VDR*: vitamin D (1,25- dihydroxyvitamin D3) receptor; *VEGFA*: vascular endothelial growth factor A; *VGF*: VGF nerve growth factor inducible; *VHL*: von Hippel-Lindau tumor suppressor, E3 ubiquitin protein ligase; *WNT5B*: wingless-type MMTV integration site family, member 5B; *XDH*: xanthine dehydrogenase; *XPA*: xeroderma pigmentosum, complementation group A; *XPC*: xeroderma pigmentosum, complementation group C; *XRCC1*: X-ray repair complementing defective repair in Chinese hamster cells 1; *XRCC3*: X-ray repair complementing defective repair in Chinese hamster cells 3; *XRCC4*: X-ray repair complementing defective repair in Chinese hamster cells 4.

Compound 60 (Cpd-60) is a slow-binding, benzamide-based inhibitor of the class I histone deacetylase (HDAC) family members, HDAC1 and HDAC2. Cpd-60 treatment was associated with attenuated locomotor activity following acute amphetamine challenge. Selective inhibition of HDAC1 and HDAC2 in the brain was postulated as an epigenetic-based target for developing novel treatments for mood disorders [216].

Epigenetic changes may also explain some toxic effects of conventional drugs. Fluoroquinolones (FQ) are broad-spectrum antibiotics which may cause renal damage and tendinopathies. FQ drugs (norfloxacin, ciprofloxacin, and enrofloxacin) are powerful iron chelators comparable to deferoxamine, a clinically useful iron chelating agent. Iron chelation by FQ leads to epigenetic effects through the inhibition of α-ketoglutarate-dependent dioxygenases that require iron as a co-factor. These antibiotics inhibit TET DNA demethylases, Jumonji domain histone demethylases, and collagen prolyl 4-hydroxylases, leading to accumulation of methylated DNA and histones, and inhibition of proline hydroxylation in collagen, respectively. These epigenetic changes might explain FQ-induced nephrotoxicity and tendinopathy [217].

Epigenetic changes in drug transporters may affect drug metabolism and drug resistance. The Multidrug Resistance 1 (*MDR1*, *ABCB1*) gene product P-glycoprotein (P-gp), an ATP-binding cassette transporter, extrudes multiple endogenous and exogenous substrates from the cell, playing an important role in normal physiology and xenobiotic distribution and bioavailability. The placenta and fetal brain are barrier sites that express P-gp and play a critical role of protection of the fetus and the fetal brain from maternally administered drugs and other xenobiotics [218]. *ABCB1* has several binding sites in its promoter region, along with CpG islands and GC boxes, involved in its epigenetic control. Leucine-Rich Pentatricopeptide Repeat Containing (LRPPRC) is a potential regulator of *ABCB1* transcription via an invMED1 binding site in *ABCB1*. This invMED1 binding site overlaps with the GC-100 box. LRPPRC binds prominently to the *ABCB1* promoter in Lucena cells, an imatinib mesylate (IM)-resistant cell line. *ABCB1* transcription is positively regulated by LRPPRC upon its knockdown. *ABCB1* promoter is differentially methylated at its GC-100 box in K562 cells compared with Lucena cells, and in chronic myeloid leukemia (CML) patients with different response to IM. Chromatin immunoprecipitation and Pgp expression after DNA demethylation treatment show that LRPPRC binding is affected by the methylation status of the *ABCB1* GC-100 box. LRPPRC is a transcription factor related to *ABCB1* expression and highlights the importance of epigenetic regulation in CML resistance [219].

Yang *et al.* [220] characterized a novel ATPase protein associated with ATP-binding cassette (ABC) transporters (PAAT). PAAT contains a nucleotide-binding domain (NBD)-like domain and a signal for intramitochondrial sorting. PAAT has an intrinsic ATPase activity and is localized in both the cytoplasm and the mitochondria. PAAT interacts with mitochondrial inner-membrane ABC proteins, ABCB7, ABCB8, and ABCB10, but not with ABCB1, ABCB6, or ABCG2, and regulates the transport of ferric nutrients and heme biosynthesis. PAAT is a novel ATPase and a trans-regulator of mitochondrial ABC transporters that plays an important role in the maintenance of mitochondrial homeostasis and cell survival. Its deficiency promotes cell death, reduces mitochondrial potential, and sensitizes mitochondria to oxidative stress-induced DNA damage.

Three CpG islands within a 1.15 kb region characterize the chromatin landscape surrounding the transcriptional start site of the multidrug resistance 1 (*MDR1*) gene. Hypermethylation of this region is correlated with *MDR1* gene silencing and the inability of chemotherapeutic agents to activate MDR1 transcription [221].

Induced expression of the ABCB1 drug transporter often occurs in tumors in response to chemotherapy. Acquisition of resistance to epirubicin or paclitaxel (Table 4), with increased *ABCB1* transcript expression, is associated with *ABCB1* promoter hypomethylation. Treatment of control MCF-7 cells with demethylating and/or acetylating agents increases *ABCB1* transcript expression. Reductions in the methylation of specific CpG sites within the promoter are observed, suggesting that these sites may play a predominant role in transcriptional activation through promoter hypomethylation. Allele-specific reductions in *ABCB1* promoter methylation regulate promoter usage within paclitaxel-resistant cells. Changes in *ABCB1* promoter methylation, *ABCB1* promoter usage and *ABCB1* transcript expression can be temporally and causally correlated with the acquisition of drug resistance in breast tumor cells [222].

*MDR1* promoter methylation is a frequent finding in prostate carcinoma. In prostate carcinogenesis, *MDR1* downregulation is mainly due to histone post-translational modifications, concomitant with aberrant promoter methylation, leading to decreased expression of P-gp. Histone active marks H3Ac, H3K4me2, H3K4me3, H3K9Ac, and H4Ac were increased at the *MDR1* promoter after exposure to trichostatin A alone or combined with 5-aza-2′-deoxycytidine [223].

Efflux pumps of the ABC transporter family are subject to miRNA-mediated gene regulation. ABC transporters are embedded in a concerted and miRNA-guided network of concurrently regulated proteins that mediate altered drug transport and cell survival under changing environmental conditions. miR-27a, miR-137, miR-145, miR-200c, miR-298, miR-331-5p, miR-451, and miR-1253 are associated with reduced ABCB1 expression, and miR-27a, miR-138, miR-296, and miR-451 are associated with increased ABCB1 expression [224].

Resistance to chemotherapy may arise due to promoter methylation/downregulation of the expression of transporters required for drug uptake. In specific cases, decitabine can reverse resistance *in vitro* with changes in the expression of the endocytosis regulator RhoA, the folate carriers FOLR1 (folate receptor 1) and RFC1 (replication factor C subunit 1), and the glucose transporter GLUT4 (solute carrier family 2 (facilitated glucose transporter), member 4) [225].

Synaptically released l-glutamate, the most important excitatory neurotransmitter in the central nervous system (CNS), is removed from extracellular space by fast and efficient transport mediated by several transporters (EAAT1/GLAST and EAAT2/GLT1). There is one CpG island in the *SLC1A2* (*EAAT2/GLT1*) gene and none in *SLC1A3* (EAAT1/GLAST), and there are targets for specific miRNA in the *SLC1A2* (*EAAT2*/*GLT1*) gene [226].

Organic cation transporter 3 (OCT3, SLC22A3) mediates the uptake of endogenous amines and basic drugs in several tissues. *OCT3* is a risk locus for prostate cancer, and is markedly underexpressed in aggressive prostate cancers. Haplotypes with the common variants g.-81G>delGA (rs60515630) (minor allele frequency of 11.5% in African Americans) and g.-2G>A (rs555754) (minor allele frequency >30% in all ethnic groups) show significant increases in luciferase reporter activities and exhibit stronger transcription factor–binding affinity than haplotypes containing the major alleles. *OCT3* messenger RNA expression levels are higher in Asian and Caucasian livers from subjects homozygous for g.-2A/A as compared to those homozygous for the g.-2G/G allele. *OCT3* promoter methylation associates with *OCT3* expression level and tumorigenesis in prostate cancer cells. The methylation level of the *OCT3* promoter is higher in over 60% of prostate tumor samples. Polymorphic variants in the proximal promoter region of *OCT3* modify the transcription rate of the gene and may be associated with altered expression levels of *OCT3* in human liver. Aberrant methylation may reduce expression of *OCT3* in prostate cancer [227].

Despite alteration of DNA methylation or histone modifications, deregulated miRNA expression patterns of tumor cells have been identified as interfering with drug response [228]. Therefore, miRNAs are also involved in the mechanisms of chemoresistance. The bladder cancer (BCa) cell line 5637 is significantly more sensitive to the cytoxicity of five chemotherapeutic agents than resistant cell line (H-bc) cells. The inhibitor of growth 5 (*ING5*) gene is upregulated in 5637 cells compared with H-bc cells, indicating that it has an inhibitory role in BCa chemoresistance. siRNA-mediated inhibition of ING5 increases the chemoresistance and inhibits the DNA damage response pathway in 5637 cells. Conversely, forced expression of EGFP-ING5 decreased the chemoresistance of and activated the DNA damage response pathway in H-bc cells. *ING5* gene expression is inhibited by miR-193a-3p and is instrumental in the role of miR-193a-3p in activating BCa chemoresistance [229].

Lung cancer cells show inherent and acquired resistance to chemotherapy. El-Awady *et al.* [230] used an isogenic pair of lung adenocarcinoma cell lines (A549 (wild type) and A549DOX11 (doxorubicin-resistant)) (Table 4) to study the role of epigenetics and miRNA in the resistance/response of non-small cell lung cancer (NSCLC) cells to doxorubicin. The level of HDACs 1, 2, 3 and 4, DNA methyltransferase, acetylated H2B and acetylated H3 were lower in A549DOX11 compared to A549 cells. miRNAs were dysregulated in A549DOX11 cells compared to A549 cells; among 14 dysregulated miRNAs, four (has-mir-1973, 494, 4286 and 29b-3p) showed a 2.99- to 4.44-fold increase in their expression. This was associated with reduced apoptosis and higher resistance of A549DOX11cells to doxorubicin and etoposide. Sequential treatment with the epigenetic modifiers trichostatin A or 5-aza-2′-deoxycytidine followed by doxorubicin resulted in enhanced sensitivity of both cell lines to doxorubicin, enhanced doxorubicin-induced DNA damage in both cell lines, and dysregulation of some miRNAs in A549 cells. A549DOX11 cells resistant to DNA-damaging drugs have an epigenetic profile and miRNA expression different from the sensitive cells. Epigenetic modifiers may reverse the resistance of certain NSCLC cells to DNA-damaging agents by enhancing induction of DNA damage.

The mitochondrial fission protein FIS1 is upregulated upon cisplatin treatment (Table 4) in tongue squamous cell carcinoma (TSCC) cells. FIS1 knockdown can attenuate mitochondrial fission and cisplatin sensitivity. FIS1 is a direct target of miR-483-5p and miR-483-5p can inhibit mitochondrial fission and cisplatin sensitivity *in vitro* and *in vivo*. miR-483-5p and FIS1 are significantly associated with cisplatin sensitivity and with overall survival in patients with TSCC. These results reported by Fan *et al.* [231] reveal that a novel mitochondrial fission pathway composed of miR-483-5p and FIS1 regulates cisplatin sensitivity, and indicate that the modulation of miR-483-5p and FIS1 levels may provide a new approach for increasing cisplatin sensitivity.

## 9. Conclusions

(i) Epigenetic changes (DNA methylation, histone remodeling, miRNA regulation) are common phenomena in physiological and pathological conditions.
(ii) Epigenetic variation is sex- and age-dependent, and affects life expectancy and longevity.
(iii) Genes associated with the pathogenesis of neurodegeneration in Alzheimer’s disease exhibit epigenetic changes, suggesting that epigenetics might contribute to the pathogenesis of dementia.
(iv) DNA methylation influences phenotype differences, such as susceptibility to certain diseases and pathogens, and response to drugs and xenobiotic agents.
(v) Epigenetic modifications are associated with drug resistance.
(vi) Epigenetic modifications are reversible and can be potentially targeted by pharmacological and dietary interventions.
(vii) Epigenetic drugs can reverse epigenetic changes in gene expression and might open future avenues for the treatment of major problems of health.
(viii) A series of epigenetic drugs have been developed, including DNA methyltransferase inhibitors (nucleoside analogs, small molecules, bioproducts, antisense oligonucleotides, miRNAs), histone deacetylase inhibitors (short-chain fatty acids, hydroxamic acids, cyclic peptides, benzamides, ketones, sirtuin inhibitors, sirtuin activators), histone acetyltransferase modulators, histone methyltransferase inhibitors, histone demethylase inhibitors, and non-coding RNAs (miRNAs), with potential effects against major problems of health. Some epigenetic drugs have been approved for the treatment of different modalities of cancer.
(ix) Pharmacoepigenomics deals with the influence that epigenetic alterations may exert on genes involved in the pharmacogenomic network responsible for the pharmacokinetics and pharmacodynamics of drugs (efficacy and safety), as well as the effects that drugs may have on the epigenetic machinery.
(x) Genes involved in the pharmacogenomic process include pathogenic, mechanistic, metabolic, transporter, and pleiotropic genes which are susceptible to epigenetic modifications leading to altered expression of proteins and enzymes, with the consequent effects on the therapeutic outcome.
(xi) Although the information available at present on the pharmacoepigenomics of most drugs is very limited, growing evidence indicates that epigenetic changes are determinant in the pathogenesis of many medical conditions and in drug response and drug resistance; consequently, pharmacoepigenetic studies should be incorporated in the future as routine procedures for the proper evaluation of efficacy and safety issues in drug development and clinical trials.
